# Molecular Phylogeny of Echiuran Worms (Phylum: Annelida) Reveals Evolutionary Pattern of Feeding Mode and Sexual Dimorphism

**DOI:** 10.1371/journal.pone.0056809

**Published:** 2013-02-14

**Authors:** Ryutaro Goto, Tomoko Okamoto, Hiroshi Ishikawa, Yoichi Hamamura, Makoto Kato

**Affiliations:** 1 Department of Marine Ecosystem Dynamics, Atmosphere and Ocean Research Institute, The University of Tokyo, Kashiwa, Chiba, Japan; 2 Graduate School of Human and Environmental Studies, Kyoto University, Kyoto, Japan; 3 Uwajima, Ehime, Japan; 4 Kure, Hiroshima, Japan; Australian Museum, Australia

## Abstract

The Echiura, or spoon worms, are a group of marine worms, most of which live in burrows in soft sediments. This annelid-like animal group was once considered as a separate phylum because of the absence of segmentation, although recent molecular analyses have placed it within the annelids. In this study, we elucidate the interfamily relationships of echiuran worms and their evolutionary pattern of feeding mode and sexual dimorphism, by performing molecular phylogenetic analyses using four genes (18S, 28S, H3, and COI) of representatives of all extant echiuran families. Our results suggest that Echiura is monophyletic and comprises two unexpected groups: [Echiuridae+Urechidae+Thalassematidae] and [Bonelliidae+Ikedidae]. This grouping agrees with the presence/absence of marked sexual dimorphism involving dwarf males and the paired/non-paired configuration of the gonoducts (genital sacs). Furthermore, the data supports the sister group relationship of Echiuridae and Urechidae. These two families share the character of having anal chaetae rings around the posterior trunk as a synapomorphy. The analyses also suggest that deposit feeding is a basal feeding mode in echiurans and that filter feeding originated once in the common ancestor of Urechidae. Overall, our results contradict the currently accepted order-level classification, especially in that Echiuroinea is polyphyletic, and provide novel insights into the evolution of echiuran worms.

## Introduction

Echiurans (spoon worms or innkeeper worms) are a group of marine worms with a sausage-shaped non-segmented body and a highly extensible scoop-like proboscis [1]–[3]. Approximately 165 species of echiurans have been described in the shallow to deep waters of all oceans [4], [5]. Most live in burrows in soft sediments, although some live in crevices in dead corals or rocks [1]–[3]. In general, they are deposit feeders that collect small organic particles by scooping the sediment with their flattened and elongated proboscis, although some are filter feeders that create U-shaped burrows and gather food by generating water currents using their peristaltic bodies [1]–[3].

In recent years, much attention has been paid to the systematic position of echiuran worms [6]–[8]. They were considered to have a close phylogenetic relationship with annelids, based on their developmental and morphological characters [1]–[3]. However, echiurans can be distinguished from the other annelids by their lack of segmentation [1]–[3]. Thus, this animal group has been recognized as a separate phylum [1]. However, recent molecular phylogenetic analyses have consistently suggested that echiurans should be placed within the phylum Annelida [6]–[8]. Thus, they are currently treated as a group of derived annelids [7], [8].

In contrast to their systematic position within the annelids, the interfamily relationships of echiurans remain poorly understood [3]. The Echiura has traditionally been classified into the three orders: Echiuroinea (with Echiuridae, Thalassematidae, and Bonelliidae), Xenopneusta (Urechidae), and Heteromyota (with Ikedidae) [3]. This classification is based on their morphology, such as the arrangements of the body wall musculature, the vascular system, and the hind gut [1]–[3]. However, this information is insufficient for understanding the phylogenetic relationships among these groups [3].

It has also been suggested that the currently accepted classification should be revised [9]. The order Heteromyota was originally established for the species *Ikeda taenioides* (Ikeda, 1904), which is endemic to Japan [10]. The body-wall muscle layer of *I. taenioides* was once described as including outer longitudinal and inner circular layers, which is the opposite of that found in all other echiurans [10]. Based on this character, the order Heteromyota was proposed for this species [10]. However, a recent detailed assessment of *I. taenioides* specimens revealed that the arrangements of their muscle layers are not different from the other echiurans [9]. Thus, it was proposed that the order Heteromyota should be abolished and that the family Ikedidae should be treated as a junior synonym of the family Echiuridae [9]. According to this classification, the echiurans comprise the two orders: Echiuroinea and Xenopneusta [9].

It is also interesting that echiuran worms include species with and without marked sexual dimorphisms [1]–[3]. The members of the family Bonelliidae show marked sexual dimorphism [1]–[3]; very tiny dwarf males are parasitic on or within the female body. Similar forms of sexual dimorphism were also suggested in the Ikedidae [9]. By contrast, there is no marked sexual dimorphism in the families Echiuridae, Thalassematidae, and Urechidae [1]–[3]. How marked sexual dimorphism evolved in this animal group is an intriguing question.

In order to understand the evolutionary pattern of feeding mode and sexual dimorphism in echiuran worms, and to resolve taxonomic confusion, we constructed the molecular phylogenetic tree of echiuran worms using two nuclear ribosomal genes (18S and 28S rRNA), one nuclear protein gene (histone H3) and one mitochondrial protein gene (cytochrome *c* oxidase subunit I, COI). Representatives of all extant echiuran families were included in the analyses. We discussed the classification and character evolution of echiuran worms based on the tree produced.

## Results and Discussion

### Molecular phylogenetic analysis

**Figure 1:**
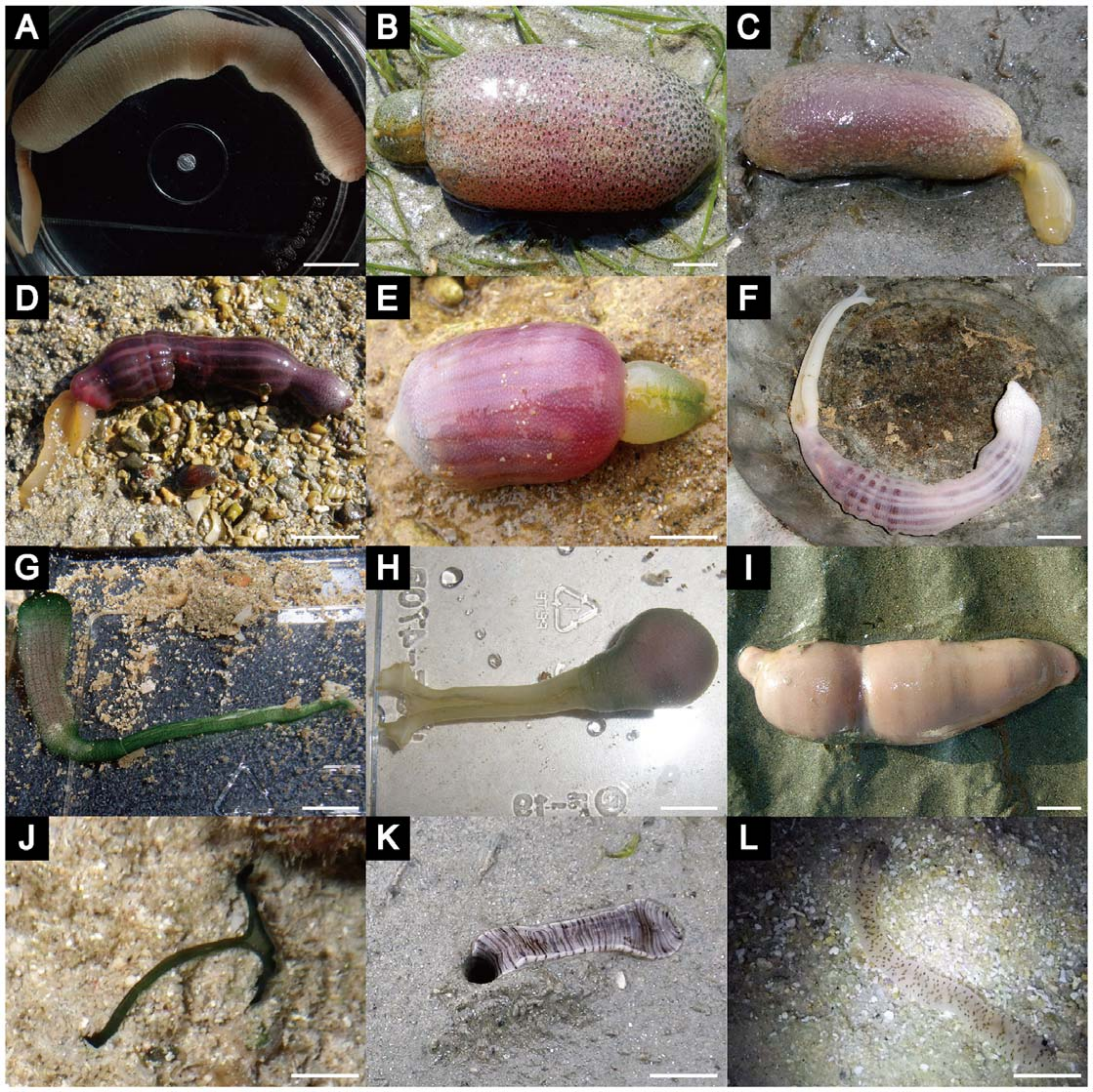
Various echiuran worms that utilized for the analyses. A. *Echiurus echiurus*, B. *Ikedosoma gogoshimense*, C. *Thalassema owstoni*, D. *Listriolobus sorbillans*, E. *Ochetostoma erythrogrammon*, F. *Ochetostoma* sp. 1, G. *Ochetostoma* sp. 2, H. *Ochetostoma* sp. 3, I. *Urechis unicinctus*, J. *Bonellia viridis*, K. *Ikeda taenioides*, L. *Ikeda* sp. 1. Scale bar = 2 cm.

We collected sequence data for the 18S rRNA, 28S rRNA, H3, and COI genes for the molecular phylogenetic analysis of 15 echiuran species belonging to nine genera, five families, and three orders, as well as eight outgroup species (Fig. 1, Table 1, Table S1). The following outgroups were included to root the echiuran tree; five polychaete annelid species representing three families, one sipunculan, and two mollusks (Table 1). The annelid outgroup included three species of Capitellidae (Table 1), which has been suggested as a sister group of Echirua [7], [8]. All sequences were newly obtained in this study except for the sequences of *Arhynchite pugettensis* Fisher, 1949, *Urechis caupo* Fisher & MacGinitie, 1928, and seven outgroup species (Table S1).

**Table 1:** Sampling information for the specimens used in this study.

Order	Species	Family	Sampling locality
Echiuroinea	*Echiurus echiurus* (Pallas, 1767)	Echiuridae	Akkeshi, Hokkaido, Japan
	*Arhynchite pugettensis* Fisher, 1949	Thalassematidae	GenBank
	*Ikedosoma gogoshimense* (Ikeda, 1904)	Thalassematidae	Kure, Hiroshima, Japan
	*Listriolobus sorbillans* (Lampert, 1883)	Thalassematidae	Nago, Okinawa, Japan
	*Ochetostoma erythrogrammon* Leuckart & Ruppell, 1828	Thalassematidae	Kakeroma, Kagoshima, Japan
	*Ochetostoma* sp. 1	Thalassematidae	Kakeroma, Kagoshima, Japan
	*Ochetostoma* sp. 2	Thalassematidae	Uken, Kagoshima, Japan
	*Ochetostoma* sp. 3	Thalassematidae	Kakeroma, Kagoshima, Japan
	*Thalassema owstoni* Ikeda, 1904	Thalassematidae	Kure, Hiroshima, Japan
	*Bonellia viridis* Rolando, 1821	Bonelliidae	Itoman, Okinawa, Japan
Xenopneusta	*Urechis caupo* Fisher & MacGinitie, 1828	Urechidae	GenBank
	*Urechis unicinctus* (von Drasche, 1881)	Urechidae	Iyosaijo, Ehime, Japan
	*Urechis* sp. 1	Urechidae	Kashimanada, Ibaraki, Japan
Heteromyota	*Ikeda taenioides* (Ikeda, 1904)	Ikedidae	Hakatajima, Ehime, Japan
	*Ikeda* sp. 1	Ikedidae	Ishigaki, Okinawa, Japan
Outgroup	*Dasybranchus* sp. 1	Capitellidae	Hakatajima, Ehime, Japan
	*Heteromastus filiformis* (Claparède, 1864)	Capitellidae	GenBank
	*Notomastus tenuis* Moore, 1909	Capitellidae	GenBank
	*Ophelina acuminata* Örsted, 1843	Opheliidae	GenBank
	*Lepidonotus sublevis* Leach, 1816	Polynoidae	GenBank
	*Siphonosoma cumanense* (Keferstein, 1867)	Sipunculidae	GenBank
	*Littorina littorea* (Linnaeus, 1758)	Littorinidae	GenBank
	*Solemya velum* Lamarck, 1818	Solemyidae	GenBank

Taxonomic classification follows Ruppert *et al*. (2004) [3].

**Figure 2:**
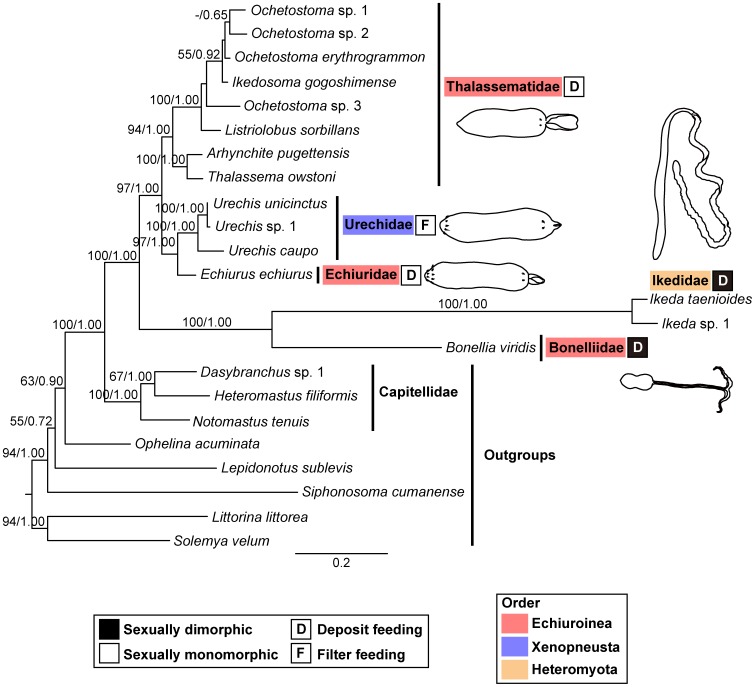
Maximum-likelihood tree of echiuran worms based on the combined dataset of 18S, 28S, H3 and COI genes. Numbers above branches indicate maximum-likelihood bootstrap support values followed by Bayesian posterior probabilities. The colors of the boxes to the right of the family names indicate whether the family is sexually dimorphic (black) or monomorphic (white). The capital letters in the boxes indicate the feeding mode of the family, i.e., deposit feeding (D) or filter feeding (F). The colors shading the family names indicate the order to which the family belongs, as defined by Ruppert *et al*. (2004) [3].

Maximum likelihood (ML) and Bayesian phylogenetic analyses of the combined data set (18S+28S+H3+COI; Dataset S1) suggested that echiurans are monophyletic within the annelids [Maximum-likelihood bootstrap percentage (MBP) = 100, Bayesian posterior probability (BPP) = 1.00] (Fig. 2) and that Capitellidae is sister to echiurans (MBP = 100, BPP = 1.00) (Fig. 2), as in previous molecular studies [7], [8]. Within the Echiura, two major clades were recovered: [Echiuridae+Urechidae+Thalassematidae] (MBP = 97, BPP = 1.00) and [Bonelliidae+Ikedidae] (MBP = 100, BPP = 1.00) (Fig. 2). Within the former major clade, a sister group relationship of Echiuridae and Urechidae was highly supported (MBP = 97, BPP = 1.00) (Fig. 2). Thalassematidae was monophyletic (MBP = 94, BPP = 1.00) and comprised two subclades: [*Ochetostoma*+*Listriolobus*+*Ikedosoma*] (MBP = 100, BPP = 1.00) and [*Thalassema*+*Arhynchite*] (MBP = 100, BPP = 1.00) (Fig. 2). Phylogenetic relationships among the families were consistent between the ML and Bayesian trees (Fig. 2).

Traditionally, echiurans are separated into the three orders: Echiuroinea (with Echiuridae, Thalassematidae, and Bonelliidae), Xenopneusta (with Urechidae) and Heteromyota (with Ikedidae) [3]. However, the phylogenetic relationship among these groups has not been resolved because the informative morphological characters are insufficient to infer it [3]. Our analyses demonstrated that the order-level classification does not actually reflect the evolutionary history of the echiurans, especially in that Echiuroinea is polyphyletic (Fig. 2). This suggests that the taxonomic revision of echiurans is needed, although it requires a further phylogenetic analysis including more echiuran taxa.

A molecular phylogenetic analysis of echiurans based on three genes (16S, 18S, and COI) was recently performed in Lehrke's dissertation work [11]. Her results agree with ours in the following points: (i) Echiura is monophyletic and has a sister group relationship with Capitellidae. (ii) The monophyly of Bonelliidae and Ikedidae is highly supported. (iii) The sister group relationship of Echiuridae and Urechidae is highly supported. A high degree of congruence with her results supports the conclusion of this study. However, there are also some differences between her and our results. For example, in our tree (Fig. 2), the monophyly of Thalassematidae was recovered with strong support (MBP = 94, BPP = 1.00), whereas, in her tree [11], it was ambiguous probably due to the scarcity of the taxa and genes used for the analysis.

### Evolution of marked sexual dimorphism involving dwarf males

Our results suggested that echiurans are separated into two groups; [Thalassematidae+Echiuridae+Urechidae] and [Ikedidae+Bonelliidae] (Fig. 2). These two groups can be distinguished by the presence/absence of marked sexual dimorphism with dwarf males. The members of Echiuridae, Thalassematidae, and Urechidae do not show marked sexual dimorphism, i.e., the males and females are similar in size [1]–[3]. By contrast, those of Bonelliidae and Ikedidae show marked sexual dimorphism, i.e., very tiny dwarf males, the ciliated bodies of which contain only a gonad, a seminal vesicle, and a pair of protonephridia, are parasitic on or within females [1]–[3], [9]. Our analyses suggested that Capitellidae is a sister group of Echiura (Fig. 2), as in previous molecular studies [7], [8]. The most members of this family are sexually monomorphic [12]. Thus, it is highly probable that sexual monomorphism is plesiomorphic for echiurans and that marked sexual dimorphism evolved in the common ancestor of Bonelliidae and Ikedidae (Fig. 2). Among echiurans, the members of the sexually monomorphic group (Echiuridae, Thalassematidae and Urechidae) usually live in littoral and sublittoral waters, whereas those of the sexually dimorphic group (Bonelliidae) are commoner in bathyal and abyssal depth [4], [5]. It is well known that marked sexual dimorphism with dwarf males has evolved in various deep-sea animals (e.g., *Osedax*, anglerfishes, and pedunculate barnacles) [13]–[15]. Perhaps, echiurans may also have evolved dwarf males as an adaptation to the deep-sea environment and some may have secondarily expanded their habitat into shallow waters. To test this hypothesis, a further phylogenetic analysis including deep-sea species is necessary, although they are difficult to collect.

### Evolution of feeding mode

Most echiuran worms are deposit feeders that collect detritus by scooping mud or sand sediments using their flattened and elongated proboscis [1]–[3]. By contrast, members of Urechidae are unusual filter feeders, which use a mucus net to trap food particles [1]–[3]. Our analyses suggested that deposit feeding is a basal feeding mode in echiurans (Fig. 2) and that filter feeding evolved once in the common ancestor of Urechidae (Fig. 2). The argument that deposit feeding is plesiomorphic for echiurans is supported by the fact that all known members of the Capitellidae, the family shown by present analyses and previous molecular studies [7], [8] to be the sister group of echiurans (Fig. 2), are deposit feeders [12].

### Sister group relationship of Bonelliidae and Ikedidae

Our analyses strongly supported a sister group relationship of Bonelliidae and Ikedidae (MBP = 100, BPP = 1.00) (Fig. 2). In addition to sexual dimorphism, several other morphological characters supported this result. Members of Bonelliidae and Ikedidae have unpaired gonoducts, whereas those of Echiuridae, Thalassematidae, and Urechidae have paired gonoducts [3], [16]. Furthermore, Bonelliidae and Ikedidae share certain characters, such as very long proboscises, distally-situated gonostomes and branching anal vesicles [3], [9]. However, there are several morphological differences between these two families. The proboscis is often forked in the former but not the latter [1]–[3] (Fig. 1). In addition, bonelliid females usually have only one or two gonoducts, whereas ikedid females have 10 or more gonoducts [9].

The family Bonelliidae is traditionally grouped into the order Echiuroinea, together with the families Echiuridae and Thalassematidae [3]. However, our molecular analyses suggested that Bonelliidae is separated from the other families of Echiuroinea (Fig. 2). The Bonelliidae has sometimes been grouped into the separate order Bonellioinea primarily based on its marked sexual dimorphism [16]. Our result supported this classification. On the other hand, the analyses included only one species of Bonelliidae, *Bonellia viridis* Rolando, 1821 (Table 1, Fig. 1). Therefore, it will remain unclear whether Ikedidae is a separate family or is included in Bonelliidae until more bonelliids are added to the analyses.

### Sister group relationship of Echiuridae and Urechidae

A sister group relationship between Echiuridae and Urechidae was strongly supported by our analyses (MBP = 97, BPP = 1.00) (Fig. 2). These two families share the character of having anal chaetae rings around the posterior trunk (Fig. 1) [1]–[3], which is absent in the remaining echiurans [1]–[3]. This trait is sometimes considered as a plesiomorphy for echiurans [3]. However, our result strongly suggests that it is an apomorphy for the Echiuridae + Urechidae clade (Fig. 2). On the other hand, these two families are quite different from each other in feeding mode; Echiuridae are deposit feeders as are most other echiurans [17], while Urechidae are filter feeders [1]–[3]. The members of Urechidae have various unique characters (autapomorphies), which are quite distinct from the other echiuran taxa, such as the highly reduced proboscis (Fig. 1), the loss of a vascular system, and the elaboration of the cloaca into water lung [1]–[3]. Probably, the specialization in filter feeding and anal respiration is associated with unique morphological characters of the Urechidae.

### Phylogenetic relationships of genera within Thalassematidae

Approximately eight genera are known from the Thalassematidae [2]. Our molecular analyses included representatives of five thalassematid genera (Table 1). According to the tree (Fig. 2), Thalassematidae was divided into two monophyletic groups: [*Arhynchite* and *Thalassema*] and [*Ochetostoma*, *Listriolobus*, and *Ikedosoma*] (Fig. 2). The longitudinal musculature of the body wall is continuous in the former, while it is thickened into bands in the latter [2]. These morphological characters support the grouping within Thalassematidae in our tree (Fig. 2).

### Conclusion and remaining issues

In this study, we conducted the first molecular phylogenetic analysis of Echiura, using the nuclear and mitochondrial DNA sequence data of representatives of all extant families. The phylogenetic tree supported the monophyly of echiurans and its sister group relationship with Capitellidae. Within the Echiura, two major clades were recovered: [Thalassematidae+Echiuridae+Urechidae] and [Bonelliidae+Ikedidae]. This grouping agreed with the presence/absence of marked sexual dimorphism with dwarf males and the paired/non-paired configuration of the gonoducts. Within the former major clade, the sister group relationship of Echiuridae and Urechidae was highly supported. These two families share the character of having posterior rings of anal chaetae as a synapomorphy. Furthermore, our analyses suggested that deposit feeding is plesiomorphic for echiurans and that filter feeding is apomorphic for Urechidae. Our results did not support the traditional order-level classification, especially in that Echiuroinea was polyphyletic. Our analyses included only shallow-water echiurans. Therefore, deep-sea echiurans should be included in future analyses to completely understand the evolutionary pattern of echiuran worms.

## Materials and Methods

### Sampling and data collection

We collected 13 echiuran specimens from 13 species belonging to five families and three orders, and one specimen of *Dasybranchus* sp. 1 (Capitellidae) as an outgroup species (Table 1, Fig. 1). All specimens were collected in southwestern Japan with the exception of *Echiurus echiurus* (Pallas, 1767) and *Urechis* sp. 1 (Fig. 1), which were collected in northern and eastern Japan, respectively (Table 1). We also included sequence data for *Urechis caupo*, *Arhynchite pugettensis*, and seven outgroup species, which were available in GenBank (Table 1, Table S1).

### Molecular methods

Total DNA was isolated following a previously described method [18]. A small piece of tissue was cut out of the proboscis, homogenized in 800 µl lysis buffer and incubated at 55°C overnight, after which 80 µl saturated potassium chloride was added to the lysate. This solution was incubated for 5 min on ice and then centrifuged for 10 min. The supernatant (700 µl) was transferred to a new tube, cleaned once with a phenol/chloroform solution, and precipitated with an equal volume of 2-propanol. The DNA pellet was rinsed with 70% ethanol, vacuum-dried, and dissolved in 100 µl TE buffer.

We sequenced fragments of the nuclear 18S and 28S ribosomal RNA (rRNA), H3 and COI genes. Polymerase chain reactions (PCRs) were used to amplify ∼1700 bp of 18S rRNA and ∼1000 bp of 28S rRNA, ∼350 bp of H3 and ∼700 bp of COI. Amplifications were performed in 20 µl mixtures consisting of 0.4 µl of forward and reverse primers (20 µm each; primer sequences are provided in Table S2), 2.0 µl of ExTaq buffer, 1.6 µl of dNTPs (2.5 µm each), 0.1 µl of ExTaq polymerase (TaKaRa, Otsu, Japan), and 15.1 µl of distilled water. Thermal cycling was performed with an initial denaturation for 3 min at 94°C, followed by 30 cycles of 30 s at 94°C, 30 s at a gene-specific annealing temperature (Table S2), and 2 min at 72°C, with a final 3 min extension at 72°C. The sequencing reaction was performed using the PCR primers and internal primers (Table S2) and the BigDye Terminator Cycle Sequencing Ready Reaction Kit (Applied Biosystems, Foster City, CA) and electrophoresed on an ABI 3130 sequencer (Applied Biosystems). The obtained sequences have been deposited in the DDBJ/EMBL/GenBank databases with accession numbers AB771455–AB771500 (Table S1). Voucher specimens have been deposited at Kato lab, Graduate school of Human and Environmental Studies, Kyoto University, Japan.

### Phylogenetic analysis

Sequences of the 18S and 28S genes were aligned using the Muscle program [19] with default settings in the software Seaview [20], [21], while those of the H3 and COI genes were aligned without gaps. Obvious misalignments were edited manually using Seaview [20], [21]. The 18S, 28S, H3 and COI alignments contained 522, 369, 273 and 104 variable sites, respectively. Phylogenetic trees were constructed by using the maximum-likelihood (ML) and Bayesian methods. For the ML analysis, model selection and tree search were conducted using the TreeFinder program [22], [23]. The robustness of the ML tree was evaluated by bootstrap analysis with 1000 replications using the same program. Bayesian analyses were performed using MrBayes 3.1.2 [24] with substitution models chosen by Kakusan 4 [25]. In the combined data set, substitution parameters were estimated separately for each gene partition (Table S3). Two independent runs of Metropolis-coupled Markov chain Monte Carlo were carried out simultaneously, sampling trees every 100 generations and calculating the average standard deviation of split frequencies (ASDSF) every 1000 generations. Using the ‘stoprule’ option, analyses were continued until ASDSF dropped below 0.01, at which point the two chains were considered to have achieved convergence. Because ASDSF was calculated based on the last 75% of the samples, we discarded the initial 25% of the sampled trees as burn-in. We confirmed that analyses reached stationarity well before the burn-in period by plotting the ln-likelihood of the sampled trees against generation time.

## Supporting Information

Table S1 **Accession numbers of the specimens used in this study.**
(PDF)

Table S2 **Information on primers and PCR conditions used in this study.**
(PDF)

Table S3 **Information on models of sequence evolution for maximum-likelihood (ML) and Bayesian analyses.**
(PDF)

Dataset S1 **Combined molecular data set.** The data provided includes alignments of the four concatenated molecular data partitions (18S, 28S, H3 and COI).
(PDF)
