# Supplementary material for: Molecular Phylogeny of Echiuran Worms (Phylum: Annelida) Reveals Evolutionary Pattern of Feeding Mode and Sexual Dimorphism
Source: PLoS One. 2013 Feb 14;8(2):e56809. doi: 10.1371/journal.pone.0056809 (PMC3572977; doi:10.1371/journal.pone.0056809)
Supplement: Table S1 — Accession numbers of the specimens used in this study. (PDF) [file pone.0056809.s001.pdf]

Table S1 Accession numbers of the specimens used in this study. Taxonomic classification follows Ruppert *et al.* (2004) [1].

Order	Species	Familiy	18S	28S	H3	COI
Echiuroinea	*Echiurus echiurus*	Echiuridae	AB771455*	AB771469*	-	-
	Arhynchite pugettensis	Thalassematidae	AY210441	AY210455	-	-
	Ikedosoma gogoshimense	Thalassematidae	AB771456*	AB771470*	AB771481*	AB771491*
	Listriolobus sorbillans	Thalassematidae	AB771457*	AB771471*	AB771482*	AB771492*
	Ochetostoma erythrogrammon	Thalassematidae	AB771458*	AB771472*	AB771483*	AB771493*
	Ochetostoma sp. 1	Thalassematidae	AB771459*	AB771473*	AB771484*	-
	Ochetostoma sp. 2	Thalassematidae	AB771460*	-	AB771485*	-
	Ochetostoma sp. 3	Thalassematidae	AB771461*	-	AB771486*	AB771494*
	Thalassema owstoni	Thalassematidae	AB771462*	AB771474*	AB771487*	AB771495*
	Bonellia viridis	Bonelliidae	AB771463*	AB771475*	AB771488*	AB771496*
Xenopneusta	*Urechis caupo*	Urechidae	AF119076	JF509731	JF509712	NC006379
	Urechis unicinctus	Urechidae	AB771464*	AB771476*	-	AB771497*
	Urechis sp. 1	Urechidae	AB771465*	AB771477*	-	AB771498*
Heteromyota	*Ikeda taenioides*	Ikedidae	AB771466*	AB771478*	AB771489*	AB771499*
	Ikeda sp. 1	Ikedidae	AB771467*	AB771479*	AB771490*	AB771500*
Outgroup	*Dasybranchus* sp. 1	Capitellidae	AB771468*	AB771480*	-	-
	Heteromastus filiformis	Capitellidae	DQ790081	DQ790038	-	-
	Notomastus tenuis	Capitellidae	U67323	DQ790028	-	-
	Ophelina acuminata	Opheliidae	DQ790085	DQ790045	HM746761	HQ023899
	Lepidonotus sublevis	Polynoidae	AY894301	DQ790039	-	AY894317
	Siphonosoma cumanense	Sipunculidae	DQ300002	AY445139	DQ300089	AY445139
	Littorina littorea	Littorinidae	DQ093437	FJ977692	DQ093507	DQ093525
	Solemya velum	Solemyidae	AF120524	AY145421	AY070146	U56852

* Sequences obtained for this study are marked with an asterisk.

References

[1] Ruppert EE, Fox RS, Barnes RD (2004) Invertebrate Zoology: A Functional Evolutionary Approach, seventh ed. Belmont: Brooks/Cole-Thompson. 963 p.
