# Supplementary material for: Molecular Phylogeny of Echiuran Worms (Phylum: Annelida) Reveals Evolutionary Pattern of Feeding Mode and Sexual Dimorphism
Source: PLoS One. 2013 Feb 14;8(2):e56809. doi: 10.1371/journal.pone.0056809 (PMC3572977; doi:10.1371/journal.pone.0056809)
Supplement: Table S2 — Information on primers and PCR conditions used in this study. (PDF) [file pone.0056809.s002.pdf]

Table S2 Information on primers and PCR conditions used in this study.

Primer	Direction	Squence 5'–3'	PCR confition
18SrRNA			
PCR amplification and sequencing			94 ℃ 4 min, (94 ℃ 30 s, 55 ℃ 30 s,
G01 [1]	Forward	CACCT GGTTG ATCCT GCCAG	72 ℃ 2 min) x 35, 72 ℃ 5 min
G07 [1]	Reverse	AGCTT GATCC TTCTG CAGGT TCACC TAC	
Sequencing			
G03 [1]	Forward	GTCTG GTGCC AGCAG CCGCG G	
1155F [2]	Forward	CTGAA ACTTA AAGGA ATTGA CGG	
18d [3]	Forward	CACAC CGCCC GTCGC TACTA CCGAT TG	
18Sop [4]	Revese	GCTCC CTCTC CGGAA TCGAA CCC	
G08 [1]	Revese	GAACG GCCAT GCACC ACCAC C	
28SrRNA			
PCR amplification and sequencing			94 ℃ 4 min, (94 ℃ 30 s, 52 ℃ 30 s,
D1 [5]	Forward	ACCCS CTGAA YTTAA GCAT	72 ℃ 2 min) x 40, 72 ℃ 5 min
D3 [6]	Reverse	GACGA TCGAT TTGCA CGTCA	
Sequencing			
D2F [6]	Forward	CCCGT CTTGA AACAC GGACC AAGG	
C2R [7]	Reverse	ACTCT CTCTT CAAAG TTCTT TTC	
H3			
PCR amplification and sequencing			
H3F [8]	Forward	ATGGCTCGTACCAAGCAGACVGC	94 ℃ 4 min, (94 ℃ 30 s, 52 ℃ 30 s,
H3R [8]	Reverse	ATATCCTTRGGCATRATRGTGAC	72 ℃ 2 min) x 40, 72 ℃ 5 min
COI			
PCR amplification and sequencing			
LCO1490 [9]	Forward	GGT CAA CAA TCA TAA AGA TAT TGG	94 ℃ 4 min, (94 ℃ 30 s, 50 ℃ 30 s,
HCO2198 [9]	Reverse	TAA ACT TCA GGG TGA CCA AAA AAT C	72 ℃ 2 min) x 40, 72 ℃ 5 min

References

1. Saunders GW, Kraft GT (1994) Small-subunit rRNA gene sequences from representatives of selected families of the Gigartinales and Rhodymeniales (Rhodophyta). 1. Evidence for the Plocamiales ord. nov. Can J Bot 72: 1250-1263.
2. Wollscheid E, Wägele H (1999) Initial results on the molecular phylogeny of the Nudibranchia (Gastropoda, Opisthobranchia) based on 18S rDNA data. Mol Phylogenet Evol 13: 215-226.
3. Hillis DM, Dixon MT (1991) Ribosomal DNA: Molecular evolution and phylogenetic inference. Q Rev Biol 66: 411-453.
4. Hoso M, Kameda Y, Wu SP, Asami T, Kato M, Hori M (2010) A speciation gene for left-right reversal in snails results in anti-predator adaptation. Nat Comm 1: 133.
5. Colgan DJ, Ponder WF, Beacham E, Macaranas JM (2003) Gastropod phylogeny based on six segments from four genes representing coding or non-coding and mitochondrial or nuclear DNA. Molluscan Res 23: 123-148.
6. Vonnemann V, Schrödl M, Klussmann-Kolb A, Wägele H (2005) Reconstruction of the phylogeny of the Opisthobranchia (Mollusca: Gastropoda) by means of 18S and 28S rRNA gene sequences. J Molluscan Stud 71: 113-125.
7. Dayrat B, Tillier A, Lecointre G, Tillier S (2001) New clades of euthyneuran gastropods (Mollusca) from 28S rRNA sequences. Mol Phylogenet Evol 19: 225-235.
8. Colgan DJ, McLauchlan A, Wilson GDF, Livingston S, Macaranas J, Edgecombe GD, Cassis G, Gray MR (1998) Molecular phylogenetics of the Arthropoda: relationships based on histone H3 and U2 snRNA DNA sequences. Aust J Zool 46: 419-437.
9. Folmer O, Black M, Hoeh W, Lutz RA, Vrijenhoek R (1994) DNA primers for amplification of mitochondrial cytochrome *c* oxidase subunit I from diverse metazoan invertebrates. Mol Mar Biol Biotechnol 3: 294-299.
