# Supplementary material for: Molecular Phylogeny of Echiuran Worms (Phylum: Annelida) Reveals Evolutionary Pattern of Feeding Mode and Sexual Dimorphism
Source: PLoS One. 2013 Feb 14;8(2):e56809. doi: 10.1371/journal.pone.0056809 (PMC3572977; doi:10.1371/journal.pone.0056809)
Supplement: Table S3 — Information on models of sequence evolution for maximum-likelihood (ML) and Bayesian analyses. (PDF) [file pone.0056809.s003.pdf]

**Table S3 Information on models of sequence evolution for maximum-likelihood (ML) and Bayesian analyses.**

| Gene | Substitution model (ML) | Substitution model (Bayesian) |
|---|---|---|
| 18S rRNA | TN+Gamma | SYM+Gamma |
| 28S rRNA | GTR+Gamma | GTR+Gamma |
| H3 | GTR+Gamma | – |
| COI | GTR+Gamma | – |
| H3_1st | – | GTR+Gamma |
| H3_2nd | – | JC69+Homogeneous |
| H3_3rd | – | GTR+Gamma |
| COI_1st | – | HKY85+Gamma |
| COI_2nd | – | SYM+Gamma |
| COI_3rd | – | GTR+Gamma |
