# Supplementary material for: Molecular Phylogeny of Echiuran Worms (Phylum: Annelida) Reveals Evolutionary Pattern of Feeding Mode and Sexual Dimorphism
Source: PLoS One. 2013 Feb 14;8(2):e56809. doi: 10.1371/journal.pone.0056809 (PMC3572977; doi:10.1371/journal.pone.0056809)
Supplement: Dataset S1 — Combined molecular data set. The data provided includes alignments of the four concatenated molecular data partitions (18S, 28S, H3 and COI). (PDF) [file pone.0056809.s004.pdf]

Supporting Information Dataset S1
Combined molecular data set

DATA PARTITIONS

1–2031 18S
2032–3328 28S
3329–3655 H3
3656–4314 COI

```
>Echiurus_echiurus
TAGTCATATG CTTGTCTCAA AGATTAAGCC ATGCATGTCT AAGTACATAC TTTTAC----
--------AC AGTGAAACCG CGAATGGCTC ATTAAATCAG TTAAGGTTCC TTAGAT---C
GTACTATCCT ACTTGGATAA CTGTGGTAAT TCTAGAGCTA ATACATGA-- AAGC-ACGCT
CCGACTGTTG ---------- ---------- --------C- --GGGAAGAG CGTTTTTAT-
TACACCAAAA CCA-ATCGG- ------TCTT C-----GGGC CGTC----AT CTTGGTGACT
CTGAATAACT TTGTGCTGAT CGC-ATGGCC T-C----GAG CCGGCGACGT -ATCTTTCAA
ATGTCTGCCC TATCAAC-TT TCGATGGTAC GTGATATGCC TACCATGGTT GTAACGGGTA
ACGGGGAATC AGGGTTTGAT TCCGGAGAGG GAGCATGAGA AACGGCTACC ACATCCAAGG
AAGGCAGCAG GCGCGCAAAT TACCCACTCC TGGAACGGGG AGGTAGTGAC GAAAAATAAC
AATACGGGAC TCTT-ATGAG GCCTCGTAA- ---------- --TTGGAATG AGTACACTTT
AAATCCTTTA ACGAGGATCT ATTGGAGGGC AAGT-CTGGT GCCAGCAGCC GCGGTAATTC
CAGCTCCAAT AGCGTATATT AAAGTTGTTG CAGTTAAAAA GCTCGTAGTT GGATCTCTGG
T-TTAGGCCG ACGGTTCGC- ---------- ---------C TCG--CGGC- GACTACTGTC
-TG------- ---------- ---------- ---------- ---------- ---TCCTGAC
CT---ACCTG CCGGTT-T-- TCCTCA--GG TGCTCTTGAC TG-AGTGTCT GG------GG
TGGCCGGAAC -GTTTACTTT GAAAAAATTA GAGTGTTCAA AGCAGGCAT- GT--TCGCCT
GAATATTAGT GCATGGAATA ATGGAAGAGG ACCTCGGTTC TATTTT--GT TGGTTTTCGG
AG-CTCGAGG TAATGATTAA GAGGGACTGA CGGGGGCATT CGTATTACGG TGTTAGAGGT
GAAATTCTTG GATCATCGTA AGACGAACTA CTGCGAAAGC ATTTGCCAAG AATGTTTTCA
TTAGTC-AAG AACGAAAGTC GGAGGTTCGA AGACGATCAG ATACCGTCGT AGTTCCGACC
ATAAACGATG CCAACTAGCG ATCCGCCGGC GTTGTTCTCA ---TGACCCG GCGGGCAGCT
TC--CGGGAA ACCAAAGTTT TTGGGTTCCG GGGGAAGTAT GGTTGCAAAG CTGAAACTTA
AAGGAATTGA CGGAAGGGCA CCACCAGGAG TGGAGCCTGC GGCT-TAATT TGACTCAACA
CGGGGAAACT CACCCGGCCC GGACACTGTA AGGATTGACA GATTGAGAGC TCTTTCTTGA
TTCGGTGGGT GGTGGTGCAT GGCCGTTCTT AGTTGGTGGA GCGATTTGTC TGGTTAATTC
CGATAACGAA CGAGACTCTA GCCTATTAAA TAGTTCGCTT GT-------- -TGATGCTGC
AAGTGCA--- ----ACTTCT TAGAGGGACA AGTGTA---- ATATAGACAC ---ACGAGAT
TGAGCAATAA CAGGTCTGTG ATGCCCTTAG ATGTTTGGGG CCGCACGCGC GCTACACTGA
AGGAATCAGC GTGT-----G -TTCTTCCTG CT-CTGCAAA GAGT------ ----------
---------- -----GGGTA ACCCGTTGAA CCTCCTTCGT GCTAGGGATT GGGGCTTGTA
ATTATTCCCC ATGAACGAGG AATTCCCAGT AAGCACGAGT CATCAGCTCG TGTTGATTAC
GTCCCTGCCC TTTGTACACA CCGCCCGTCG CTACTACCGA TTGAACGGTT TAGTGAGATT
CTCGGATCGG ACCCGG--CT TGATGGGTTA CC--GTCGAG CTGTTTGGCG CTGAGAAGAA
ACTCGAACT- TGATC-GTTT AGAGGAAGT- AAAAGTCGTA ACAAGGTTTC CATTACTAAG
CGGAGGAAAA GAAACTAACA A-GGATTCCC CTAGTAACGG CGAGTGAAGC GGGA-ACAGT
CCAGCACCGA ATCCCTCG-C CCTGC----G GC--GAC-GG GAACTGTGGT GTTTGGGACG
A-CCTCTGGG TCGTGCG-TG ACGTGTCC-- ---AGGTTCT CTTGATAGGG A---------
---------C TTTAT----- ---------- -----CCAGA GAGGGTGTCA GGCCCGTGG-
GGCACGTTG- -CGTCGGCT- --CACTGT-- TCGTC-TCTC GAGTCGGGTT G-TTTGGGAA
TGCAGCCCAA ATCTGGTGGT AAACTCCATC TAAGACTAAA TACGGTCGCG AGTCCGATAG
CGGA-CAAGT ACCGTGAGGG AAAGTTGAAA AGAAC-TTTG AAGAGAGAGT TCAAGAGTAC
GTGAAACCGT TCAGAGGTAA ACGGATGGGA CCGCATGCGT C--CCCGCGG ---AATTCAA
CTTGCCTGCG -C--CCGGTG GCGCGG-CGG CTCGGAT-CG C--------- -A--A-GACC
GAACGTCGT- -GTTCAGCC- GGCGCGGGC- ---------- ---------- ---------G
GGCGCATTTT TCGCGGGGAG A-CGCCACGA CCGGTTCTCC GTCGGTCAGG AG-CTC----
------GGCG -GCAAGGTGT ACGG------ --TC--CTTC GGGATCG--- ---GAGC-TT
ATAGGTC-G- --TCGATGT- TTGGCCCGGT G-GAGGACCG AGG------A ATT---GCCC
GC----CG-C CGGTGGGGCC CTTTC----- ------GCTC CGTGCGCTCT ---TTCG---
ACTG-----T GAGGGACTGT TC-T-CAGTG CCTTGCGACT GCGGG----- -CGTATG--T
GGGTGGGGG- ---------- ------TCAA CCCCATCGG- ---------- -----CCTTC
AG---GGTCT GTGGCTGTTC GGTCGGCACC CCATCCGACC CGTCTTGAAA CACGGACCAA
GGAGTCTAAC ATGTGCGCGA GTCAT-GGGA CTATACGAAA TCTA---AAG GCACAATGAA
GGTGA-AGGC CCTC-CGTTG GGTGGGCCTA GGTCTGATCC CT-GCCTCCG --GGCGG-GG
C--------- -GCAAGACTG GCCCGTCTCG TCCCCAT--T GCGGGTGAGG -CGGAGCAAG
AGCGTACACG TTGGGACCCG AAAGATGGTG AACTATGCCT GAGTAGGATG AAGCCAGAGG
AAACTCTGGT GGAGGTCCGT AGCGATTC?? ?????????? ?????????? ??????????
?????????? ?????????? ?????????? ?????????? ?????????? ??????????
?????????? ?????????? ?????????? ?????????? ?????????? ??????????
?????????? ?????????? ?????????? ?????????? ?????????? ??????????
?????????? ?????????? ?????????? ?????????? ?????????? ??????????
?????????? ?????????? ?????????? ?????????? ?????????? ??????????
?????????? ?????????? ?????????? ?????????? ?????????? ??????????
?????????? ?????????? ?????????? ?????????? ?????????? ??????????
?????????? ?????????? ?????????? ?????????? ?????????? ??????????
?????????? ?????????? ?????????? ?????????? ?????????? ??????????
```

```
?????????? ?????????? ?????????? ?????????? ?????????? ??????????
?????????? ?????????? ?????????? ?????????? ?????????? ??????????
?????????? ?????????? ?????????? ?????????? ?????????? ??????????
?????????? ?????????? ?????????? ?????????? ?????????? ??????????
?????????? ?????????? ?????????? ?????????? ?????????? ??????????
?????????? ?????????? ?????????? ?????????? ?????????? ??????????
?????????? ?????????? ?????????? ?????????? ?????????? ????

>Arhynchite_pugettensis
TAGTCATATG CTTGTCTCAA AGATTAAGCC ATGCATGTCT AAGTACATAC TTTTAC----
--------AC AGTGAAACCG CGAATGGCTC ATTAAATCAG TTAAGGTTCC TTAGAT---C
GTACTATCCT ACTTGGATAA CTGTGGTAAT TCTAGAGCTA ATACATGA-- AAGC-ACGCT
CCGACTGTTG ---------- ---------- --------T- --GGGAAGAG CGTTTTTAT-
TACACCAAAA CCA-ATCGG- ------CTTT C-----GGGC CGTC----AA CCTGGTGACT
CTGAATAACT TTGTGCTGAT CGC-ATGGCC T-C----GAG CCGGCGACGT -ATCTTTCAA
ATGTCTGCCC TATCAAC-TT CCGATGGTAC GTGATATGCC TACCATGGTT GTAACGGGTA
ACGGGGAATC AGGGTTTGAT TCCGGAGAGG GAGCATGAGA AACGGCTACC ACATCCAAGG
AAGGCAGCAG GCGCGCAAAT TACCCACTCC TGGAACGGGG AGGTAGTGAC GAAAAATAAC
AATACGGGAC TCTT-ATGAG GCCTCGTAA- ---------- --TTGGAATG AGTACACTTT
AAATCCTTTA ACGAGGATCT ATTGGAGGGC AAGT-CTGGT GCCAGCAGCC GCGGTAATTC
CAGCTCCAAT AGCGTATATT AAAGTTGTTG CAGTTAAAAA GCTCGTAGTT GGATCTCTGG
T-TTAGGCAT GCGGTTCGC- ---------- ---------C TCG--CGGC- GATTACTGTT
-TG------- ---------- ---------- ---------- ---------- ---TCCTGAC
CT---GCCTG CCGGTT-TG- TCCGCT--GA TGCTCTTGAC TG-AGTGTCT GC------GG
TGGCCGGAAC -GTTTACTTT GAAAAAATTA GAGTGTTCAA AGCAGGCCTT TT--CAGCCT
GAATATTAGT GCATGGAATA ATGGAAGAGG ACCTCGGTTC TATTTT--GT TGGTTTTCGG
AG-CTCGAGG TAATGATTAA GAGGGACTGA CGGGGGCATT CGTATTACGG TGTTAGAGGT
GAAATTCTTG GATCATCGTA AGACGAACTA CTGCGAAAGC ATTTGCCAAG GATGTTTTCA
TTAGTC-AAG AACGAAAGTC GGAGGTTCGA AGACGATCAG ATACCGTCGT AGTTCCGACC
ATAAACGATG CCAACTAGCG ATCCGCCGGC GTTGTTCTCA ---TGACCCG GCGGGCAGCT
TC--CGGGAA ACCTAAGTTT TTGGGTTCCG GGGGAAGTAT GGTTGCGAAG CTGAAACTTA
AAGGAATTGA CGGAAGGGCA CCACCAGGAG TGGAGCCTGC GGCT-TAATT TGACTCAACA
CGGGGAAACT CACCCGGCCC GGACACTGTA AGGATTGACA GATTGAGAGC TCTTTCTTGA
TTCGGTGGGT GGTGGTGCAT GGCCGTTCTT AGTTGGTGGA GCGATTTGTC TGGTTAATTC
CGATAACGAA CGAGACTCTA GCCTATTAAA TAGTTCGCCT GT-------- -TCATGCTGC
AGGTGCA--- ----ACTTCT TAGAGGGACA AGTGTA---- ATATAGACAC ---ACGAGAT
TGAGCAATAA CAGGTCTGTG ATGCCCTTAG ATGTTTGGGG CCGCACGCGC GCTACACTGA
AGGAATCAGC GTGT-----G -TTCCTCCTG CT-CTGCAAG GAGC------ ----------
---------- -----GGGTA ACCCGTTGAA CCTCCTTCGT GCTAGGGATT GGGGCTTGTA
ATTATTCCCC ATGAACGAGG AATTCCCAGT AAGCACGAGT CATCAGCTCG TGTTGGTTAC
GTCCCTGCCC TTTGTACACA CCGCCCGTCG CTACTACCGA TTGAATGGTT TAGTGGGATT
CTCGGATCGG ACCCGG--CT TGGTGGGTTA CC--GCCGAG CTGTTTGGCG CTGAGAAGAA
ACTCGAACT- TGATC-ATTT AGAGGAAGT- AAAAGTCGTA ACAAGGTTTC CATTACTAAG
CGGAGGAAAA GAAACTAACA A-GGATTCCC CTAGTAACGG CGAGTGAAGC GGGA-AGAGC
CCAGCACCGA ATCCCCTG-C CCTGT----G GC--AGC-GG GAACTGTGGT GTTTGGGACG
G-CCTCTGGG TCGTGCG-CG GCGAATCC-- ---AAGTTCT CTTGATAGGG A---------
---------C TTTAT----- ---------- -----CCAGA GAGGGTGTCA GGCCCGTGG-
GGATCGCTG- -CGTCGGCT- --CACTGA-- CCGTC-CTTA GAGTCGGGTT G-TTTGGGAA
TGTAGCCCAA ATCTGGTGGT AAACTCCATC TAAGGCTAAA TACGGTCGCG AGTCCGATAG
CGGA-CAAGT ACCGTGAGGG AAAGTTGAAA AGAAC-TTTG AAGAGAGAGT TCAAGAGTAC
GTGAAACCGT TCAGAGGTAA ACGGATGGGA CCGCATGCGT C--CTCGCGG ---AATTCAA
CCTGCCTGTT -G--CTGGAG GCGCT--GTC AGCGGAT-CG C--------- -A--A-GACC
GCGGGCGGT- GCTC-TGCC- TATGCGGGC- ---------- ---------- ---------G
GGCGCATTTT TCGCGGGGAG A-CGCCACGA CCGGTTCTCT GTCGGTCAGG AG-CCT----
------GGCA -GCAAGGTGT ACGA------ --CT--CTTC GGAGTCG--- ---GAGC-TT
ATAGGTT-G- --CCGGAGC- TCGGCCCGAC G-GGGGACCG AGG------A ATT---GCCC
GC----CG-C TGGCGAGGCC C-TTC----- ------GTTC CGTGCGTCCG ---TTCG---
ACTG-----G GAGGGACTGT TC-T-CAGTG CCTTCCGACT GCGGG----- -CGTTCG--T
GGGCGGGGG- ---------- ------TTCC CCTTGTCGG- ---------- -----TTTTT
AG---GGTCA GTGGCTGCTC GGTCGGCACC TCATCCGACC CGTCTTGAAA CACGGACCAA
GGAGTCTAAC ATGTGCGCGA GTCAC-TGGA TTGTACAAAA TCTA---AAG GCGCAATGAA
GGTGA-AGGC CCTC-CGTTC GGTGGGCCTA GGTCTGATCT CT-GCTT-CT --GGCGG-AG
C--------- -GCAAGACTG GCCCGTCTCG TACCCAT--T GTGGTTGGGG -CGGAGCAAG
AGCGTACACG TTGGGACCCG AAAGATGGTG AACTATGCCT GAGTAGGACG AAGTCAGAGG
AAACTCTGAT GGAGGTCCGT AGCGATTC?? ?????????? ?????????? ??????????
?????????? ?????????? ?????????? ?????????? ?????????? ??????????
?????????? ?????????? ?????????? ?????????? ?????????? ??????????
?????????? ?????????? ?????????? ?????????? ?????????? ??????????
?????????? ?????????? ?????????? ?????????? ?????????? ??????????
?????????? ?????????? ?????????? ?????????? ?????????? ??????????
?????????? ?????????? ?????????? ?????????? ?????????? ??????????
?????????? ?????????? ?????????? ?????????? ?????????? ??????????
?????????? ?????????? ?????????? ?????????? ?????????? ??????????
?????????? ?????????? ?????????? ?????????? ?????????? ??????????
?????????? ?????????? ?????????? ?????????? ?????????? ??????????
?????????? ?????????? ?????????? ?????????? ?????????? ??????????
```

?????????? ?????????? ?????????? ?????????? ?????????? ??????????
?????????? ?????????? ?????????? ?????????? ?????????? ??????????
?????????? ?????????? ?????????? ?????????? ?????????? ??????????
?????????? ?????????? ?????????? ?????????? ?????????? ??????????
?????????? ?????????? ?????????? ?????????? ?????????? ????

>Ikedosoma_gogoshimense
TAGTCATATG CTTGTCTCAA AGATTAAGCC ATGCATGTCT AAGTACATAC TTTTAC----
--------AG AGTGAAACCG CGAATGGCTC ATTAAATCAG TTAAGGTTCC TTANAT---C
GTACTATCCT ACTTGGATAA CTGTGGTAAT TCTAGAGCTA ATACATGA-- AAGC-ACGCT
CCGACTGTTC ---------- ---------- ---------- -CGGGAAGAG CGTTTTTAT-
TACACCAAAA CCA-ATCGG- ------TCTC C-----GTGC CGTC----CA CTTGGTGACT
CTGAATAACT TTGTGCTGAT CGCAATGGCC T-C----GAG CCGGCGACGT -ATCTTTCAA
ATGTCTGCCC TATCAAC-TT TCGATGGTAC GTGATATGCC TACCATGGTT GTAACGGGTA
ACGGGGAATC AGGGTTTGAT TCCGGAGAGG GAGCATGAGA AACGGCTACC ACATCCAAGG
AAGGCAGCAG GCGCGCAAAT TACCCACTCC TGGAACGGGG AGGTAGTGAC GAAAAATAAC
AATACGGGAC TCTT-ACGAG GCCTCGTAA- ---------- --TTGGAATG AGTACACTTT
AAATCCTTTA ACGAGGATCT ATTGGAGGGC AAGT-CTGGT GCCAGCAGCC GCGGTAATTC
CAGCTCCAAT AGCGTATATT AAAGTTGTTG CAGTTAAAAA GCTCGTAGTT GGATCTCTGG
TCTCAGGCCG GCGGTTCGC- ---------- ---------C TCG--CGGC- GATTACTGCC
-TG------- ---------- ---------- ---------- ---------- ---TCCTGAC
CT---ACCCG CCGGTT-CG- CCCGCA--GA TGCTCTTGAT TG-AGTGTCT GC------GG
TGGCCGGAAC -GTTTACTTT GAAAAAATTA GAGTGTTCAA AGCAGGCTA- CA--CAGCCT
GAATATTAGT GCATGGAATA ATGGAAGAGG ACCTCGGTTC TATTTT--GT TGGTTTTCGG
AG-CTCGAGG TAATGATTAA GAGGGACTGA CGGGGGCATT CGTATTACGG TGTTAGAGGT
GAAATTCTTG GATCATCGTA AGACGAACTA CTGCGAAAGC ATTTGCCAAG AATGTTTTCA
TTAGTC-AAG AACGAAAGTC GGAGGTTCGA AGACGATCAG ATACCGTCGT AGTTCCGACC
ATAAACGATG CCAACTAGCG ATCCGCCGGC GTTGTTCTCA ---TGACCCG GCGGGCAGCT
TC--CGGGAA ACCAAAGTTT TTGGGTTCCG GGGGAAGTAT GGTTGCAAAG CTGAAACTTA
AAGGAATTGA CGGAAGGGCA CCACCAGGAG TGGAGCCTGC GGCT-TAATT TGACTCAACA
CGGGGAAACT CACCCGGCCC GGACACTGTA AGGATTGACA GATTGAGAGC TCTTTCTTGA
TTCGGTGGGT GGTGGTGCAT GGCCGTTCTT AGTTGGTGGA GCGATTTGTC TGGTTAATTC
CGATAACGAA CGAGACTCTA GCCTATTAAA TAGTTCGCCT GT-------- -TCATGCTGC
AGGCGCA--- ----ACTTCT TAGAGGGACA AGTGTA---- ATATAGACAC ---ACGAGAT
TGAGCAATAA CAGGTCTGTG ATGCCCTTAG ATGTTTGGGG CCGCACGCGC GCTACACTGA
AGGAATCAGC GTGT-----G -TTCTCCCTG CT-CTGCAAG GAGT------ ----------
---------- -----GGGTA ACCCGTTGAA CCTCCTTCGT GCTAGGGATT GGGGCTTGTA
ATTATTCCCC ATGAACGAGG AATTCCCAGT AAGCACGAGT CATCAGCTCG TGTTGATTAC
GTCCCTGCCC TTTGTACACA CCGCCCGTCG CTACTACCGA CTGAATGGTT TAGTGAAATC
CTCGGATCGG ACCCGG--CT TGGTGGGTTA CC--GCCGAG CTGTTTGGCG CTGAGAAGAT
GATTGAACT- TGATC-ACTT GGAGGAAGT- AAAAGTCGTA ACAAGGTTTC CATTACTAAG
CGGAGGAAAA GAAACTAACA A-GGATTCCC CTAGTAACGG CGAGTGAAGC GGGA-AGAGC
CCAGCACCGA ATCCCCTG-C CCTGA----G GC--AGC-CG GAACTGTGGT GTTTGGGACG
G-CCTCTGGG TCGTGTG-CG GCGAGTCC-- ---AGGTTCT CTTGATAGGG A---------
---------C TTCAT----- ---------- -----CCAGA GAGGGTGTCA GGCCCGTGG-
GGCTCGCTT- -CGCCGGCT- --CACTGA-- CCGTC-CTTA GAGTCGGGTT G-TTTGGGAA
TGCAGCCCAA ATCGGGTGGT AAACTCCATC TAAGGCTAAA TACGGTCGCG AGTCCGATAG
CGGA-CAAGT ACCGTGAGGG AAAGTTGAAA AGAAC-TTTG AAGAGAGAGT TCAAGAGTAC
GTGAAACCGT TCAGAGGTAA ACGGATGGGA CCGCATGCGT C--CTCGCGG ---AATTCAA
CCTGCCTGCC -G--AAGGCG GCGCTCGGT- GTCCGAT-CG C--------- -A--A-GACG
GCGCCGGGC- GTCT-GGCC- CAAGCGGGC- ---------- ---------- ---------G
GGCGCATTTT TCGCGGGGAG A-CGCCACGA CCGCTTCCCC GCCGGTCAGG AG-CTC----
------AGCT -GCAAGGTGT ACGA------ --GC--CCTC GGGTTCG--- ---GAGC-TT
ACAGGCT-G- --CTGTTGC- TCGGCCCGGT G-GAGGAGCG AGG------A A-T---GCCC
GC----CG-C TCGCGAGGCC C-TTT----- ------GCTC CGTGCGTCCG ---TTCG---
ACTG-----G GAGGGACTGT CC-T-CAGTG CCTTCCGACC GCGGG----- -CGTACG--G
CGGTGAGGG- ---------- ------TCCC CCTCGCGGG- ---------- -----CCCTG
AG---GGTCT GTGGCTGCTC GGTCGGCACC TCATCCGACC CGTCTTGAAA CACGGACCAA
GGAGTCTAAC ATGTGCGCGA GTCAC-TGGA CTGTACGAAA TCTA---AAG GCGCAATGAA
GGTGA-AGGC CCTC-CGTTC GGTGGGCCTA GGTCTGATCT CT-GCTT-CT --GGCGG-AG
C--------- -GCAAGACTG GCCCGTCTCG TACCCAT--C GCGGTTGAGG -CGGAGCAAG
AGCGTACACG TTGGGACCCG AAAGATGGTG AACTATGCCT GAGTAGGCTC CAGCCAGAGG
AAACTCTGGT GGAGGTCCGT AGCGATTCCG TAAATCTACC GGAGGCAAGG CCCCCAGGAA
GCAGCTCGCC ACCAAAGCTG CCCGCAAGAG TGCTCCCGCC ACTGGTGGCG TGAAGAAGCC
TCACAGATAC AGGCCAGGCA CAGTCGCCCT CCGTGAAATC CGTCGTTACC AGAAGAGCAC
CGAGCTGCTC ATCCGCAAGC TGCCCTTCCA GAGACTGGTG AGAGAAATCG CCCAGGACTT
CAAGACTGAC CTGCGATTCC AGAGCTCTGC TGTCATGGCT CTCCAGGAGG CCAGTGAGGC
TTACCTCGTC GGTCTCTTTG AGNNNNCCAA CTTGTGCGCC ATCCACGCCA AGCGTCACCC
TTTATTTCAT TTTAGGTATC TGAGGGGGTC TCCTAGGAAC ATCAATAAGT CTCCTGATTC
GAGCTGAACT TGGCCAGCCT GGCTCTCTTT TAGCCAATGA CCAAATCTAT AATACAGTCG
TCACTGCCCA TGCCTTTCTA ATAATCTTTT TCTTAGTCAT ACCCATCTTC ATTGGAGGAT
TTGGAAATTG ACTTGTGCCC CTAATACTCT CAGCACCAGA CATAGCATTC CCACGAATAA
ACAACATAAG ATTCTGGTTC CTCCCCCCAG CCTTAATCTT ATTAGTATCC TCTTCTATAA
TCGAGTCGGG GGTGGGAACC GGATGAACCG TATACCCACC GCTCTCAGGC AACCTAGCCC
ACTCTGGCCC TCCAGTAGAT TTAGCAATCT TTTCACTCCA CCTAGCCGGA ATCTCTTCTA
TCCTTGGGGC CATCAACTTT ATCTCCACAG CCTTAAACAT ACGTATTACT GGCTACCTAT

CAGAGAGGAT GCCGCTGTTC GTATGAGCCG TAACCATCAC AGCTATCCTT CTATTGCTAT
CGCTGCCAGT TTTAGCGGGG GCAATTACTA TGCTACTGAC GGACCGAAAC TTAAATACCT
CCTTCTTTGA CCCTGCAGGT GGTGGTGATC CTGTCCTTTA TCAACACCTG TTCT

>Listriolobus_sorbillans
TAGTCATATG CTTGTCTCAA AGATTAAGCC ATGCATGTCT AAGTACATAC TTTTAC----
--------AC AGTGAAACCG CGAATGGCTC ATTAAATCAG TTAAGGTTCC TTAGAT---C
GTACTATCCT ACTTGGATAA CTGTGGTAAT TCTAGAGCTA ATACATGA-- AAGC-ACGCT
CCGACTGTTC ---------- ---------- ---------- -CGGGAAGAG CGTTTTTAT-
TACACCAAAA CCA-ATCGG- ------TCCT C-----GGGC CGTC----CT CTTGGTGACT
CTGAATAACT TTGTGCTGAT CGC-ATGGCC T-C----GAG CCGGCGACGT -ATCTTTCAA
ATGTCTGCCC TATCAAC-TT TCGATGGTAC GTGATATGCC TACCATGGTT GTAACGGGTA
ACGGGGAATC AGGGTTTGAT TCCGGAGAGG GAGCATGAGA AACGGCTACC ACATCCAAGG
AAGGCAGCAG GCGCGCAAAT TACCCACTCC TGGAACGGGG AGGTAGTGAC GAAAAATAAC
AATACGGGAC TCTT-ACGAG GCCTCGTAA- ---------- --TTGGAATG AGTACACTTT
AAATCCTTTA ACGAGGATCT ATTGGAGGGC AAGT-CTGGT GCCAGCAGCC GCGGTAATTC
CAGCTCCAAT AGCGTATATT AAAGTTGTTG CAGTTAAAAA GCTCGTAGTT GGATCTCTGG
TCTCAGGCCG GCGGTTCGC- ---------- ---------C TCG--CGGC- GATTACTGCC
-TG------- ---------- ---------- ---------- ---------- ---TCCTGAC
CT---ACCTG CCGGTT-CG- CCCGCA--GA TGCTCTTGAT TG-AGTGTCT GC------GG
TGGCCGGAAC -GTTTACTTT GAAAAAATTA GAGTGTTCAA AGCAGGCTA- CA--CAGCCT
GAATATTAGT GCATGGAATA ATGGAAGAGG ACCTCGGTTC TATTTT--GT TGGTTTTCGG
AG-CTCGAGG TAATGATTAA GAGGGACTGA CGGGGGCATT CGTATTACGG TGTTAGAGGT
GAAATTCTTG GATCATCGTA AGACGAACTA CTGCGAAAGC ATTTGCCAAG AATGTTTTCA
TTAGTC-AAG AACGAAAGTC GGAGGTTCGA AGACGATCAG ATACCGTCGT AGTTCCGACC
ATAAACGATG CCAACTAGCG ATCCGCCGGC GTTGTTCTCA ---TGACCCG GCGGGCAGCT
TC--CGGGAA ACCAAAGTTT TTGGGTTCCG GGGGAAGTAT GGTTGCAAAG CTGAAACTTA
AAGGAATTGA CGGAAGGGCA CCACCAGGAG TGGAGCCTGC GGCT-TAATT TGACTCAACA
CGGGGAAACT CACCCGGCCC GGACACTGTA AGGATTGACA GATTGAGAGC TCTTTCTTGA
TTCGGTGGGT GGTGGTGCAT GGCCGTTCTT AGTTGGTGGA GCGATTTGTC TGGTTAATTC
CGATAACGAA CGAGACTCTA GCCTATTAAA TAGTTCACCT GT-------- -TCATGCTGC
AGGTGCA--- ----ACTTCT TAGAGGGACA AGTGTA---- ATATAGACAC ---ACGAGAT
TGAGCAATAA CAGGTCTGTG ATGCCCTTAG ATGTTTGGGG CCGCACGCGC GCTACACTGA
AGGAATCAGC GTGT-----G -TTCCCCCTG CT-CTGCAAA GAGT------ ----------
---------- -----GGGTA ACCCGTTGAA CCTCCTTCGT GCTAGGGATT GGGGCTTGTA
ATTATTCCCC ATGAACGAGG AATTCCCAGT AAGCACGAGT CATCAGCTCG TGTTGATTAC
GTCCCTGCCC TTTGTACACA CCGCCCGTCG CTACTACCGA CTGAATGGTT TAGTGAAATC
CTCGGATCGG ACCCGG--CT TGGTGGGTTA CC--GCCGAG CTG-TTGGCG CTGAGAAGAT
GATTGAACT- TGATC-ACTT GGAGGAAGT- AAAAGTCGTA ACAAGGTTTC CATTACTAAG
CGGAGGAAAA GAAACTAACA A-GGATTCCC CTAGTAACGG CGAGTGAAGC GGGA-AGAGC
CCAGCACCGA ATCCCCTG-C CCTGC----G GC--AGC-CG GAACTGTGGT GTTTGGGACG
G-CCTCTGGG TCGTGTG-CG GCGAGTCC-- ---AGGTTCT CTTGATAGGG A---------
---------C TTCAT----- ---------- -----CCAGA GAGGGTGTCA GGCCCGTGG-
GGCTCGCTT- -CGCCGGCT- --CACTGA-- CCGTC-CTTA GAGTCGGGTT G-TTTGGGAA
TGCAGCCCAA ATCGGGTGGT AAACTCCATC TAAGGCTAAA TACGGTCGCG AGTCCGATAG
CGGA-CAAGT ACCGTGAGGG AAAGTTGAAA AGAAC-TTTG AAGAGAGAGT TCAAGAGTAC
GTGAAACCGT TCAGAGGTAA ACGGATGGGA CCGCATGCGT C--CTCGTGG ---AATTCAA
CCTGCCTGCC -G--ACGGCG GCGTCCGGT- GTCCGAT-CG C--------- -A--A-GACG
GCGCCGGGG- GTCTGGGCC- CAAGCGGGC- ---------- ---------- ---------G
GGCGCATTTT TCGCGGGGAG A-CGCCACGA CCGCTTCCCT GCCGGTCAGG AG-CTC----
------GGCT -GCAAGGTGT ACGA------ --GC--CTTC GGGCTCG--- ---GAGC-TT
ATAGGCT-G- --CCGAAGC- TCGGCCCGGC G-GAGGAGCG AGG------A A-T---GCCC
GC----CG-C TCGCGAGGCC C-TTT----- ------GCTC CGTGCGTCTG ---TTCG---
ACTG-----G GAGGGACTGT TC-T-CAGTG CCTTCCGACT GCGGG----- -CGTACG--A
GGGTGGGGG- ---------- ------TCCA CCTCGCGGG- ---------- -----CTTTG
AG---GGTCT GTGGCTGCTC GGTCGGCACC TCATCCGACC CGTCTTGAAA CACGGACCAA
GGAGTCTAAC ATGTGCGCGA GTCAC-TGGA CTGTACGAAA TCTA---AAG GCGCAATGAA
GGTGA-AGGC CCTC-CGTTC GGTGGGCCTA GGTCTGATCT CT-GCCT-CG --GGTGG-AG
C--------- -GCAAGACTG GCCCGTCTCG AACCCAT--C GCGGTTGAGG -CGGAGCAAG
AGCGTACACG TTGGGACCCG AAAGATGGTG AACTATGCCT GAGTAGGACG AAGCCAGAGG
AAACTCTGGT GGAGGTCCGT AGCGATTCCG CAAATCTACC GGAGGCAAGG CCCCCAGGAA
GCAGCTCGCC ACCAAGGCTG CCAGAAAGAG TGCTCCCGCC ACCGGCGGAG TGAAGAAGCC
TCACAGATAC AGGCCAGGCA CAGTCGCCCT CCGTGAGATC CGTCGTTACC AGAAGAGCAC
TGAGCTCCTT ATCCGCAAAC TTCCCTTCCA GAGACTGGTG AGAGAAATCG CCCAGGACTT
CAAGACTGAC CTGCGATTCC AGAGCTCTGC TGTCATGGCT CTCCAGGAGG CCAGTGAGGC
TTACCTCGTC GGTCTTTTTG AGGACACCAA CTTGTGTGCC ATCCACGCCA AGCGTCACCC
TATACTTCAT CTTAGGAGTA TGAGGAGGTC TACTAGGCAC CTCCATAAGC CTAATAATTC
GTGCTGAACT AGGGCAACCT GGCTCCCTGT TAGGTAACGA TCAAATCTAC AATACAGTAG
TAACGGCTCA CGCCTTTTTA ATAATTTTCT TCCTAGTCAT GCCTATTTTT ATTGGAGGAT
TTGGCAACTG ATTAGTTCCC CTAATACTCT CAGCCCCCGA CATGGCGTTT CCTCGTATAA
ATAACATAAG ATTCTGACTT CTTCCCCCTG CCCTCCTTCT ACTAGTCTCC TCTTCAATGA
TTGAGTCTGG AGTAGGGACA GGATGAACTG TCTACCCCCC TTTATCCAGA AATATTGCCC
ACTCTGGGCC CCCAGTGGAC TTAGCCATTT TTTCTTTACA TTTGGCAGGT ATTTCTTCCA
TTCTAGGTGC TATTAACTTT ATCTCAACTG CCATCAACAT GCGAGTGAAA GGGTACCTAG
CGGAAAGAAT GCCTCTGTTC GTTTGAGCTG TCACCATTAC TGCCGTGCTA CTACTTCTAT
CCCTACCCGT GCTAGCCGGT GCCATTACTA TGCTCTTAAC CGACCGTAAC CTAAATACAT

```
CCTTCTTTGA CCCTG----- ---------- ---------- ---------- ----

>Ochetostoma_erythrogrammon
GTGTCATATG CTTGTCTCAA AGATTAAGCC ATGCATGTCT AAGTACATAC TTTTAC----
--------AC AGTGAAACCG CGAATGGCTC ATTAAATCAG TTAAGGTTCC TTAGAT---C
GTACTATCCT ACTTGGATAA CTGTGGTAAT TCTAGAGCTA ATACATGA-- AAGC-ACGCT
CCGACTGTTC ---------- ---------- ---------- -CGGGAAGAG CGTTTTTAT-
TACACCAAAA CCA-ATCGG- ----TCCGTC A-----GGGC CGTC----CA CTTGGTGACT
CTGAATAACT TTGTGCTGAT CGC-ATGGCC T-C----GAG CCGGCGACGT -ATCTTTCAA
ATGTCTGCCC TATCAAC-TT TCGATGGTAC GTGATATGCC TACCATGGTT GTAACGGGTA
ACGGGGAATC AGGGTTTGAT TCCGGAGAGG GAGCATGAGA AACGGCTACC ACATCCAAGG
AAGGCAGCAG GCGCGCAAAT TACCCACTCC TGGAACGGGG AGGTAGTGAC GAAAAATAAC
AATACGGGAC TCTT-ACGAG GCCTCCTAA- ---------- --TTGGAATG AGTACACTTT
AAATCCTTTA ACGAGGATCT ATTGGAGGGC AAGT-CTGGT GCCAGCAACC GCGGTAATTC
CCGCTCCAAT AACGTAAATT AAAGTTGTTG CAGTTAAAAA GCTCGTAGTT GGATCTCTGG
TCTCAGGCCG GCGGTTCGC- ---------- ---------C TCG--CGGC- GATTACTGCC
-TG------- ---------- ---------- ---------- ---------- ---TCCTGAC
CT---ACCCG CCGGTT-CG- CCCGCA--GA TGCTCTTGAT TG-AGTGTCT GT------GG
TGGCCGGAAC -GTTTACTTT GAAAAAATTA GAGTGTTCAA AGCAGGCTT- CA--CAGCCT
GAATATTAGT GCATGGAATA ATGGAAGAGG ACCTCGGTTC TATTTT--GT TGGTTTTCGG
AG-CTCGAGG TAATGATTAA GAGGGACTGA CGGGGGCATT CGTATTACGG TGTTAGAGGT
GAAATTCTTG GATCATCGTA AGACGAACTA CTGCGAAAGC ATTTGCCAAG AATGTTTTCA
TTAGTC-AAG AACGAAAGTC GGAGGTTCGA AGACGATCAG ATACCGTCGT AGTTCCGACC
ATAAACGATG CCAACTAGCG ATCCGCCGGC GTTGTTCTCA ---TGACCCG GCGGGCAGCT
TC--CGGGAA ACCAAAGTTT TTGGGTTCCG GGGGAAGTAT GGTTGCAAAG CTGAAACTTA
AAGGAATTGA CGGAAGGGCA CCACCAGGAG TGGAGCCTGC GGCT-TAATT TGACTCAACA
CGGGGAAACT CACCCGGCCC GGACACTGTA AGGATTGACA GATTGAGAGC TCTTTCTTGA
TTCGGTGGGT GGTGGTGCAT GGCCGTTCTT AGTTGGTGGA GCGATTTGTC TGGTTAATTC
CGATAACGAA CGAGACTCTA GCCTATTAAA TAGTTCGCCT GT-------- -TCATGCTGC
AGGCGCA--- ----ACTTCT TAGAGGGACA AGTGTA---- ATATAGACAC ---ACGAGAT
TGAGCAATAA CAGGTCTGTG ATGCCCTTAG ATGTTTGGGG CCGCACGCGC GCTACACTGA
AGGAATCAGC GTGT-----G -TTCCCCCTG CT-CTGCAAG GAGT------ ----------
---------- -----GGGTA ACCCGTTGAA CCTCCTTCGT GCTAGGGATT GGGGCTTGTA
ATTATTCCCC ATGAACGAGG AATTCCCAGT AAGCACGAGT CATCAGCTCG TGTTGATTAC
GTCCCTGCCC TTTGTACACA CCGCCCGTCG CTACTACCGA CTGAATGGTT TAGTGAAATC
CTCGGATCGG ACCCGG--CT TGGTGGGTTA CC--GCCGAG CTGTTTGGCG CTGAGAAGAA
GATTGAACT- TGATC-AGTT GGAGGAAGT- AAAAGTCGTA ACAAGGTTTC CATTACTAAG
CGGAGGAAAA GAAACTAACA A-GGATTCCC CTAGTAACGG CGAGTGAAGC GGGA-AGAGC
CCAGCACCGA ATCCCCTG-C CCTGC----G GC--AGC-CG GAACTGTGGT GTTTGGGACG
G-CCTCTGGG CCGTGTG-CG GCGAGTCC-- ---AGGTTCT CTTGATAGGG A---------
---------C TTCAT----- ---------- -----CCAGA GAGGGTGTCA GGCCCGTGG-
GGCTCGCTT- -NGCCGGCT- --CACTGA-- CCGTC-CTTA GAGTCGGGTT G-TTTGGGAA
TGCAGCCCAA ATCGGGTGGT AAACTCCATC TAAGGCTAAA TACGGTCGCG AGTCCGATAG
CGGA-CAAGT ACCGTGAGGG AAAGTTGAAA AGAAC-TTTG AAGAGAGAGT TCAAGAGTAC
GTGAAACCGT TCAGAGGTAA ACGGATGGGA CCGCATGCGT C--CTCGCGG ---AATTCAA
CCTGCCTGCC -G--ACGGCG GCGTTCGGT- GCTCGAT-CG C--------- -A--A-GACG
GGCCCGGGC- GTCC-GGCC- CGAGCGGGC- ---------- ---------- ---------G
GGCGCATTTT TCGCGGGGAG A-CGCCACGA CCGCTTCCCC GCCGGTCAGG AG-CTC----
------GGCT -TCAAGGTGT ACGA------ --TC--CTTC GGGGTCG--- ---GAGC-TT
ACAGGTC-G- --CCGCTGC- TCGGCCCGGT G-GAGGAGCG AGG------A A-T---GCCC
GC----CG-C CTGCGAGGCC C-TTC----- ------GCTC CGTGCGTCCG ---TTCG---
ACTG-----G GAGGGACTGT CC-T-CAGTG CCTTCCGACC GCGGG----- -CGTACG--A
GGTCGGGGG- ---------- ------TCCC CCTCGCGGG- ---------- -----CCTTG
AG---GGTCT GTGGCTGCTC GGTCGGCACC TCATCCGACC CGTCTTGAAA CACGGACCAA
GGAGTCTAAC ATGTGCGCGA GTCAT-TGGA CTGTACGAAA TCTA---AAG GCGCAATGAA
GGTGA-AGGC CCAT-CGTTC GGTGGGCCTA GGTCTGATCT CT-GCCTCCC --GGCGG-AG
C--------- -GCAAGACTG GCCCGTNTNN AACCCAT--C GTGGTTGAGG -CGGAGCAAG
AGCGTACACG TTGGGACCCG AAAGATGGTG AACTATGCCT GAGTAGGACG AAGCCAGAGG
AAACTCTGGT GGAGGTCCGT AGCGATTCCG CAAATCTACC GGAGGCAAGG CCCCCAGGAA
GCAGCTCGCC ACCAAAGCTG CCCGCAAAAG TGCTCCCGCC ACTGGCGGTG TGAAGAAGCC
TCACAGATAC AGGCCAGGCA CAGTCGCCCT CCGTGAGATC CGTCGTTACC AGAAGAGTAC
CGAGCTGCTC ATCCGCAAAC TTCCCTTCCA GAGACTGGTG AGAGAAATCG CCCAGGACTT
CAAGACTGAT CTGCGATTCC AGAGCTCCGC TGTCATGGCT CTCCAGGAGG CCAGTGAGGC
TTACCTCGTC GGTCTCTTTG AGGACACCAA CTTGTGTGCC ATCCACGCCA AGCGT-----
---------- ---------- ---------- ---------- ---------- ----------
---------- --------CT GGCTCTCTTT TAGCCAATGA TCAAATCTAT AATACAGTCG
TCACTGCCCA TGCCTTTCTA ATAATCTTTT TCTTAGTCAT ACCCATCTTC ATTGGAGGAT
TTGGAAATTG ACTTGTCCCC CTAATACTCT CAGCACCAGA CATAGCATTC CCACGAATAA
ACAACATAAG ATTCTGGTTC CTCCCCCCAG CCTTAATCTT ATTAGTATCC TCTTCTATAA
TCGAGTCGGG GGTGGGAACC GGATGAACCG TATACCCACC GCTCTCAGGC AACCTAGCCC
ACTCTGGCCC TCCAGTAGAT TTAGCAATTT TTTCACTCCA CCTAGCCGGA ATCTCTTCTA
TTCTTGGGGC CATTAACTTT ATCTCCACAG CCTTAAACAT ACGTATTACT GGCTACCTAT
CAAAGAGGAT GCCACTGTTC GTATGGGCCG TAACCATCAC AGCTATCCTT CTATTACTAT
CGCTGCCAGT TTTAGCGGGG GCAATTACTA TGCTACTGAC GGACCGAAAC TTAAATACCT
CCTTCTTTGA CCCTGCAGGT GGTGGTGATC CTGTCCTTTA ---------- ----
```

>Ochetostoma_sp1
GTGTCATATG CTTGTCTCAA AGATTAAGCC ATGCATGTCT AAGTACATAC TTTTGC----
--------AC AGTGAAACCG CGAATGGCTC ATTAAATCAG TTAAGGTTCC TTAGAT---C
GTACTATCCT ACTTGGATAA CTGTGGTAAT TCTAGAGCTA ATACATGA-- AAGC-ACGCT
CCGACTGTTG ---------- ---------- ---------- -CGGGAAGAG CGTTTTTAT-
TACACCAAAA CCA-ATCGG- ------TCTT C-----GGGC CGTC----CG CTTGGTGACT
CTGAATAACT TTGTGCTGAT CGC-ATGGCC T-C----GAG CCGGCGACGT -ATCTTTCAA
ATGTCTGCCC TATCAAC-TT TCGATGGTAC GTGATATGCC TACCATGGTT GTAACGGGTA
ACGGGGAATC AGGGTTTGAT TCCGGAGAGG GAGCATGAGA AACGGCTACC ACATCCAAGG
AAGGCAGCAG GCGCGCAAAT TACCCACTCC TGGAACGGGG AGGTAGTGAC GAAAAATAAC
AATACGGGAC TCTT-ACGAG GCCTCGTAA- ---------- --TTGGAATG AGTACACTTT
AAATCCTTTA ACGAGGATCT ATTGGAGGGC AAGT-CTGGT GCCAGCAGCC GCGGTAATTC
CAGCTCCAAT AGCGTATATT AAAGTTGTTG CAGTTAAAAA GCTCGTAGTT GGATCTCTGG
TCTCAGGCCG GCGGTTCGC- ---------- ---------C TCG--CGGC- GATTACTGCC
-TG------- ---------- ---------- ---------- ---------- ---TCCTGAC
CT---ACCCG CCGGTT-CG- CCCGCA--GA TGCTCTTGAT TG-AGTGTCT GC------GG
TGGCCGGAAC -GTTTACTTT GAAAAAATTA GAGTGTTCAA AGCAGGCTT- CA--CAGCCT
GAATATTAGT GCATGGAATA ATGGAAGAGG ACCTCGGTTC TATTTT--GT TGGTTTTCGG
AG-CTCGAGG TAATGATTAA GAGGGACTGA CGGGGGCATT CGTATTACGG TGTTAGAGGT
GAAATTCTTG GATCATCGTA AGACGAACTA CTGCGAAAGC ATTTGCCAAG AATGTTTTCA
TTAGTC-AAG AACGAAAGTC GGAGGTTCGA AGACGATCAG ATACCGTCGT AGTTCCGACC
ATAAACGATG CCAACTAGCG ATCCGCCGGC GTTGTTCTCA ---TGACCCG GCGGGCAGCT
TC--CGGGAA ACCAAAGTTT TTGGGTTCCG GGGGAAGTAT GGTTGCAAAG CTGAAACTTA
AAGGAATTGA CGGAAGGGCA CCACCAGGAG TGGAGCCTGC GGCT-TAATT TGACTCAACA
CGGGGAAACT CACCCGGCCC GGACACTGTA AGGATTGACA GATTGAGAGC TCTTTCTTGA
TTCGGTGGGT GGTGGTGCAT GGCCGTTCTT AGTTGGTGGA GCGATTTGTC TGGTTAATTC
CGATAACGAA CGAGACTCTA GCCTATTAAA TAGTTCGCCT GT-------- -TCATGCTGC
AGGCGCA--- ----ACTTCT TAGAGGGACA AGTGTA---- ATATAGACAC ---ACGAGAT
TGAGCAATAA CAGGTCTGTG ATGCCCTTAG ATGTTTGGGG CCGCACGCGC GCTACACTGA
AGGAATCAGC GTGT-----G -TTCTCCCTG CT-CTGCAAG GAGT------ ----------
---------- -----GGGTA ACCCGTTGAA CCTCCTTCGT GCTAGGGATT GGGGCTTGTA
ATTATTCCCC ATGAACGAGG AATTCCCAGT AAGCACGAGT CATCAGCTCG TGTTGATTAC
GTCCCTGCCC TTTGTACACA CCGCCCGTCG CTACTACCGA CTGAATGGTT TAGTGAAATC
CTCGGATCGG ACCCGG--CT TGGTGGGTTA CC--GCCGAG CTGTTTGGCG CTGAGAAGAA
GATTGAACT- TGATC-AGTT GGAGGAAGT- AAAAGTCGTA ACAAGGTTTC CATTACTAAG
CGGAGGAAAA GAAACTAACG A-GGATTCCC CTAGTAACGG CGAGTGAAGC GGGA-AGAGC
CCAGCACCGA ATCCCCTGCC CCTGC----G GC--AGC-CG GAACTGTGGT GTTCGGGACG
G-CCTCTGGG TCGCGCG-GG GCGAGTCC-- ---AGGTTCT CTTGATAGGG A---------
---------C TTCGT----- ---------- -----CCAGA GAGGGTGTCA GGCCCGTGG-
GGCCCGCTC- -GGCCGGCT- --CACTGA-- CCGTC-CCCA GAGTCGGGTT G-TTTGGGAA
TGCAGCCCAA ATCGGGTGGT AAACTCCATC CAAGGCTAAA TACGGTCGCG AGTCCGATAG
CGGA-CAAGT ACCGTGAGGG AAAGTTGAAA AGAAC-TTTG AAGAGAGAGT TCAAGAGTAC
GTGAAACCGT TCAGAGGTAA ACGGATGGGA CCGCACGCGT C--CCCGCGG ---AATTCAA
CCTGCCTGCC -T--AGGGCG GCGTCCATCG GTCCGAT-CT CCCTTAGGTG GG--G-GAGG
GGCCCGGGC- GTC--GGCC- CGAGCGGGC- ---------- ---------- ---------G
GGCGCATTTT TCGCGGGGAG A-CGCCACGA CCGCTTCCCT GTCGGTCAGG AG-CCC----
------GGCT -TCAAGGTGT ACGA------ --CC--CTTC GGGGACG--- ---GAGC-TT
ACAGGCT-G- --CCGGCGC- TCGGCCCGGC G-GGGGAGCG AGG------A A-C---GCCC
GC----CG-C CCGCGAGGCC C-TCC----- ------GCTC CGTGCGTCCG T--TTCG---
ACTG-----G GAGGGACTGT GC-T-CAGTG CCTTCCGACC GCGGG----- -CGTACG--A
GGTCGGGGG- ---------- ------ACCC CCTCGCGGG- ---------- -----CCCAG
AG---GGTCC GTGGCTGCTC GGTCGGCACC TCATCCGACC CGTCTTGAAA CACGGACCAA
GGAGTCTAAC ATGTGCGCGA GTCGC-TGGA CTGTACCAAA TCTG---AAG GCGCAATGAA
GGTGA-AGGC CCTC-CGTTC GGTGGGCCTA GGTCTGATCT CT-GCCCTTT GGGGCGG-AG
C--------- -GCAAGACTG GCCCGTCTCG GACCCAT--C GCGGTCGAGG -CGGAGCAAG
AGCGTACACG TTGGGACCCG AAAGATGGTG AACTATGCCT GAGTAGGACG AAGCCAGAGG
AAACTCTGGT GGAGGTCCGT AGCGATTCCG TAAATCTACC GGAGGCAAGG CCCCCAGGAA
GCAGCTCGCC ACCAAAGCTG CCCGCAAAAG TGCTCCCGCC ACTGGCGGTG TGAAGAAGCC
TCACAGATAC AGGCCAGGCA CAGTCGCCCT TCGTGAGATC CGTCGTTACC AGAAGAGCAC
AGAGCTCCTC ATCCGCAAAC TTCCCTTCCA GAGACTGGTG AGAGAAATCG CCCAGGACTT
CAAGACTGAC CTGCGATTCC AGAGCTCTGC TGTCATGGCT CTCCAGGAGG CCAGTGAGGC
TTACCTCGTC GGTCTCTTTG AGGACACCAA CTTGTGTGCC ATCCACGCCA AGCGT?????
?????????? ?????????? ?????????? ?????????? ?????????? ??????????
?????????? ?????????? ?????????? ?????????? ?????????? ??????????
?????????? ?????????? ?????????? ?????????? ?????????? ??????????
?????????? ?????????? ?????????? ?????????? ?????????? ??????????
?????????? ?????????? ?????????? ?????????? ?????????? ??????????
?????????? ?????????? ?????????? ?????????? ?????????? ??????????
?????????? ?????????? ?????????? ?????????? ?????????? ??????????
?????????? ?????????? ?????????? ?????????? ?????????? ??????????
?????????? ?????????? ?????????? ?????????? ?????????? ??????????
?????????? ?????????? ?????????? ?????????? ?????????? ??????????
?????????? ?????????? ?????????? ?????????? ?????????? ????

>Ochetostoma_sp2
GTGTCATATG CTTGTCTCAA AGATTAAGCC ATGCATGTCT AAGTACATAC TTTTAC----

```
--------AC AGTGAAACCG CGAATGGCTC ATTAAATCAG TTAAGGTTCC TTAGAT---C
GTACTATCCT ACTTGGATAA CTGTGGTAAT TCTAGAGCTA ATACATGA-- AAGC-ACGCT
CCGACTGTTC ---------- ---------- ---------- -CGGGAAGAG CGTTTTTAT-
TACACCAAAA CCA-ATCGG- ------CNTC G-----CGGC CGTC----CG CTTGGTGACT
CTGAATAACT TTGTGCTGAT CGC-ATGGCC T-C----GAG CCGGCGACGT -ATCTTTCAA
ATGTCTGCCC TATCAAC-TT TCGATGGTAC GTGATATGCC TACCATGGTT GTAACGGGTA
ACGGGGAATC AGGGTTTGAT TCCGGAGAGG GAGCATGAGA AACGGCTACC ACATCCAAGG
AAGGCAGCAG GCGCGCAAAT TACCCACTCC TGGAACGGGG AGGTAGTGAC GAAAAATAAC
AATACGGGAC TCTT-ACGAG GCCTCGTAA- ---------- --TTGGAATG AGTACACTTT
AAATCCTTTA ACGAGGATCT ATTGGAGGGC AAGT-CTGGT GCCAGCAGCC GCGGTAATTC
CAGCTCCAAT AGCGTATATT AAAGTTGTTG CAGTTAAAAA GCTCGTAGTT GGATCTCTGG
TCTCAGGCCG GCGGTTCGC- ---------- ---------C TCG--CGGC- GACTACTGCC
-TG------- ---------- ---------- ---------- ---------- ---TCCTGAC
CT---ACCTG CCGGTT-CG- CCCGCA--GA TGCTCTTGAT TG-AGTGTCT GT------GG
TGGCCGGGAC -GTTTACTTT GAAAAAATTA GAGTGTTCAA AGCAGGCTA- CA--CAGCCT
GAATATTAGT GCATGGAATA ATGGAAGAGG ACCTCGGTTC TATTTT--GT TGGTTTTCGG
AG-CTCGAGG TAATGATTAA GAGGGACTGA CGGGGGCATT CGTATTACGG TGTTAGAGGT
GAAATTCTTG GATCATCGTA AGACGAACTA CTGCGAAAGC ATTTGCCAAG AATGTTTTCA
TTAGTC-AAG AACGAAAGTC GGAGGTTCGA AGACGATCAG ATACCGTCGT AGTTCCGACC
ATAAACGATG CCAACTAGCG ATCCGCCGGC GTTGTTCTCA ---TGACCCG GCGGGCAGCT
TC--CGGGAA ACCAAAGTTT TTGGGTTCCG GGGGAAGTAT GGTTGCAAAG CTGAAACTTA
AAGGAATTGA CGGAAGGGCA CCACCAGGAG TGGAGCCTGC GGCTGTAATT TGACTCAACA
CGGGGAAACT CACCCGGCCC GGACACTGTA AGGATTGACA GATTGAGAGC TCTTTCTTGA
TTCGGTGGGT GGTGGTGCAT GGCCGTTCTT AGTTGGTGGA GCGATTTGTC TGGTTAATTC
CGATAACGAA CGAGACTCTA GCCTATTAAA TAGTTCGCCT GT-------- -TCATGCTGC
AGGCGCA--- ----ACTTCT TAGAGGGACA AGTGTA---- ATATAGACAC ---ACGAGAT
TGAGCAATAA CAGGTCTGTG ATGCCCTTAG ATGTTTGGGG CCGCACGCGC GCTACACTGA
AGGAATCAGC GTGT-----G -TTCCCCCTG CT-CTGCAAG GAGT------ ----------
---------- -----GGGTA ACCCGTTGAA CCTCCTTCGT GCTAGGGATT GGGGCTTGTA
ATTATTCCCC ATGAACGAGG AATTCCCAGT AAGCACGAGT CATCAGCTCG TGTTGATTAC
GTCCCTGCCC TTTGTACACA CCGCCCGTCG CTACTACCGA TTGAATGGTT TAGTGAAATC
CTCGGATCGG ACCCGG--CT TGGTGGGTTA CC--GCCGAG CTGTTTGNCG CTGAGAAGAC
GATTGAACTG T-ATC-ATTT AGAGGAAGT- AAAAGTCGTA ACAAGGTTTC C?????????
?????????? ?????????? ?????????? ?????????? ?????????? ??????????
?????????? ?????????? ?????????? ?????????? ?????????? ??????????
?????????? ?????????? ?????????? ?????????? ?????????? ??????????
?????????? ?????????? ?????????? ?????????? ?????????? ??????????
?????????? ?????????? ?????????? ?????????? ?????????? ??????????
?????????? ?????????? ?????????? ?????????? ?????????? ??????????
?????????? ?????????? ?????????? ?????????? ?????????? ??????????
?????????? ?????????? ?????????? ?????????? ?????????? ??????????
?????????? ?????????? ?????????? ?????????? ?????????? ??????????
?????????? ?????????? ?????????? ?????????? ?????????? ??????????
?????????? ?????????? ?????????? ?????????? ?????????? ??????????
?????????? ?????????? ?????????? ?????????? ?????????? ??????????
?????????? ?????????? ?????????? ?????????? ?????????? ??????????
?????????? ?????????? ?????????? ?????????? ?????????? ??????????
?????????? ?????????? ?????????? ?????????? ?????????? ??????????
?????????? ?????????? ?????????? ?????????? ?????????? ??????????
?????????? ?????????? ?????????? ?????????? ?????????? ??????????
?????????? ?????????? ?????????? ?????????? ?????????? ??????????
?????????? ?????????? ?????????? ?????????? ?????????? ??????????
?????????? ?????????? ?????????? ?????????? ?????????? ??????????
?????????? ?????????? ?????????? ?????????? ?????????? ??????????
?????????? ?????????? ????????CG TAAATCTACC GGAGGCAAGG CCCCCAGGAA
GCAGCTCGCC ACCAAAGCTG CCCGCAAGAG TGCTCCCGCC ACTGGCGGAG TGAAGAAGCC
TCACAGATAC AGGCCTGGCA CAGTCGCCCT CCGTGAGATC CGTCGTTACC AGAAGAGCAC
CGAGCTCCTC ATCCGCAAGC TTCCCTTCCA GAGATTGGTG AGAGAAATCG CCCAGGACTT
CAAGACTGAC CTGCGATTCC AGAGCTCTGC TGTCATGGCT CTGCAGGAGG CTAGCGAGGC
TTACCTCGTC GGTCTTTTTG AGGACACCAA CTTGTGTGCC ATCCACGCCA AGCGT?????
?????????? ?????????? ?????????? ?????????? ?????????? ??????????
?????????? ?????????? ?????????? ?????????? ?????????? ??????????
?????????? ?????????? ?????????? ?????????? ?????????? ??????????
?????????? ?????????? ?????????? ?????????? ?????????? ??????????
?????????? ?????????? ?????????? ?????????? ?????????? ??????????
?????????? ?????????? ?????????? ?????????? ?????????? ??????????
?????????? ?????????? ?????????? ?????????? ?????????? ??????????
?????????? ?????????? ?????????? ?????????? ?????????? ??????????
?????????? ?????????? ?????????? ?????????? ?????????? ??????????
?????????? ?????????? ?????????? ?????????? ?????????? ??????????
?????????? ?????????? ?????????? ?????????? ?????????? ????

>Ochetostoma_sp3
TAGTCATATG CTTGTCTCAA AGATTAAGCC ATGCATGTCT AAGTACATAC TTTTAC----
--------AC AGTGAAACCG CGAATGGCTC ATTAAATCAG TTAAGGTTCC TTAGAT---C
GTACTATCCT ACTTGGATAA CTGTGGTAAT TCTAGAGCTA ATACATGA-- AAGC-ACGCT
```

```
CCGACTGTTC ---------- ---------- ---------- -CGGGAAGAG CGTTTTTAT-
TACACCAAAA CCA-ATCGG- ------TCTT C-----GGGC CGTC----CT CTTGGTGACT
CTGAATAACT TTGTGCTGAT CGC-ATGGCC T-C----GAG CCGGCGACGT -ATCTTTCAA
ATGTCTGCCC TATCAAC-TT TCGATGGTAC GTGATATGCC TACCATGGTT GTAACGGGTA
ACGGGGAATC AGGGTTTGAT TCCGGAGAGG GAGCATGAGA AACGGCTACC ACATCCAAGG
AAGGCAGCAG GCGCGCAAAT TACCCACTCC TGGAACGGGG AGGTAGTGAC GAAAAATAAC
AATACGGGAC TCTT-ACGAG GCCTCGTAA- ---------- --TTGGAATG AGTACACTTT
AAATCCTTTA ACGAGGATCT ATTGGAGGGC AAGT-CTGGT GCCAGCAGCC GCGGTAATTC
CAGCTCCAAT AGCGTATATT AAAGTTGTTG CAGTTAAAAA GCTCGTAGTT GGATCTCTGG
TCTCAGGCCG GCGGTTCGC- ---------- ---------C TCG--CGGC- GACTACTGCC
-CG------- ---------- ---------- ---------- ---------- ---TCCTGAC
CT---GCCCG CCGGTT-CG- CCCGCA--GA TGCTCTTGAT TG-AGTGTCT GC------GG
TGGCCGGAAC -GTTTACTTT GAAAAAATTA GAGTGTTCAA AGCAGGCTT- CA--CAGCCT
GAATATTAGT GCATGGAATA ATGGAAGAGG ACCTCGGTTC TATTTT--GT TGGTTTTCGG
AG-CTCGAGG TAATGATTAA GAGGGACTGA CGGGGGCATT CGTATTACGG TGTTAGAGGT
GAAATTCTTG GATCATCGTA AGACGAACTA CTGCGAAAGC ATTTGCCAAG AATGTTTTCA
TTAGTC-AAG AACGAAAGTC GGAGGTTCGA AGACGATCAG ATACCGTCGT AGTTCCGACC
ATAAACGATG CCGACTAGCG ATCCGCCGGC GTTGTTCTCA ---TGACCCG GCGGGCAGCT
TC--CGGGAA ACCAAAGTTT TTGGGTTCCG GGGGAAGTAT GGTTGCAAAG CTGAAACTTA
AAGGAATTGA CGGAAGGGCA CCACCAGGAG TGGAGCCTGC GGCT-TAATT TGACTCAACA
CGGGGAAACT CACCCGGCCC GGACACTGTA AGGATTGACA GATTGAGAGC TCTTTCTTGA
TTCGGTGGGT GGTGGTGCAT GGCCGTTCTT AGTTGGTGGA GCGATTTGTC TGGTTAATTC
CGATAACGAA CGAGACTCTA GCCTATTAAA TAGTTCGCCT GT-------- -TCATGCTGC
AGGCGCA--- ----ACTTCT TAGAGGGACA AGTGTA---- ATATAGACAC ---ACGAGAT
TGAGCAATAA CAGGTCTGTG ATGCCCTTAG ATGTTTGGGG CCGCACGCGC GCTACACTGA
AGGAATCAGC GTGT-----G -TTCTCCCTG CT-CTGCGAG GAGT------ ----------
---------- -----GGGTA ACCCGTTGAA CCTCCTTCGT GCTAGGGATT GGGGCTTGTA
ATTATTCCCC ATGAACGAGG AATTCCCAGT AAGCACGAGT CATCAGCTCG TGTTGATTAC
GTCCCTGCCC TTTGTACACA CCGCCCGTCG CTACTACCGA CTGAATGGTT TAGTGAAATC
CTCGGATCGG ACCCGG--CT TGGTGGGTTA CC--GCCGAG CTGTCTGGCG CTGAGAAGAT
GATTGAACT- TGATC-ACTT GGAGGAAGT- AAAAGTCGTA ACAAGGTTTC C?????????
?????????? ?????????? ?????????? ?????????? ?????????? ??????????
?????????? ?????????? ?????????? ?????????? ?????????? ??????????
?????????? ?????????? ?????????? ?????????? ?????????? ??????????
?????????? ?????????? ?????????? ?????????? ?????????? ??????????
?????????? ?????????? ?????????? ?????????? ?????????? ??????????
?????????? ?????????? ?????????? ?????????? ?????????? ??????????
?????????? ?????????? ?????????? ?????????? ?????????? ??????????
?????????? ?????????? ?????????? ?????????? ?????????? ??????????
?????????? ?????????? ?????????? ?????????? ?????????? ??????????
?????????? ?????????? ?????????? ?????????? ?????????? ??????????
?????????? ?????????? ?????????? ?????????? ?????????? ??????????
?????????? ?????????? ?????????? ?????????? ?????????? ??????????
?????????? ?????????? ?????????? ?????????? ?????????? ??????????
?????????? ?????????? ?????????? ?????????? ?????????? ??????????
?????????? ?????????? ?????????? ?????????? ?????????? ??????????
?????????? ?????????? ?????????? ?????????? ?????????? ??????????
?????????? ?????????? ?????????? ?????????? ?????????? ??????????
?????????? ?????????? ?????????? ?????????? ?????????? ??????????
?????????? ?????????? ?????????? ?????????? ?????????? ??????????
?????????? ?????????? ?????????? ?????????? ?????????? ??????????
?????????? ?????????? ?????????? ?????????? ?????????? ??????????
?????????? ?????????? ????????CG TAAATCTACC GGAGGCAAAG CCCCCAGGAA
GCAGCTCGCC ACCAAAGCTG CCCGTAAGAG TGCTCCCGCC ACCGGCGGAG TGAAGAAGCC
TCACAGATAC AGGCCAGGCA CAGTCGCCCT CCGTGAGATC CGTCGTTACC AGAAGAGCAC
CGAGCTGCTC ATCCGCAAAC TCCCCTTCCA GAGACTGGTG AGAGAAATCG CCCAGGACTT
CAAGACTGAC CTGCGATTCC AGAGCTCTGC TGTCATGGCT CTTCAGGAGG CTAGCGAGGC
CTACCTGGTC GGTCTCTTTG AGGACACCAA CTTGTGCGCC ATCCACGCCA AGCGTAACCT
TATATTTCTT ACTTGGAATT TGGGGTGGCC TATTAGGCAC AGCTATAAGA ATAATAATTC
GTGCTGAACT AGGTCAGCCC GGGTCCCTCC TAGGCAATGA CCAAGTATAT AATACTATTG
TTACTGCCCA TGCTTTTTTA ATAATTTTTT TCTTAGTTAT ACCTATTTTT ATCGGGGGGT
TTGGAAATTG GCTCGTCCCC CTTATGCTGT CTGCCCCAGA CATAGCCTTC CCACGTATGA
ACAACATAAG ATTCTGGCTT CTCCCCCCTG CCCTAATCTT ACTAGTCTCC TCTGCAATAA
TTGAGTCTGG GGTAGGGACA GGCTGAACTG TCTACCCCCC ACTTTCTGGG AACCTGGCCC
ATTCAGGTCC CTCTGTAGAC TTAGCCATCT TCTCCTTACA CCTAGCAGGG ATTTCCTCCA
TCCTGGGGGC TATTAACTTT ATCTCTACTG CCATTAATAT GCGATCAACA GGCTACATCT
CAGAAAGGAT GCCTCTCTTT GTTTGAGCAG TTACAATTAC AGCAGTTCTA CTTCTATTAT
CCCTTCCCGT ATTAGCAGGC GCTATTACTA TGCTACTCAC AGACCGAAAT CTGAACACTT
CATTCTTTGA CCCCGCTGGA GGTGGAGACC CCATCCTATA TCAGCACCTC TTCT

>Thalassema_owstoni
TAGTCATATG CTTGTCTCAA AGATTAAGCC ATGCATGTCT AAGTACATAC TTTTAC----
--------AC AGTGAAACCG CGAATGGCTC ATTAAATCAG TTAAGGTTCC TTAGAT---C
GTACTATCCT ACTTGGATAA CTGTGGTAAT TCTAGAGCTA ATACATGA-- AAGC-ACGCT
CCGACTGTTG ---------- ---------- --------T- --GGGAAGAG CGTTTTTAT-
TACACCAAAA CCA-ATCGG- ------CCTC C-----GGGC CGTC----CA CCTGGTGACT
```

```
CTGAATAACT TTGTGCTGAT CGC-ATGGCC T-C----GAG CCGGCGACGT -ATCTTTCAA
ATGTCTGCCC TATCAACTTT TCGATGGTAC GTGATATGCC TACCATGGTT GTAACGGGTA
ACGGGGAATC AGGGTTTGAT TCCGGAGAGG GAGCATGAGA AACGGCTACC ACATCCAAGG
AAGGCAGCAG GCGCGCAAAT TACCCACTCC TGGAACGGGG AGGTAGTGAC GAAAAATAAC
AATACGGGAC TCTT-ATGAG GCCTCGTAA- ---------- --TTGGAATG AGTACACTTT
AAATCCTTTA ACGAGGATCT ATTGGAGGGC AAGT-CTGGT GCCAGCAGCC GCGGTAATTC
CAGCTCCAAT AGCGTATATT AAAGTTGTTG CAGTTAAAAA GCTCGTAGTT GGATCTCTGG
T-TTAGGCAT GCGGTTCGC- ---------- ---------C TCG--CGGC- GATTACTGTT
-TG------- ---------- ---------- ---------- ---------- ---TCCTGAC
CT---ACCTG CCGGTT-TG- TCCACT--GA TGCTCTTGAC TG-AGTGTCT GT------GG
TGGCCGGAAC -GTTTACTTT GAAAAAATTA GAGTGTTCAA AGCAGGCCTT TT--CAGCCT
GAATATTAGT GCATGGAATA ATGGAAGAGG ACCTCGGTTC TATTTT--GT TGGTTTTCGG
AG-CTCGAGG TAATGATTAA GAGGGACTGA CGGGGGCATT CGTATTACGG TGTTAGAGGT
GAAATTCTTG GATCATCGTA AGACGAACTA CTGCGAAAGC ATTTGCCAAG AATGTTTTCA
TTAGTC-AAG AACGAAAGTC GGAGGTTCGA AGACGATCAG ATACCGTCGT AGTTCCGACC
ATAAACGATG CCAACTAGCG ATCCGCCGGC GTTGTTCTCA ---TGACCCG GCGGGCAGCT
TC--CGGGAA ACCAAAGTTT TTGGGTTCCG GGGGAAGTAT GGTTGCAAAG CTGAAACTTA
AAGGAATTGA CGGAAGGGCA CCACCAGGAG TGGAGCCTGC GGCT-TAATT TGACTCAACA
CGGGGAAACT CACCCGGCCC GGACACTGTA AGGATTGACA GATTGAGAGC TCTTTCTTGA
TTCGGTGGGT GGTGGTGCAT GGCCGTTCTT AGTTGGTGGA GCGATTTGTC TGGTTAATTC
CGATAACGAA CGAGACTCTA GCCTATTAAA TAGTTCGCCT GT-------- -TCATGCTGC
AGGTGCA--- ----ACTTCT TAGAGGGACA AGTGTA---- ATATAGACAC ---ACGAGAT
TGAGCAATAA CAGGTCTGTG ATGCCCTTAG ATGTTTGGGG CCGCACGCGC GCTACACTGA
AGGAATCAGC GTGT-----G -TTCTTCCTG CT-CTGCAAG GAGC------ ----------
---------- -----GGGTA ACCCGTTGAA CCTCCTTCGT GCTAGGGATT GGGGCTTGTA
ATTATTCCCC ATGAACGAGG AATTCCCAGT AAGCACGAGT CATCAGCTCG TGTTGATTAC
GTCCCTGCCC TTTGTACACA CCGCCCGTCG CTACTACCGA CTGAATGGTT TAGTGAGATT
CTTGGATCAG ACCCGG--CT TGGTGGGTTA CC--GCCGAG CTGTTAGGCG CAGAGAAGAA
AATCGAACT- TGATC-ATTT GGAGGAAGT- AAAAGTCGTA ACAAGGTTTC CATTACTAAG
CGGAGGAAAA GAAACTAACA A-GGATTCCC CTAGTAACGG CGAGTGAAGC GGGA-AGAGC
CCAGCACCGA ATCCCCTG-C CCTGT----G GC--AGC-GG GAACTGTGGT GTTTGGGACG
G-CCTCTGTG TCGTGCG-CG GCGAATCC-- ---AGGTTCT CTTGATAGGG A---------
---------C TTTAT----- ---------- -----CCAGA GAGGGTGTCA GGCCCGTGG-
GGTTCGCTG- -CGTCGGCT- --CACTGA-- CCGTC-CTTA GAGTCGGGTT G-TTTGGGAA
TGCAGCCCAA ATCTGGTGGT AAACTCCATC TAAGGCTAAA TACGGTCGCG AGTCCGATAG
CGGA-CAAGT ACCGTGAGGG AAAGTTGAAA AGAAC-TTTG AAGAGAGAGT TCAAGAGTAC
GTGAAACCGT TCAGAGGTAA ACGGATGGGA CCGCATGCGT C--CCCGCGG ---AATTCAA
CCTGCTCGCT -G--TTGGAG GTGCT--GCA GGCGGAT-CG C--------- -A--A-GACC
GTCTGCGGT- GTTC-TGCC- TATGTGAGC- ---------- ---------- ---------G
GGCGCATTTT TCGCGGGGAG A-CGCCACGA CCGGTTCCCT GTCGGTCAGG AG-CCT----
------GGCG -GCAAGGTGT ACTT------ --TC--CTTC GGGATTG--- ---GAGC-TT
ATAGGTC-G- --CCGGTGC- TTGGCCCGAC G-GAGGACCG AGG------A ATT---GCCC
GC----CG-C TGGCGAGGCC C-TTT----- ------GCTC CGTGCGTCCG ---TTCG---
ACTG-----G GAGGGACTGT CC-T-CAGTG CCTTCCGACC GCGGG----- -CGTTCG--T
GTGTAGGGG- ---------- ------TTCC CCTCGTCGG- ---------- -----TCTTA
AG---GGTCT GTGGCTGCTC GGTCGGCACC TCATCCGACC CGTCTTGAAA CACGGACCAA
GGAGTCTAAC ATGTGCGCGA GTCAC-TGGA CTGTACGAAA TCTA---AAG GCGCAATGAA
GGTGA-AGGC CCTC-CGTTA GGTGAGCCTA GGTCTGATCT CT-GCTT-CT --GGCGG-AG
C--------- -GCAAGACTG GCCCGTCTCG AACCCAT--C GTGGTTGAGG -CGGAGCAAG
AGCGTACACG TTGGGACCCG AAAGATGGTG AACTATGCCT GAGTAGGACG AAGTCAGAGG
AAACTCTGAT GGAGGTCCGT AGCGATTCCG TAAATCTACC GGAGGCAAGG CCCCCAGGAA
GCAGCTGGCT ACCAAGGCTG CCCGTAAGAG TGCCCCTGCC ACCGGGGGTG TCAAGAAGCC
TCACAGATAC AGGCCTGGCA CAGTCGCCCT CCGTGAGATC CGTCGTTACC AGAAGAGCAC
TGAGCTCCTC ATCCGCAAAC TGCCCTTCCA GAGACTGGTG AGAGAAATCG CCCAGGACTT
CAAGACAGAT CTGCGTTTCC AGAGCTCTGC TGTCATGGCT CTCCAGGAGG CCAGTGAGGC
TTACTTGGTT GGTCTGTTCG AGGACACCAA CTTGTGCGCT ATCCATGCCA AGCGTCACCC
TATATTTTAT TTTTGGTGCC TGAGGAGGCC TTTTAGGCAC CTCAATAAGA CTAATAATTC
GAGCTGAGCT CGGGCAACCT GGATCTCTCT TAGGAAGAGA CCAGCTCTAC AATACTATCG
TTACAGCACA CGCATTTTTA ATAATTTTCT TCTTAGTAAT ACCTGTATTT ATTGGAGGAT
TTGGTAACTG ACTTGTACCT CTAATACTCT CCGCACCAGA CATAGCATTC CCACGAATAA
ATAACATGAG ATTTTGACTT TTACCTCCAT CCTTAATTCT TCTTGTTTCA TCTGCCATTG
TAGGAAGAGG GGTTGGAACT GGTTGAACTG TTTATCCCCC ACTTGCTAGT AATCTAGCTC
ACTCTGGACC ATCAGTAGAC CTTGCAATTT TCTCTCTACA TCTAGCAGGG GTCTCATCAA
TTCTTGGGGC AATTAATTTT ATCACTACAG TAATTAATAT ACGGTCAAAA AGATATCGGT
CCGAACGAAT GCCATTATTC GTCTGAGCCG TTGCTATTAC CGCTTTACTT CTATTATTAT
CCCTGCCTGT ATTAGCAGGA GCCATTACAA TGCTTTTAAC AGACCG---- ----------
---------- ---------- ---------- ---------- ---------- ----

>Bonellia_viridis
TGATCATATG CTTGTCTCAA AGATTAAGCC ATGCATGTCT AAGTACATAC T---------
--GAATGAAA AGTGAAACCG CGAATGGCTC ATTATACCAG ACAAGGTTCC TTGGAT---C
CTACTATCCT ACTCGGATAA CTGTGGAAAT GCTAGAGCTA ATACGTGACG AACC-ATGCT
CCGACTGTCT ---------- ---------- ---CTCGTGG ACGGGACGAG CGTTTTTGT-
CCGATCAAAA CCA-TTCGG- ---------- CAGCGCAAGC TGCCGGAACC TTAGGTGACT
CTGGGCAACG CTTATCTGAT CGC-ATGGCC TTT----GTG CCGGCGACGA -ATCTTTCAA
ATGTCTGCCC CATCAAT-TC ACGATGGTAG TCGACCGGAC TACAATGGTG TTGACGGGTA
```

```
ACGGGGAATC TGGGTTTGAT TCCGGAGAGG GAGCCTGAGA AACGGCTACC ACATCCAAGG
AAGGCAGCAG GCGCGCAAAT TACCCACTCC CGGCACGGGG AGGTAGTGAC GAAAAATATC
AGTGGGGGTC TCTTAACGAG TGCCCGCCA- ---------- --TTGGAATG AGTACAGCTT
AAATCCTTTG ACGAGGATCA ATTGGAGGGC AAGTTCTGGT GCCAGCAGCC GCGGTAATAC
CAGCTCCAAT AGCGTATATC AAAGTTGTTG CAGTCAAAAA GCTCGTAGTT GGATCTGTGA
ACGGAGGCCG GCGGTCCGC- ---------- ---------C CTGGCGGGTG TGTTGCCGTC
-GG------- ---------- ---------- ---------- ---------- ---TCCTCCT
CA---TCTTG TCGGTTTTAC TCCGTC--GG TGCGCTTGAT TG-AGTGCCG GTC-----GG
GGGCCGGAC- -GGTTACTTT GTGCAAATGA GAGTGCTCAA GACAGGCGGT GAATCGACCT
GTATTTTGT- -CATGGAATA AGCGAAAAAG GCTTCGAGTC TATTTTTCGT TGGTTCATAG
AT-CTCGAGG CAATGATTAA AAGGGACTGA CGGAGGCATT CGTACTACGG GGTTAGAGGT
GAAATTCTTG GATCCCTGTA AGACGAACAA AAGCGAAAGC ATTTGCCAAG AATGTTTTCA
TTAATC-AAG AACGAAAGTC GGAGGTTCAA AGACGATTAG ATACCGTCGT AGTTCCGACC
ATAAACGATG TCGACTAGCC ATCTGCCGCC GTAGTTTCCA A--TGACTCG GTGGGCTGCT
CT--CGGGAA ACCAAAGTGT TTGGATTCCG GGGGAAGTAT GGTTGCAAAG CTGAAACTTA
AAGGAATTGA CGGAAGGGCA CCACCAGGAG TGGAGCCTGC GGCT-TAATT TGACTCAACA
CGGGGAATCT CACCCGGCCC GGACACTGTA AGGATTGACA GATTGAGAGC TCTTTCTTGA
TTCGGTGGTT GGTGGTGCAT GGCCGTTCTT AGTTGGTGGA GCGATTTGTC TGGTCAATTC
CGATAACGAA CGAGACTCTA GCCTGCTCAT TAGGCGGTCG GA-CGCCGTT TCCGACCGGC
CCACG----- -----CTTCT TAGAGGGACA AGTGTATGAG ACAAAGACAC ---ACGAAAT
TGAGCAATAA CAGGTCTGTG ATGCCCTTAG ATGTCTGGGG CCGCACGCGC GCTACACTGA
AGGAATCAGC GAGT-----G -TAATTCCCG CT-CCGAAAG GCAC------ ----------
---------- --TGCGGGGA ACCCGTTGAA CCTCCTTCGT GCTGGGGATT GGGGCTTGTA
ATTCTTGCCC ATGAACGAGG AATTCCTAGT AAGCACGAGT CATCAGCTCG TGTTGATTGA
GTCCCTGCCC TTTGTACACA CCGCCCGTCG ATGACACCGA CTAA------ CAGAGTGATG
AACAGGGCGG ACTCGGGCCC TGTCGACTTA CG-----TCG GCGTTGGGAA CGGGAAAGCC
TGGTGATTCA AGATTGTTTC GGAAGGTGTC AAAAGTCGTA ACAAGGTTTC CATTACTAAG
CGGAGGAAAA GAAAATAACG A-TGATTCCC CCAGTAACGG CGAGTGAAGT GGGA-AGAGC
CTAGCACTGA ATCCCTTGCC GATGC----G GC--AAC-GG GAGCTGTAGT GTTTGGGACG
G-CCTCTGTG CGGTGCG-CG GCGATTCG-- ---AAGTTCG CTTGAAGGCG G---------
---------C TTACTGGGCT TCGTTCGAGG CCTTGCCAGA GAGGGTGTCA GACCCGTGT-
GGAACGTCGC GTCCTCGCG- --CTTTGGTC TCGTC-TCCA GAGTCGGGCT G-TTTGGGAA
TGCAGCCCAA ATGGGGTGGT AAACTCCATC CAAGGCTAAA TACGCTCGCG AGTCCGATAG
CGGACCAAGT ACCGCGAGGG AAAGTTGAAA AGAAC-TTTG AAGAGAGAGT TCAAGAGTAC
GTGAAACCGC TGCGAGGTAA ACGGATGGGA TCGCAATTG- ---CTCGCGG TGAGATTCAG
CTTTCGTTCG -G--TCGGCG ACGTCCGGCT GTCGTCTGCT T--------- ------GTCG
GTGCGTTCTC TCGGGGGCGT ATCGCAGGTG GACGCAGTCG GCGGAGGCGG TCGTTTCGAG
GGTGCACTTC TCCCGCGAGA A-CGCCACGA CCGGTTCTCG GCCTGTCGCA AG-GCG----
------ATCG -GCAAGGTGT TCTGGAACTC ACCCTCCTGT GGGGGTGCCC GGAAACTAAC
ATGGCTCGG- --TCGCTGTT TTGACCAGGC CGATGGACCG AGGCTCGCTG ACGGTCGTCG
GA----GGGC CGTGGCGGCT CGCTG----- ------GTCG AGCGCGTTGT C--GTTGTGT
ATCG-----T CGTGGACTGT CC-T-CAGTG CCCGGCGACC GCGGC----- TCGTGCGAGT
GACTGCGGGC CGTGCGGCGC TCGCCTCCCT CCTCGTCGGG GGAGACGGCG GCCGTCCTAT
TGGTTGGTCG GTGGCTGTTT GGCAGACAAC CCATCCGACC CGTCTTGAAA CACGGACCAA
GGAGTCTAAC ATGTGCGCGA GTCAT-TGTA CTCGACTAGA TACA---CAG GCGCAATGAA
GGTGA----- ---------- -----ACGCA GGTCTGAC-- ---------- --GGAGGGAG
TCGTCCTTTC GGCAAGACCG GCCCGTCCCA TATCGCTTGC GTCGATGTGG ATGGAGCATG
AGCGCAAATG TTGGTACCCG AAAGATGGTG AACTATGCCC GAGTAGGACG AAGCCAGAGG
AAACTCTGGT GGAAGTCCGT AGCGATTCCG TAAATCGACA GGAGGCAAGG CCCCCAGAAA
ACAGCTGGCC ACCAAGGCTG CTCGCAAGAG CGCGCCTGCC ACTGGCGGCG TCAAGAAGCC
TCACAGGTAC AGGCCCGGCA CAGTGGCCCT GCGAGAGATC CGTCGCTACC AGAAGAGCAC
CGAGCTCCTC ATCCGCAAGT TGCCCTTCCA GCGCCTCGTC CGCGAGATCG CCCAGGACTT
CAAGACCGAC CTGCGTTTCC AGAGCTCGGC CGTCATGGCT CTGCAGGAAG CCAGCGAGGC
CTACCTGGTC GGCCTCTTCG AGGACACCAA CCTGTGTGCC ATCCACGCCA AGCGACACAC
TCTACTTCAT TCTTGGTGTA TGAGGAGGTC TCCTTGGCAC ATCCATAAGG CTTATAATCC
GAATTGAACT TGGGCAACCA GGCTCCCTTC TAAACGGAGA TCAACTCTAT AACACTATTG
TTACCGCACA TGCATTCCTA ATAATTTTCT TCCTAGTCAT ACCAATATTT ATTGGTGGTT
TCGGAAACTG ACTTCTACCA TTAATACTTG GCGCCCCTGA CATGGCATTC CCCCGACTAA
ATAACATAAG ATTCTGACTT CTTCCCCCCG CACTGATCAT ACTAGTATCC TCCGCACTAA
TTGGGGATGG AGTAGGGACA GGATGAACAG TATACCCTCC TCTCTCTGGA AACCTCGCCC
ACTCTGGCCC ATCCGTAGAC TTTGCTATCT TCTCCCTCCA CCTTGCAGGA GTATCTTCTA
TCCTCGGTGC CTTAAACTTT ATCACAACCG CAATCAACAT ACGATGAAAT GGAATACGAC
CCGAACGACT ATCTCTATTC GTCTGGGCGG TAACCATTAC AGCAGTCTTA CTACTTCTAT
CTCTCCCTGT TCTCGCGGGA GCCATTACCA TACTCCTAAC CGANCGAAAC CTAAATACTT
CCTTCTTCGA CCCGCAAGGA GGA------- ---------- ---------- ----

>Urechis_caupo
---TCATATG CTTGTCTCAA AGATTAAGCC ATGCATGTCT AAGTACATAC TTTTAC----
--------AC AGTGAAACCG CGAATGGCTC ATTAAATCAG TTAAGGTTCC TTAGAT---C
GTACTATCCT ACTTGGATAA CTGTGGTAAT TCTAGAGCTA ATACATGA-- AAGC-ACGCT
CCGACTGTTG ---------- ---------- --------C- --GGGAAGAG CGTTTTTAT-
TACACCAAAA CCA-ATGGG- ------CTCC CGACGGGGGT CGTT----CT CTTGGTGACT
CTGAATAACT TTGTGCTGAT CGC-ATGGCC T-C----GAG CCGGCGACGT -ATCTTTCAA
ATGTCTGCCC TATCAAC-TT TCGATGGTAC GTGATATGCC TACCATGGTT GTAACGGGTA
ACGGGGAATC AGGGTTTGAT TCCGGAGAGG GAGCATGAGA AACGGCTACC ACATCCAAGG
AAGGCAGCAG GCGCGCAAAT TACCCACTCC TGGAACGGGG AGGTAGTGAC GAAAAATAAC
```

```
AATACGGGAC TCTT-ATGAG GCCTCGTAA- ---------- --TTGGAATG AGTACACTTT
AAATCCTTTA ACGAGGATCC ATTGGAGGGC AAGT-CTGGT GCCAGCAGCC GCGGTAATTC
CAGCTCCAAT AGCGTATATT AAAGTTGTTG CAGTTAAAAA GCTCGTAGTT GGATCTCTGG
T-TTAGGCGG GCGGTTCGC- ---------- ---------C TCG--CGGC- GACTACTGCC
-CG------- ---------- ---------- ---------- ---------- ---TCCTGAC
CT---ACCTG CCGGTT-TGA TTCGCA--GG TGCTCTTGAC TG-AGTGTCT GC------GG
TGGCCGGAAC -GTTTACTTT GAAAAAATTA GAGTGTTCAA AGCAGGCG-- GC--ACGCCT
GAATATTGGT GCATGGAATA ATGGAAGAGG ACCTCGGTTC TATTTT--GT TGGTTTTCGG
AG-CTCGAGG TAATGATTAA GAGGGACTGA CGGGGGCATT CGTATTACGG TGTTAGAGGT
GAAATTCTTG GATCATCGTA AGACGAACTA CTGCGAAAGC ATTTGCCAAG AATGTTTTCA
TTAGTC-AAG AACGAAAGTC GGAGGTTCGA AGACGATCAG ATACCGTCGT AGTTCCGACC
ATAAACGATG CCAACTAGCG ATCCGCCGGC GTTGTTCTCA ---TGACCCG GCGGGCAGCT
TC--CGGGAA ACCAAAGTCT TTGGGTTCCG GGGGAAGTAT GGTTGCAAAG CTGAAACTTA
AAGGAATTGA CGGAAGGGCA CCACCAGGAG TGGAGCCTGC GGCT-TAATT TGACTCAACA
CGGG-AAACT CACCCGGCCC GGACACTGTA AGGATTGACA GATTGAGAGC TCTTTCTTGA
TTCGGTGGGT GGTGGTGCAT GGCCGTTCTT AGTTGGTGGA GCGATTTGTC TGGTTAATTC
CGATAACGAA CGAGACTCTA GCCTATTAAA TAGTTCGCCT GT-------- -TGATGCTGC
AGGTGCA--- ----ACTTCT TAGAGGGACA AGTGTA---- ATATAGACAC ---ACGAGAT
TGAGCAATAA CAGGTCTGTG ATGCCCTTAG ATGTTTGGGG CCGCACGCGC GCTACACTGA
AGAAATCAGC GTGT-----G TTTCTTCCTG CT-CTGTAAG GAGT------ ----------
---------- -----GGGTA ACCCGTTGAA CCTTCTTCGT GCTAGGGATT GGGGCTTGTA
ATTGTTCCCC ATGAACGAGG AATTCCCAGT AAGCACGAGT CATCAGCTCG TGTTGATTAC
GTCCCTGCCC TTTGTACACA CCGCCCGTCG CTACTACCGA TTGAATGGTT TAGTGAGATT
CTCGGATCGG ACCCGG--AT TGATGGGTTA CC--GTCGAT CTGTTTGGCG CTGAGAAGAA
ACTCGAACT- TGATC-ATTT AGAGGAAGT- AAAAGTCGTA ACAAGGTTTC C--TACTAAG
CGGAGGAAAA GAAACTAACA A-GGATTCCC CCAGTAACGG CGAGTGAAGT GGGACGGGGC
CCAGCACCGA ATCCCTCG-C CCTGC----G GC--GAC-GG GAACTGTGGT GTTTGGGACG
T-GCTCTGTG TCGTGCG-TG GCCAGTCC-- ---AGGTTCT CTTGATAGGG A---------
---------C TTGAT----- ---------- -----CCAGA GAGGGTGTCA GGCCCGTGG-
GGCTGGTCG- -TGTCGGCG- --CACTGT-- TCGTC-CTTC GAGTCGGGTT G-TTTGGGAA
TGCAGCCCAA ATATGGTGGT AAACTCCATC TAAGGCTAAA TACGGTCGCG AGTCCGATAG
CGGA-CAAGT ACCGTGAGGG AAAGTTGAAA AGAAC-TTTG AAGAGAGAGT TCAAGAGTAC
GTGAAACCGT TGAGAGGTAA ACGGATGGGA CCGCATGCGT C--CCCGTGG ---AATTCAA
CCTGTCGGCG TT--ACGGCG GCGGGGCCGC TCCGGAT-GG T--------- -AACA-CACC
GGGTGCCCT- GTGTCAGTTC GGCGCGGGC- ---------- ---------- ---------G
GGCGCATTTT TCGCGGGGAG A-CGCCACGA CCGGTTCTCC GTCGGTCTCG AG-CCC----
------GGTG -GCAAGGTGT ACGT------ --CC--TTTC GGGGGCG--- ---GAGC-TT
ATAGGCC-G- --CCGACGT- TTGGCCCGAT GTGGGGACCG AGG------A AATGATACCC
GC----CGCC CCTTGGGGCC CTTTC----- ------TCTC CGGGCGTCCG ---TTCG---
ACTG-----A GAGGGACTGT GC-T-CAGTG CCTTTCGACT GCGGG----- -CGTCTGCTC
GGGAGGAGG- ---------- ------TTCA CCCCGGGGG- ---------- -----CCATT
TG---GGTCA GTGGCTGCTC GGTCGGCACC TCATCCGACC CGTCTTGAAA CACGGACCAA
GGAGTCTAAC ATGTGCGCGA GTCAT-GGGA CTTTACGAAA TCTA---AAG GCGCAATGAA
GGTGA-AGGC TTTC-CGTTC GGTGAGCCCA GGTCTGATCC CTCGCCCGAG WAGGCGGAGG
C--------- -GCACGACTG GCCCGTCTCG TCCCCAT--C GCGGGTGAGG -CGGAGCAAG
AGCGTACACG TTGGGACCCG AAAGATGGTG AACTATGCCT GAGTAGGACG AAGCCAGAGG
AAACTCTGGT GGAGGTCCGT AGCGATTCCG TAAATCTACC GGAGGCAAGG CCCCCAGGAA
GCAGCTGGCT ACCAAGGCCG CCCGTAAGAG CGCCCCAGCC ACCGGTGGTG TGAAGAAGCC
CCACAGATAC AGGCCCGGAA CTGTCGCCCT CCGTGAGATC CGTCGTTACC AGAAGAGCAC
CGAGCTCCTC ATCCGCAAAC TGCCCTTCCA GAGGCTGGTG AGAGAAATCG CTCAGGACTT
CAAAACCGAC CTGCGTTTCC AGAGCTCCGC CGTCATGGCC CTTCAGGAGG CTAGCGAGGC
CTACTTGGTC GGTCTCTTCG AGGACACCAA CCTGTGCGCC ATCCACGCCA AGCG-AACTC
TCTACTTTAT TTTAGGAACT TGAGGAGGCC TTTTAGGAAC TTCTATAAGT CTAATAATTC
GAGCAGAACT AGGGCAACCT GGCTCGCTCC TAGGTAGAGA CCAACTCTAT AACACTATTG
TAACAGCTCA CGCATTCCTT ATAATTTTCT TCTTAGTAAT ACCTGTTTTT ATTGGGGGAT
TTGGAAACTG ATTAGTTCCC TTAATACTTG GGGCACCTGA CATAGCATTC CCTCGTCTAA
ATAATATAAG ATTTTGACTT CTCCCTCCAG CACTCACTCT ACTAGTATCC TCTGCCCTAA
TTGGAGGAGG AGTAGGGACA GGCTGAACTG TATATCCCCC CCTAGCAGGA AATATGGCTC
ATTCTGGCCC CTCAGTAGAT TTAGCAATTT TTTCTCTACA CTTGGCCGGT GTATCTTCAA
TTCTAGGAGC ATTAAATTTT ATTACCACAG TAATTAACAT GCGATGAGAC GGACTACGAC
CAGAACGAAT ACCTTTATTC GTATGGGCTG TAGTCATTAC AGCAGTTCTG CTATTACTAT
CTCTACCTGT TCTTGCAGGC GCAATTACTA TACTCCTAAC AGATCGAAAC CTGAACACTG
CATTCTTTGA CCCTCAAGGA GGAGGAGACC CAGTCCTATA TCAACACCTT TTCT

>Urechis_unicinctus
TAGTCATATG CTTGTCTCAA AGATTAAGCC ATGCATGTCT AAGTACATAC TTTTAC----
--------AC AGTGAAACCG CGAATGGCTC ATTAAATCAG TTAAGGTTCC TTAGAT---C
GTACTATCCT ACTTGGATAA CTGTGGTAAT TCTAGAGCTA ATACATGA-- AAGC-ATGCT
CCGACTGT-- ---------- ---------- ---------G ACGGGAAGAG CGTTTTTAT-
TACACCAAAA CCA-ATCGG- ------CCCT C-TCGGGGGT CGTT----CT CTTGGTGACT
CTGAATAACT TTGTGCTGAT CGC-ATGGCC T-C----GAG CCGGCGACGT -ATCTTTCAA
ATGTCTGCCC TATCAAC-TT TCGATGGTAC GTGATATGCC TACCATGGTT GTAACGGGTA
ACGGGGAATC AGGGTTTGAT TCCGGAGAGG GAGCATGAGA AACGGCTACC ACATCCAAGG
AAGGCAGCAG GCGCGCAAAT TACCCACTCC TGGAACGGGG AGGTAGTGAC GAAAAATAAC
AATACGGGAC TCTT-ATGAG GCCTCGTAA- ---------- --TTGGAATG AGTACACTTT
AAATCCTTTA ACGAGGATCT ATTGGAGGGC AAGT-CTGGT GCCAGCAGCC GCGGTAATTC
```

CAGCTCCAAT AGCGTATATT AAAGTTGTTG CAGTTAAAAA GCTCGTAGTT GGATCTCTGG
T-TTAGGCCG GCGGTTCGC- ---------- ---------C TCG--CGGC- GACTACTGCC
-TG------- ---------- ---------- ---------- ---------- ---TCCTGAC
CT---ACCTG CCGGTT-TG- TCCGCA--GG TGCTCTTGAC TG-AGTGTCT GC------GG
TGGCCGGAAC -GTTTACTTT GAAAAAATTA GAGTGTTCAA AGCAGGCGC- CA---CGCCT
GAATATTAGT GCATGGAATA ATGGAAGAGG ACCTCGGTTC TATTTT--GT TGGTTTTCGG
AG-CTCGAGG TAATGATTAA GAGGGACTGA CGGGGGCATT CGTATTACGG TGTTAGAGGT
GAAATTCTTG GATCATCGTA AGACGAACTA CTGCGAAAGC ATTTGCCAAG AATGTTTTCA
TTAGTC-AAG AACGAAAGTC GGAGGTTCGA AGACGATCAG ATACCGTCGT AGTTCCGACC
ATAAACGATG CCAACTAGCG ATCCGCCGGC GTTGTTCTCA ---TGACCCG GCGGGCAGCT
TC--CGGGAA ACCAAAGTTT TTGGGTTCCG GGGGAAGTAT GGTTGCAAAG CTGAAACTTA
AAGGAATTGA CGGAAGGGCA CCACCAGGAG TGGAGCCTGC GGCT-TAATT TGACTCAACA
CGGGGAAACT CACCCGGCCC GGACACTGTA AGGATTGACA GATTGAGAGC TCTTTCTTGA
TTCGGTGGGT GGTGGTGCAT GGCCGTTCTT AGTTGGTGGA GCGATTTGTC TGGTTAATTC
CGATAACGAA CGAGACTCTA GCCTATTAAA TAGTTCGCCT GT-------- -TGATGCTGC
AGGTGCA--- ----ACTTCT TAGAGGGACA AGTGTA---- ATATAGACAC ---ACGAGAT
TGAGCAATAA CAGGTCTGTG ATGCCCTTAG ATGTTTGGGG CCGCACGCGC GCTACACTGA
AGAAATCAGC GTGT-----G -TTCTTCCTG CT-CTGCAAG GAGT------ ----------
---------- -----GGGTA ACCCGTTGAA CCTTCTTCGT GCTAGGGATT GGGGCTTGTA
ATTATTCCCC ATGAACGAGG AATTCCCAGT AAGCACGAGT CATCAGCTCG TGTTGATTAC
GTCCCTGCCC TTTGTACACA CCGCCCGTCG CTACTACCGA TTGAATGGTT TAGTGAGATT
CTCGGATCGG ACCCGG--AT TGATGGGTTA CC--GTCGAT CTGTTTGGCG CTGAGAAGAA
ACTCGAACT- TGATC-ATTT AGAGGAAGT- AAAAGTCGTA ACAAGGTTTC CATTACTAAG
CGGAGGAAAA GAAACTAACA A-GGATTCCC TCAGTAACGG CGAGTGAAGC GGGA-ATAGC
CCAGCACCGA ATCCCTCG-C CCTGC----G GC--GAC-GG GAACTGTGGT GTTTGGGACG
A-CCTCTGTG TCGTGCG-TG GCCAGTCC-- ---AGGTTCT CTTGATAGGG A---------
---------C TTGAT----- ---------- -----CCAGA GAGGGTGTCA GGCCCGTGG-
GGCTGGTCG- -TGTCGGCG- --CACTGT-- TCGTC-TCTA GAGTCGGGTT G-TTTGGGAA
TGCAGCCCAA ATATGGTGGT AAACTCCATC TAAGGCTAAA TACGGTCGCG AGTCCGATAG
CGGA-CAAGT ACCGTGAGGG AAAGTTGAAA AGAAC-TTTG AAGAGAGAGT TCAAGAGTAC
GTGAAACCGT TGAGAGGTAA ACGGATGGGA CCGCATGCGT C--CCCGTGG ---AATTCAA
CCTGCTGGCG TT--CTGGCG GCGGGAGTGG CTCGGAT-CG C--------- -A--A-GACC
GGACGCTTTC GTTTCAGCT- GGCGTGGGC- ---------- ---------- ---------G
GGCGCATTTT TCGCGGGGAG A-CGCCACGA CCGGTTCTCT GTCGGTCTGG AG-CCC----
------GGTG -GCAAGGTGT ACGT------ --CCCTCTTC TGGGGCG--- ---GAGC-TT
ATAGGCC-G- --CCGACGT- TTGGCCCGAT GTGAGGACCG AGG------A ATTGA-GCCC
GC----CG-C CTTAGGGGCC CTTTC----- ------TCTC CGGGCGTCCG ---TTCG---
ACTG-----A GAGGGACTGT TC-T-CAGTG CCTTTCGACT GCGGG----- -CGTCTG--T
GGGAGGAGG- ---------- ------TCAA CCCCTGGGG- ---------- -----CCATC
AG---GGTCT GTGGCTGCTC GGTCGGCACC TCATCCGACC CGTCTTGAAA CACGGACCAA
GGAGTCTAAC ATGTGCGCGA GTCAT-GGGA CTGTACGAAA TCTA---AAG GCGCAATGAA
GGTGA-AGGC TTTC-CGTTC GGTGGGCCTA GGTCTGATCC CTCGTCGTCG GTGGCGGGGG
C--------- -GCAAGACTG GCCCGTCTCG TCCCCAT--C GTGGGTGAGG -CGGAGCAAG
AGCGTACACG TTGGGACCCG AAAGATGGTG AACTATGCCT GAGTAGGACG AAGCCAGAGG
AAACTCTGGT GGAGGTCCGT AGCGATTC?? ?????????? ?????????? ??????????
?????????? ?????????? ?????????? ?????????? ?????????? ??????????
?????????? ?????????? ?????????? ?????????? ?????????? ??????????
?????????? ?????????? ?????????? ?????????? ?????????? ??????????
?????????? ?????????? ?????????? ?????????? ?????????? ??????????
?????????? ?????????? ?????????? ?????????? ?????????? ?????-----
---------- ---------- --------TC TTCTAGGGAC CTCTATAAGC CTGATAATTC
GAGCTGAACT TGGGCAACCA GGATCACTCC TAGGAAGAGA CCAACTCTAT AATACTATTG
TTACAGCTCA CGCATTCCTT ATAATTTTCT TCTTAGTAAT ACCTGTTTTT ATTGGGGGGT
TCGGAAACTG ATTAGTTCCC CTAATACTTG GGGCACCAGA TATAGCATTC CCGCGACTAA
ATAACATAAG ATTCTGACTT CTCCCTCCTG CACTAACACT CCTTGTATCT TCCGCACTAA
TTGGTGGAGG AGTAGGAACA GGGTGAACAG TCTACCCCCC TCTTTCAGGA AATATAGCCC
ACTCCGGCCC ATCAGTAGAC TTAGCAATTT TCTCTCTCCA CCTAGCTGGG GTATCTTCTA
TTCTTGGGGC ACTAAATTTT ATTACTACAG TAATTAACAT ACGATGAGAT GGCCTACGAC
CAGAACGAAT GCCTTTATTT GTATGGGCCG TAGTCATTAC AGCAGTACTA TTACTTTTAT
CCCTCCCAGT TCTAGCAGGG GCAATTACTA TACTTCTGAC AGACCGAAAC CTAAATACCG
CATTTTTCGA CCCCCAAGGG GGTGGTGACC CTGTGTTATA CCAACATCTC TTTT

>Urechis_sp1
TAGTCATATG CTTGTCTCAA AGATTAAGCC ATGCATGTCT AAGTACATAC TTTTAC----
--------AC AGTGAAACCG CGAATGGCTC ATTAAATCAG TTAAGGTTCC TTAGAT---C
GTACTATCCT ACTTGGATAA CTGTGGTAAT TCTAGAGCTA ATACATGA-- AAGC-ATGCT
CCGACTGTGA ---------- ---------- --------C- --GGGAAGAG CGTTTTTAT-
TACACCAAAA CCA-ATCGG- ------CCCT C-TCGGGGGT CGTT----CT CTTGGTGACT
CTGAATAACT TTGTGCTGAT CGC-ATGGCC T-C----GAG CCGGCGACGT -ATCTTTCAA
ATGTCTGCCC TATCAAC-TT TCGATGGTAC GTGATATGCC TACCATGGTT GTAACGGGTA
ACGGGGAATC AGGGTTTGAT TCCGGAGAGG GAGCATGAGA AACGGCTACC ACATCCAAGG
AAGGCAGCAG GCGCGCAAAT TACCCACTCC TGGAACGGGG AGGTAGTGAC GAAAAATAAC
AATACGGGAC TCTT-ATGAG GCCTCGTAA- ---------- --TTGGAATG AGTACACTTT
AAATCCTTTA ACGAGGATCT ATTGGAGGGC AAGT-CTGGT GCCAGCAGCC GCGGTAATTC
CAGCTCCAAT AGCGTATATT AAAGTTGTTG CAGTTAAAAA GCTCGTAGTT GGATCTCTGG
T-TTAGGCCG GCGGTTCGC- ---------- ---------C TCG--CGGC- GACTACTGCC

-TG------- ---------- ---------- ---------- ---------- ---TCCTGAC
CT---ACCTG CCGGTT-TG- TCCGCA--GG TGCTCTTGAC TG-AGTGTCT GC------GG
TGGCCGGAAC -GTTTACTTT GAAAAAATTA GAGTGTTCAA AGCAGGCGC- CA---CGCCT
GAATATTAGT GCATGGAATA ATGGAAGAGG ACCTCGGTTC TATTTT--GT TGGTTTTCGG
AG-CTCGAGG TAATGATTAA GAGGGACTGA CGGGGGCATT CGTATTACGG TGTTAGAGGT
GAAATTCTTG GATCATCGTA AGACGAACTA CTGCGAAAGC ATTTGCCAAG AATGTTTTCA
TTAGTC-AAG AACGAAAGTC GGAGGTTCGA AGACGATCAG ATACCGTCGT AGTTCCGACC
ATAAACGATG CCAACTAGCG ATCCGCCGGC GTTGTTCTCA ---TGACCCG GCGGGCAGCT
TC--CGGGAA ACCAAAGTTT TTGGGTTCCG GGGGAAGTAT GGTTGCAAAG CTGAAACTTA
AAGGAATTGA CGGAAGGGCA CCACCAGGAG TGGAGCCTGC GGCT-TAATT TGACTCAACA
CGGGGAAACT CACCCGGCCC GGACACTGTA AGGATTGACA GATTGAGAGC TCTTTCTTGA
TTCGGTGGGT GGTGGTGCAT GGCCGTTCTT AGTTGGTGGA GCGATTTGTC TGGTTAATTC
CGATAACGAA CGAGACTCTA GCCTATTAAA TAGTTCGCCT GT-------- -TGATGCTGC
AGGTGCA--- ----ACTTCT TAGAGGGACA AGTGTA---- ATATAGACAC ---ACGAGAT
TGAGCAATAA CAGGTCTGTG ATGCCCTTAG ATGTTTGGGG CCGCACGCGC GCTACACTGA
AGAAATCAGC GTGT-----G -TTCTTCCTG CT-CTGCAAG GAGT------ ----------
---------- -----GGGTA ACCCGTTGAA CCTTCTTCGT GCTAGGGATT GGGGCTTGTA
ATTATTCCCC ATGAACGAGG AATTCCCAGT AAGCACGAGT CATCAGCTCG TGTTGATTAC
GTCCCTGCCC TTTGTACACA CCGCCCGTCG CTACTACCGA TTGAATGGTT TAGTGAGATT
CTCGGATCGG ACCCGG--AT TGATGGGTTA CC--GTCGAT CTGTTTGGCG ----------
---------- ---------- ---------- ---------- ---------- -ATTACTAAG
CGGAGGAAAA GAAACTAACA A-GGATTCCC TCAGTAACGG CGAGTGAAGC GGGA-ATAGC
CCAGCACCGA ATCCCTCG-C CCTGC----G GC--GAC-GG GAACTGTGGT GTTTGGGACG
A-CCTCTGTG TCGTGCG-TG GCCAGTCC-- ---AGGTTCT CTTGATAGGG A---------
---------C TTGAT----- ---------- -----CCAGA GAGGGTGTCA GGCCCGTGG-
GGCTGGTCG- -TGTCGGCG- --CACTGT-- TCGTC-TCTA GAGTCGGGTT G-TTTGGGAA
TGCAGCCCAA ATATGGTGGT AAACTCCATC TAAGGCTAAA TACGGTCGCG AGTCCGATAG
CGGA-CAAGT ACCGTGAGGG AAAGTTGAAA AGAAC-TTTG AAGAGAGAGT TCAAGAGTAC
GTGAAACCGT TGAGAGGTAA ACGGATGGGA CCGCATGCGT C--CCCGTGG ---AATTCAA
CCTGCTGGCG TT--CTGGCG GCGGGAGTGG CTCGGAT-CG C--------- -A--A-GACC
GGACGCTTTC GTTTCAGCT- GGCGTGGGC- ---------- ---------- ---------G
GGCGCATTTT TCGCGGGGAG A-CGCCACGA CCGGTTCTCT GTCGGTCTGG AG-CCC----
------GGTG -GCAAGGTGT ACGT------ --CCCTCTTC TGGGGCG--- ---GAGC-TT
ATAGGCC-G- --CCGACGT- TTGGCCCGAT GTGAGGACCG AGG------A ATTGA-GCCC
GC----CG-C CTTAGGGGCC CTTTC----- ------TCTC CGGGCGTCCG ---TTCG---
ACTG-----A GAGGGACTGT TC-T-CAGTG CCTTTCGACT GCGGG----- -CGTCTG--T
GGGAGGAGG- ---------- ------TCAA CCCCTGGGG- ---------- -----CCATC
AG---GGTCT GTGGCTGCTC GGTCGGCACC TCATCCGACC CGTCTTGAAA CACGGACCAA
GGAGTCTAAC ATGTGCGCGA GTCAT-GGGA CTGTACGAAA TCTA---AAG GCGCAATGAA
GGTGA-AGGC TTTC-CGTTC GGTGGGCCTA GGTCTGATCC CTCGTCGTCG GTGGCAGGGG
C--------- -GCAAGACTG GCCCGTCTCG TCCCCAT--C GTGGGTGAGG -CGGAGCAAG
AGCGTACACG TTGGGACCCG AAAGATGGTG AACTATGCCT GAGTAGGACG AAGCCAGAGG
AAACTCTGGT GGAGGTCCGT AGCGATTC?? ?????????? ?????????? ??????????
?????????? ?????????? ?????????? ?????????? ?????????? ??????????
?????????? ?????????? ?????????? ?????????? ?????????? ??????????
?????????? ?????????? ?????????? ?????????? ?????????? ??????????
?????????? ?????????? ?????????? ?????????? ?????????? ??????????
?????????? ?????????? ?????????? ?????????? ?????????? ?????AACTC
TCTACTTTAT CCTCGGAACT TGAGGAGGTC TTCTAGGGAC CTCTATAAGC CTGATAATTC
GGGCTGAACT TGGGCAACCA GGATCACTCC TAGGAAGAGA CCAACTCTAT AATACTATTG
TTACAGCTCA CGCATTCCTT ATAATTTTCT TCTTAGTAAT ACCTGTTTTT ATTGGAGGGT
TCGGAAACTG GTTAGTTCCC CTAATACTTG GGGCACCAGA TATAGCATTT CCGCGGCTAA
ATAATATAAG ATTCTGACTT CTCCCTCCTG CACTAACACT CCTTGTATCT TCCGCACTAA
TTGGCGGAGG AGTAGGAACA GGGTGAACAG TCTACCCCCC TCTTTCAGGA AATATGGCCC
ACTCCGGCCC ATCAGTAGAT TTAGCAATTT TCTCTCTCCA CCTAGCCGGG GTATCTTCTA
TTCTTGGGGC ACTAAATTTT ATTACTACAG TAATTAACAT ACGATGAGAT GGCCTACGAC
CAGAACGAAT ACCTTTATTT GTATGGGCCG TAGTCATTAC AGCAGTACTA TTACTCTTAT
CTCTCCCAGT TCTAGCAGGG GCAATTACTA TACTTCTAAC AGACCGAAAC CTAAATACCG
CATTTTTCGA CCCTCAAGGT GGTGGTGACC CTGTATTATA CCAACATCTC TTTT

>Ikeda_taenioides
TAGTTATATG CTTATCTCAA AGATTAAGCC ATGCAGGTGC AAGTACCCGC TGCTTCTCGC
AAGAGACAGC AGCTAAACCG CGGATGGCTC AGTAAATCAG ACAGAGTTCA CTAGATGAAC
GCACCAG--T AGTCGGATAA CTGTGGAAAA CCCAGAGCTA ATACGTGA-- AACCGAAGCT
CCGACTGGCT AGAC--CCCA TTCCTCGTGG GTGGCGGTCG CTGGGACGAG CGGTTTAGTC
TGAACGAAAA CCATGTCGA- -------CTT C------GGT CGAC----TC TGTGGTGACT
CCGGGCTACA GTTGTCGGAT CGCAGTGCCC C-CTCGAGGG GCGGCGACGG -ATCTTTCAA
ATGTCTGACT CATCAAC--- TAGATGGTAG TAGCTTGGGC TACAATGGTG GTGACGGGTG
ACGGGGAATC AGGGTTTGAT TCCGGAAAGG AAGCCTGAGA AACGGCTACC ACATCTAAGG
AAGGCAGCAG GCGCGCAAAT TACCCACTCC CGGAACGGGG AGGTAGTGAC GAGAAATACC
GTTCGGG--- -----ACGAC CCCCCGCAAA GGGCGTGCCC GTCCGGAATG AGTACAGCGT
ACATCCCTGT ACGANGATCG ATTGGAGGGC AAGTCCTGGT GCCAGCAGCC GCGGTAATAC
CAGCTCCAGT AACGTATGCT AAAGTTGCTG CAGTCAAAAA GCTCGTCGTT GGATCTTGGC
CC----GGCG GCGGCGCGCG CGAT-CCGAG AAGGACGCGC GCGGGCGTCG GACGGGTG--
-AG------- ---------- ---------- ---------- ---------- ----------
-----ACTTG CCGGTG-TGA CCCGTCGGGA TGCCCTTCGT TG-GGCGTCT TCTTGACGAC

GGGCCGGCGC TGTTTACTTT GGACAAATGT TGGAGTTCAA AGCAGGCCT- TA--GTGGCC
GGAGACTGTT GCTTGGAATA ACGGAATTGG GCTTCGGCTC GTTTTC--GT TGGTTTTCGG
AG-GACGAAG CGTTGGTTAA AAGGGACTGA CGGAGGCATT CGTACTACGG GGCGAGAGGT
GAAATTCTGA GACCCTCGTA AGACGAACAA GAGCGAAAGC ATCTGCCTAG GATGTTTTCA
TTAATC-AAG AACGAAAGTC GGAGGAGCGA AGACGATTAG ATACCGTCGT AGTTCCGACC
ATANACGATG TCGACTAGCG ATCCGCCGCC GTTTTTTCAC AATGAACTCG GTGGGCAACT
CTCGCGGGAT ACCAAAGTGT TTGGATTCCG GGGGGAGTAT GGTCGCAAGG CTGAAACTTG
AAGGAATTGA CGGAAGGGCA CCACCAGGAG TGGAGCCTGC GGCT-TAATT TGACTCAACG
CGGGAAACCT CACCCGGCCC AGACACGGCA AGGATTGACA GATTGAAAGC TCTTTCTCGA
TTCGGTGGTT GGTGGTGCAT GGCCGTTCCT GGTTGGTGGA ACGATTTGTC TGGTCAATTC
CGATAACGAG CGAGACTCTA GCCTGCTCGG TAGACGGCAC GG------CC CACAAGCGGC
CGAGGTCGGT CAATACTTCT TAGAGGGACC AGCGTG-ACG ATGAAAAGAC GGTGCGAGAT
GGAGCTAAAA CAGGTCTGTG ATGCCCTTAG ATGTCTGGGG CCGCACGCGC GCTACACTGG
AGGAGACAGC GGGTTGTGAG -TTCCGACCG CTGCCGAGTG GAGTCGGCCG ACGGCGACGT
CGGCTCGACG TGTGGCGGCA CTCCCGTGAA CCTCCTCCGT GCTGGGGATT GGGCTTTGTG
ACTCTCGCCC ATGAACGAGG AATTCCCGGT AAGCACGAGT CACTAGCTCG TGTTGAACGC
GTCCCTGCCC TTTGTACACA CCGCCCGTCG CTACCGTTGG TCAGCTGGTT ACGCGAG---
--CGAGACGG ATCGGG---- ----GGCTCG CC-------- --------TC CCGCCAAGTC
GAGCAAGCTG TGACCGGTCT CGAAACGGT- AAAAGTCGTA ACAAGGTTTC CATTACTAAG
CGGAGGAAAA GAAACTAACG A-GGATTCCC TCAGTAACGG CGAGTGAAGC GGGA-AGAGC
CCAGCACCGA ATCCCCCC-G TCTGCCAAAG GC--GGC-GG GAGCTGTGGT GTGTGGGGCG
GTCCTCTGCC TTGTCCGGCG CTGCCTCTCC CAAGTGTACG TTGGAACTGG ACGAGCGGCA
GAATGCAATC TGCAC----- ---------- -----CCAGA GAGGGTGATA GGCCCGTGGG
GAGCGGGCGC CGGGCGGCG- --GTGTGG-- CTGCC-TCCA GAGTCGGGTT G-TTTGGGAA
TGCAGCCCAA AGCGGGTGGT AGACTCCATC CAAGGCTAAA TACGCTGGCG AGTCCGATAG
CAAA-CAAGT ACCGCGAGGG AAAGTTGAAA AGAAC-TTTG AAAAGAGAGT TCAAGAGTAC
GTGAAACCGT TCAGAGGTAA ACGGATGGAA TCGCAAACGC C--GCCGCGG ---GTCTCA-
---------- ---------- ---------- GTCCGGTGCG C--------- ------TTCG
GCGCGCC--- ---------- ---------- ---------- ---------- ---------G
GGTGTACTGC CCGCGGCGGG A-TGTCGCTC CCGGTGCT-- ---------- ---CCC----
------GCTG -CCGTGGT-- ---------- --TACCTCTC AAAGGCG--- --------TC
GTGGCTT-G- ---------- ---------- -------TAA AAG------- ------GGCC
GT----CGGC GCAGGGGG-- ---------- ---------- ---------- ---GTCG---
ACCG-----C AAGCGGCCGG -----CGGTT TTTTCGGGCA CCGGA----- ---------C
GAGCGGCGG- ---------- ---------- ---------- ---------- ----------
---------- ---GCT-TTT GAACGACCGC CCATCCGACC CGTCTTGAAA CACGGACCAA
GGAGTCTTGC ATGCGTGCGA GTCACGGGGA CCAGACAAGT CCCAC-CTGG GCGCAGAGAA
AGCGA----- ---------- -ACTGGCCGG AGGGCGCTCC GCCTC----- --GGCGGTGG
C------GCC TGCACGGCGG GCCCG----- ----CAG--C ATGAATG--- -TGGAGCCTG
AGCACGCATG TTGGTACCCG AAAGATGGTG AACTATGCCC GAGCAGGACG AAGCCATTGG
AAACTTTGGT GGAGGTCCGT AGCGGTTCCG CAAGTCGACC GGAGGCAAGG CGCCCAGAAA
GCAGCTGGCC ACCAAGGCTG CCCGCAAGAG CGCCCCGGCC ACGGGAGGCG TCAAGAAACC
GCACAGGTAC AGGCCCGGCA CCGTGGCCCT GCGTGAGATC CGCAGATACC AGAAGAGCAC
CGAGCTGCTC ATCCGCAAGC TGCCCTTCCA GCGACTCGTC CGCGAGATCG CCCAGGACTT
CAAGACCGAC CTGCGCTTCC AGAGCTCGGC CGTGATGGCG CTGCAGGAGG CGAGCGAGGC
CTACCTGGTC GGCCTCTTCG AANACACCAA CCTGTGTGCC ATCCACGCCA AGCGCAACCC
TATATTTTAT TCTCGCAGCA TGAGGTGGAC TACTAGGAAC ATCAATAAGA CTAATAATTC
GGATCGAACT AGGACAACCA GGATCCCTTC TAAATAGAGA TCAAATTTAC AATACAATTG
TAACAGCACA CGCATTCCTA ATAATTTTCT TTCTTGTAAT ACCTATATTT ATTGGAGGAT
TCGGAAATTG ACTCCTACCT CTTATATTAG GGGCACCAGA CATAGCATTC CCACGCCTCA
ATAACATAAG ATTTTGACTA CTACCCCCAG CTCTAATTAT ACTAGTGTCC TCCGCTATTG
TTGGAGATGG AGTAGGAACA GGATGAACAG TATACCCTCC CTTAGCAGGC AATATTGCTC
ACTCAGGCCC ATCAGTAGAT TTTGCTATTT TCTCTCTCCA CTTAGCAGGA ATCTCATCAA
TTCTAGGGGC CCTAAATTTC ATTACCACAG TAATTAATAT ACGATGGGAC GGACTAAAAA
TAGAACGTCT TCCTCTATTT GTATGGGCGG TATTAATTAC AGCTATCCTT CTTCTCCTGT
CACTCCCAGT ATTAGCAGGA GCTATTACTA TACTACTAAC AGACCGAAAC CTCAATACTT
CCTTCTTTGA CCCACAAGGA GGAGGGGACC CAGTTCTATA CCAACACCTA TTCT

>Ikeda_sp1
TAGTTATATG CTTATCTCAA AGATTAAGCC ATGCAGGTGC AAGTACCCGC TGCTTCTCGC
GAGAGACAGC AGCTAAACCG CGGATGGCTC AGTAAATCAG ACAGAGTTCA CTAGATGAAC
GCACCAG--T AGTCGGATAA CTGTGGAAAA CCCAGAGCTA ATACGTGA-- AACCGAAGCT
CCGACTGGCT AGACCTCCCG CTCCTCGTGG GTGGCGGTCG CTGGGACGAG CGGTTTAGTC
TGAACGAAAA CCATGTCGA- ------CTTC C-----GGGT CGAC----TC TGTGGTGACT
CCGGGCTACA GTTGTCGGAT CGCAGTGCCC CCTCCGAGGG GCGGCGACGG -ATCTTTCAA
ATGTCTGACT CATCAAC--- TAGATGGTAG TAGCTTGGGC TACAATGGTG GTGACGGGTG
ACGGGGAATC AGGGTTCGAT TCCGGAGAGG AAGCCTGAGA AACGGCTACC ACATCTAAGG
AAGGCAGCAG GCGCGCAAAT TACCCACTCC CGGAACGGGG AGGTAGTGAC GAGAAATACC
GTTCGGG--- -----ACGA- CCCCCGCAAA GGGCGTGCCC GTCCGGAATG AGTACAGCGT
ACATCCCTGT ACGAGGATCG ATTGGAGGGC AAGTCCTGGT GCCAGCAGCC GCGGTAATAC
CAGCTCCAGT AGCGTATGCT AAAGTTGCTG CAGTCAAAAA GCTCGTCGTT GGATCTTGGC
CC----GGCG GCGGCGCGCG CGATCCCGAG GAGGACGCGC GCGGGCGTCG GACGGGTG--
-AG------- ---------- ---------- ---------- ---------- ----------
-----ACTTG CCGGTG-TGA CCCGTCGGGA TGCCCTTCGT TG-GGCGTCT TCTTGACGAC
GGGCCGGCGC TGTTTACTTT GGACAAATGT TGGAGTTCAA AGCAGGCCT- TA--GTGGCC
GGAGACTGTT GCTTGGAATA ACGGAATTGG GCTTCGGCTC GTTTTC--GT TGGTTTTCGG

AG-GACGAAG CGTTGGTTAA AAGGGACTGA CGGAGGCATT CGTACTACGG GGCGAGAGGT
GAAATTCTGA GACCCTCGTA AGACGAACAA GAGCGAAAGC ATCTGCCTAG GATGTTTTCA
TTAATC-AAG AACGAAAGTC GGAGGAGCGA AGACGATTAG ATACCGTCGT AGTTCCGACC
ATAAACGATG TCGACTAGCG ATCCGCCGCC GTTTTTTCAC AATGAACTCG GTGGGCAACT
CTCGCGGGAT ACCAAAGTGT TTGGATTCCG GGGGGAGTAT GGTCGCAAGG CTGAAACTTG
AAGGAATTGA CGGAAGGGCA CCACCAGGAG TGGAGCCTGC GGCT-TAATT TGACTCAACG
CGGGAAACCT CACCCGGCCC AGACACGGCA AGGATTGACA GATTGAAAGC TCTTTCTCGA
TTCGGTGGTT GGTGGTGCAT GGCCGTTCCT GGTTGGTGGA ACGATTTGTC TGGTCAATTC
CGATAACGAG CGAGACTCTA GCCTGCTCGG TAGACGGCAC GG------CC CACAAGCGGC
CGAGGTCGGT CAATACTTCN TAGAGGGANC AGCGTG-ACG ATGAAAAGAC GGTGCGAGAT
GGAGCTAAAA CAGGTCTGTG ATGCCCTTAG ATGTCTGGGG CCGCACGCGC GCTACACTGG
AGGAGACAGC GGGTTGTGAG -TTCCGACCG CTGCCGAGTG GAGTCGGCCG ACGGCGACGT
CGGCTCGACG TGTGGCGGCA CTCCCGTGAA CCTCCTCCGT GCTGGGGATT GGGCTTTGTG
ACTCTCGCCC ATGAACGAGG AATTCCCGGT AAGCACGAGT CACTAGCTCG TGTTGAACGC
GTCCCTGCCC TTTGTACACA CCGCCCGTCG CTACCGTTGG TCAGCTGGTT ACGCGAG---
--CGAGACGG ATCGGG---- ----GGCTCG CC-------- --------TC CCGCCAAGTC
GAGCAAGCTG TGACCGGTCT CGAAACGGT- AAAAGTCGTA ACAAGGTTTC CATTACTAAG
CGGAGGAAAA GAAACTAACG A-GGATTCCC TCAGTAACGG CGAGTGAAGC GGGA-AGAGC
CCAGCGCCGA ATCCCCCC-G TCTCCCAAAG GC--GGC-GG GAGCTGTGGC GTGTGGGGCG
GTCCTCTGCC TTGTCCGGCG CTGCCTCTCC CAAGTGTACG TTGGAAGTGG ACGAGCGGCA
GAATGCAATC TGCAC----- ---------- -----CCAGA GAGGGTGATA GGCCCGTGGG
GAGCGGGCGC CGGGCGGCG- --GTGTGG-- CTGCC-TCCA GAGTCGGGTT G-TTTGGGAA
TGCAGCCCAA AGCGGGTGGT AGACTCCATC CAAGGCTAAA TACGCTGGCG AGTCCGATAG
CAAA-CAAGT ACCGCGAGGG AAAGTTGAAA AGAAC-TTTG AAAAGAGAGT TCAAGAGTAC
GTGAAACCGT TCAGAGGTAA ACGGATGGAA TCGCAAACGC CG-CCCGCGG ---GTCTCA-
---------- ---------- ---------- GTCCGGCACG C--------- -A--------
---------- ---------- AGTGCC---- ---------- ---------- ---------G
GGCGTACTGC CCGCGGCGGG A-TGTCGCTC CCGGTGCT-- ---------- ---CCC----
------GCTG -CCGTGGTC- ---------- --TTCCCCTC AAAGGCG--- --------CC
GTGGCTC--- ---------- -----TTCGC GAGAGGAC-- ---------- ------GCCG
TC----TG-C GCGGGGGGGA T--------- ---------- ---------- ---GCCG---
CCTG------ -AGCGGTT-- ---TGCGGGG --TTTCGGCA CCGGG----- ---------C
GAGCGGCGG- ---------- ---------- ---------- ---------- ----------
---------- ---GCTTTTC GAACGACCGC CCATCCGACC CGTCTTGAAA CACGGACCAA
GGAGTCTTGC ATGCGTGCGA GTCACGGGGA CCGGACAAGT CCCGCTCGAG GCGCAGAGAA
AGCGA----- ---------- -ACTGGCCGG AGGCCGGCCA CA------AA AGGGCCG--G
C--------- TGCACGGCGG GCCCG----- ----CAG--C ACGAATG--- -TGGAGCCCG
AGCACGCATG TTGGTACCCG AAAGATGGTG AACTATGCCC GAGCAGGACG AAGCCATTGG
AAACTTTGGT GGAGGTCCGT AGCGGTTCCG AAAGTCGACC GGAGGCAAGG CGCCCAGAAA
GCAGCTGGCC ACCAAGGCTG CCCGCAAGAG CGCCCCGGCT ACCGGAGGCG TCAAGAAACC
GCACAGGTAC AGGCCCGGCA CCGTGGCCCT CCGTGAGATC CGCAGATACC AGAAGAGCAC
AGAGCTGCTC ATTCGCAAGC TGCCATTCCA GCGACTCGTC CGCGAGATAG CCCAAGACTT
CAAGACGGAC CTGCGCTTCC AGAGCTCGGC CGTGATGGCT CTGCAGGAGG CGAGCGAGGC
CTATCTAGTC GGTCTCTTTG AAGACACCAA CCTGTGCGCC ATCCACGCCA AGCGCAACTC
TATACTTTAT CCTAGCCGCA TGAGGAGGAT TACTAGGAAC CTCTATAAGA TTAATAATTC
GAATCGAACT AGGACAACCC GGATCCCTTC TAAATAGAGA CCAAATTTAC AATACAATTG
TAACAGCACA TGCATTCTTA ATAATTTTCT TTTTAGTTAT ACCCATATTT ATTGGAGGTT
TCGGAAACTG ACTACTCCCA TTAATACTAG GGGCCCCAGA CATAGCCTTC CCTCGACTAA
ATAACATAAG ATTTTGACTT CTACCTCCAG CCCTAATCAT ACTAGTATCA TCAGCTATTG
TTGGTGATGG GGTAGGCACT GGATGAACAG TATATCCACC TCTAGCAGGA AACATTGCCC
ACTCAGGACC ATCGGTAGAT TTCGCCATCT TCTCTCTACA CCTAGCAGGG GTCTCATCTA
TTCTAGGAGC TCTTAATTTC ATCACCACAG TAATTAACAT GCGATGAGAA GGACTAAAAA
TAGAGCGACT ACCTCTATTC GTCTGAGCTG TTCTAATTAC AGCTATTCTC TTACTACTTT
CTCTCCCCGT TCTAGCTGGA GCTATTACCA TACTCCTCAC AGACCGTAAC CTCAATACCT
CATTTTTTGA TCCTCAAGGT GGAGGAGACC CAGTTCTCTA TCAACACCTA TTCT

>Dasybranchus_sp
TAGTCATATG CTTGTCTCAA AGATTAAGCC ATGCATGTCT AAGTACAAAC TTTTAC----
--------AC AGTGAAACTG CGAATGGCTC ATTAGATCAG TTATGGTTCC TTAGAT---C
GTACAATCCT ACTTGGATAA CTGTGGTAAT TCTAGAGCTA ATACATGA-- AAGC-ACGCT
CCGACTCCTC ---------- ---------- ---------- CGAGGAAGAG CGTTTTTAT-
TAGAACAAAA CCA-ATCGG- --CGTTCGCT CACGCGTCGC CGTC----CC ATTGGTGACT
CTGAATAACT TTGTGCTGAT CGC-ATGGCC T-T----GCG CCGGCGACGT GATCTTTCAA
ATGTCTGCCC TATCAAG-TC TCGATGTTAG TTGACATGAC TATCATGCTG ATAACGGGTA
ACGGGGAATC AGGGTTCGAT TCCGGAGAGG GAGCATGAGA AACGGCTACC ACATCCAAGG
AAGGCAGCAN GCNCGCAAAT TACCCACTCC TGACACGGGG AGGTAGTGAC GAAAAATAAC
AANACGGGAC TCTT-TCGAG GCCCCGTAA- ---------- --TTGGAATG AGTACACTTT
AAATCCTTTA ACGAGGATCT ATTGGAGGGC AAGT-CTGGT GCCAGCAGCC GCGGTAATTC
CAGCTCCAAT AGCGTATATT AAAGTTGCTG CAGTTAAAAA GCTCGTAGTT GGATCTCGGG
T-TTAGGCCG GCGGTTCAC- ---------- ---------C TCA-TTGGT- GATCACTGCC
-TG------- ---------- ---------- ---------- ---------- ---TCCTGAC
CT---ACCTG CCGGTT-T-- TCCCCT--GG TGCTCTTCGC TG-AGTGTCT GG------GG
TGGCTGGAAC -GTTTACTTT GAAAAAATTA GAGTGTTCAA AGCAGGCCTT TC--TAGCCT
GAATAATAGT GCATGGAATA ATGGAAGAGG ACCTCGGTTC TATTTT--GT TGGTTTTCGG
AA-CTCGAGG TAATGATTAA GAGGGACTGA CGGGGGCATT CGTATTACGG TGTTAGAGGT
GAAATTCTTG GATCGTCGTA AGACGAACTA CTGCGAAAGC ATTTGCCAAG AATGTTTTCA

```
TTAATC-AAG AACGAAAGTC GGAGGTTCGA AGACGATCAG ATACCGTCGT AGTTCCGACC
TTAAACGATG CCAACTGGCA ATTGGCCGGC GTTGTTTCAA ---TGACCCG GCGGGCAGCT
TC--CGGGAA ACCAAAGTCT TTGGGTTCCG GGGGAAGTAT GGTTGCAAAG CTGAAACTTA
AAGGAATTGA CGGAAGGGCA CCACCAGGAG TGGAGCCTGC GGCT-TAATT TGACTCAACA
CGGGAAAACT CACCCGGCCC GGACACTGTA AGGATTGACA GATTGAGAGC TCTTTCTTGA
TTCGGTGGGT GGTGGTGCAT GGCCGTTCTT AGTTGGTGGA GCGATTTGTC TGGTTAATTC
CGATAACGAA CGAGACTCTA GCCTATTAAA TAGTTCGCCG AT-------- CCCGTCTCGT
CGGCGTTA-- ----ACTTCT TAGAGGGACA AGTGTC---- GTTTAGGCAC ---GCGAGAT
TGAGCAATAA CAGGTCTGTG ATGCCCTTAG ATGTTTGGGG CCGCACGCGC GCTACACTGA
AGGACTCAAC GTGT-----G -TTTGTCCTG GC-TCGGGAG GGCT------ ----------
---------- -----GGGTA ACCCGTTGAA CCTCCTTCGT GCTAGGGATT GGGGCTTGTA
ATTCTTCCCC ATGAACGAGG AATTCCCAGT AAGCGCGAGT CATCAGCTCG CGTTGATTAC
GTCCCTGCCC TTTGTACACA CCGCCCGTCG CTACTACCGA TTGGATGGTT TAGTGAGATC
CTCGGATTGG TCCCGG--CA TGGTGGGTTA CC--GCCGCG CCGGC--GTG CCGAAAAGAC
GATCAAACT- TGATC-ATCT AGAGGAAGT- AAAAGTCGTA ACAAGGTTTC CATTACTAAG
CGGAGGAAAA GAAACTAACT A-GGATTCCC CCAGTATCGG CGAGTGAAGT GGGA-AGAGC
CCAGCACCGA ATCCCCCG-C CATGC---AG GC--GGC-GG GAACTGTGGT GTTCGGGACG
T-GCTCTGCG TCGTGCG-TC GGGCGTCT-- ---GAGTCCT CTTGATAGAG G---------
---------C TTTAT----- ---------- -----CCATA GAGGGTGTCA GACCCGTGC-
GACTCCTGG- -CGCCGGCG- --CGTTGC-- GCGTCTCTTA GAGTCGGGTT G-TTTGGGAA
TGCAGCCCAA ATAGGGTGGT AAACTCCATC TAAGGCTAAA TACGGTCGCG AGTCCGATAG
CAGA-CAAGT ACCGTGAGGG AAAGTTGAAA AGAAC-TTTG AAGAGAGAGT TCAAGAGTAC
GTGAAACCGT TCAGAGGCAA ACGGATGGGA CCGCAACCTC C--CCCGCGG ---AATTCAA
CCTCCGGGCC -G--CTCGTC CCGGCGCGT- CGTCGAT-CG C--------- -A--A-GACG
GCGCGCGTCG ---------- GGAGCGGGC- ---------- ---------- ---GTTCGGG
GGCGCATTTT TCGCGGGGGG A-CGCCGCGA CCGGTTCCGC GCCAGTCAGA AG-CCC----
------GGCG -GGAAGGTAG CCTG------ --TC---TTC GGA---G--- ---AGGT-GT
TTAGCCC-G- --CCGGCGTG TCGGCCTGGC T-CGGGACCG AGG------G AC----GCCC
GC----CG-C GGCGCGGGCC C--TC----- ------GCTC CTTCCGCCCG ---TTCG---
ACTG-----G GAGGGACTGT TC-T-CAGTG CCTTCCGACC GCGAG----- -CGGATG--C
GGGACGGGG- ---------- ------ACTC CCCGCGTCG- ---------- -----ACTTT
AG---GGTCT GCGGCTGCTC GGTCGGCTCC CTATCCGACC CGTCTTGAAA CACGGACCAA
GGAGTCTAAC ATGTGCGCGA GTCAT-TGGA CT-TACGAAA TCTA---AAG GCGCAATGAA
GGTGA-AGGC CCTC-CTTAA GGTGGGCCTA GGTCGGATCC CT-CCCCTAG CGGGGGG--G
C--------- -GCAAGACTG GCCCGTCTCA GAGGCAT--T GCCCTCGAGG -CGGAGCAAG
AGCGTACACG TTGGGACCCG AAAGATGGTG AACTATGCCT GAGTAGGACG AAGCCAGAGG
AAACTCTGGT GGAGGTCCGT AGCGATTC?? ?????????? ?????????? ??????????
?????????? ?????????? ?????????? ?????????? ?????????? ??????????
?????????? ?????????? ?????????? ?????????? ?????????? ??????????
?????????? ?????????? ?????????? ?????????? ?????????? ??????????
?????????? ?????????? ?????????? ?????????? ?????????? ??????????
?????????? ?????????? ?????????? ?????????? ?????????? ??????????
?????????? ?????????? ?????????? ?????????? ?????????? ??????????
?????????? ?????????? ?????????? ?????????? ?????????? ??????????
?????????? ?????????? ?????????? ?????????? ?????????? ??????????
?????????? ?????????? ?????????? ?????????? ?????????? ??????????
?????????? ?????????? ?????????? ?????????? ?????????? ??????????
?????????? ?????????? ?????????? ?????????? ?????????? ??????????
?????????? ?????????? ?????????? ?????????? ?????????? ??????????
?????????? ?????????? ?????????? ?????????? ?????????? ??????????
?????????? ?????????? ?????????? ?????????? ?????????? ??????????
?????????? ?????????? ?????????? ?????????? ?????????? ??????????
?????????? ?????????? ?????????? ?????????? ?????????? ????

>Heteromastus_filiformis
---------- ---------- ---------- ---------- -----CAAAC TTTTAC----
--------AC AGTGAAACTG CGAATGGCTC ATTAGATCAG TTATGGTTCC TTAGAT---C
GTACAATCCT ACTTGGATAA CTGTGGTAAT TCTAGAGCTA ATACATGA-- AAGC-ACGCT
CCGACTTTCG ---------- ---------- ---------- --GGGAAGAG CGTTTTTAT-
TACAACAAAA CCA-ATCGG- ------CCTT C-----GGGT CGTC----CA ATTGGTGACT
CTGAATAACT TTGTTCTGAT CGC-ATGGCC T-C----GAG CCGGCGACGT -ATCTTTCAA
ATGTCTGCCC TATCAAG-TC TCGATGTTAG TTGACTTGAC TATCATGCTT GTAACGGGTA
ACGGGGAATC AGGGTTCGAT TCCGGAGAGG GAGCATGAGA AACGGCTACC ACATCCAAGG
AAGGCAGCAG GCGCGCAAAT TACCCACTCC TGACACGGGG AGGTAGTGAC GAAAAATAAC
AATACGGGAC TCAT-TCGAG GCCCCGTAA- ---------- --TTGGAATG AGTACACTTT
AAATCCTTTA ACGAGGATCC ATTGGAGGGC AAGT-CTGGT GCCAGCAGCC GCGGTAATTC
CAGCTCCAAT AGCGTATA-T AAAGTTGTTG CAGTTAAAAA GCTCGTAGTT GGATCTCGGG
T-TTAGGCTG GCGGTCCGC- ---------- ---------- CCAGTCGGT- GGTCACTGCC
-TG------- ---------- ---------- ---------- ---------- ---TCCTGAC
CT---ACCTG CCGGTC-T-- TCCCCT--GG TGCTCTTCGC TG-AGTGTCT GG------GG
CGGCCGGGAC -GTTTACTTT GAAAAAATTA GAGTGTTCAA AGCAGGCGT- TC--TCGCCT
GAATAATAGT GCATGGAATA ATGGAAGAGG ACCTCGGTTC TATTTT--GT TGGTTTTCGG
AA-CTGGAGG TAATGATTAA GAGGGACTGA CGGGGGCATT CGTATTATGG TGTTAGAGGT
GAAATTCTTG GATCGTCGTA AGACGAACTA CTGCGAAAGC ATTTGCCAAG AATGTTTTCA
TTAATC-AAG AACGAAAGTC GGAGGTTCGA AGACGATCAG ATACCGTCGT AGTTCCGACC
TTAAACGATG CCAACTAGCA ATTCGCCGGC GTTGTTTCCA ---TGACTCG GCGGGCGGCT
```

TC--CGGGAA ACCAAAGTCT TTGGGTTCCG GGGGAAGTAT GGTTGCAAAG CTGAAACTTA
AAGGAATTGA CGGAAGGGCA CCACCAGGAG TGGAGCCTGC GGCT-TAATT TGACTCAACA
CGGGAAAACT CACCCGGCCC GGACACTGTA AGGATTGACA GATTGAGAGC TCTTTCTTGA
TTCGGTGGGT GGTGGTGCAT GGCCGTTCTT AGTTGGTGGA GCGATTTGTC TGGTTAATTC
CGATAACGAA CGAGACTCTA GCCTATTAAA TAGTTCACCG AT-------- -CTGTCTTGT
CGGTGTCA-- ----ACTTCT TAGAGGGACA AGTGTC---- GTATAGGCAC ---ACGAGAT
TGAGCAATAA CAGGTCTGTG ATGCCCTTAG ATGTTTGGGG CCGCACGCGC GCTACACTGA
AGGACTCAAC GTGT-----G -TCTCACCTG GT-CTGGGAG GACT------ ----------
---------- -----GGGTA ACCCGTTGAA CCTCCTTCGT GCTAGGGATT GGGGCTTGTA
ATTCTTCCCC ATGAACGAGG AATTCCCAGT AAGCGCGAGT CATCAGCTCG CGTTGATTAC
GTCCCTGCCC TTTGTACACA CCGCCCGTCG CTACTACCGA TTGGATGGTT TAGTGAGACC
CTCGGATTGG ACCCGG--CA TGGTGGGTTA CC--GCCGCG CCGGC--GTG CCGAGAAGAC
GTTCAAACT- TGATC-ATCT AGAGGAAGT- AAAAGTCGTA ACAAGGTTTC CATTACTAAG
CGGAGGAAAA GAAACTAACT A-GGATTCCC TCAGTAACGG CGAGTGAAGT GGGA-AGAGC
CCAGCACCGA ATCCCCCG-C CATGC---TG GC--GGC-GG GAACTGTGGT GTTTGGGACG
G-GCTCTGCG ACGTCCG-GC AGCTGTCT-- ---GAGTCCT CTTGATAGAG G---------
---------C TTTAT----- ---------- -----CCATA GAGGGTGTCA GACCCGTGC-
GACTTCTGC- -CGGCGGCG- --CGCTGT-- CCGTC-CTTA GAGTCGGGTT G-TTTGGGAA
TGCAGCCCAA ATCAGGTGGT AAACTCCATC CAAGGCTAAA TACGGTCGCG AGTCCGATAG
CGGA-CAAGT ACCGTGAGGG AAAGTTGAAA AGAAC-TTTG AAGAGAGAGT TCAAGAGTAC
GTGAAACCGT TCAGAGGTAA ACGGATGGGA CCGCAACCTC C--CCCGTGG ---AATTCAA
CCCTTGGCGC -G--CCGGTC GTGTCGGAC- GTCGGAT-CT T--------- -A--ACGACC
GGCCCCGGCC ---------- CGAGCCGGC- ---------- ---------- ---CGTCGGG
GGCGCATTTT CCG--GGGGT G-AGCCACGA CCGGTTCGCC GTCAGTCAGA AG-CCC----
------GCCG -GGAAGGTGA CCTG------ --CC---TTC GGG---T--- ---AGGT-GT
ATAGCCC-G- --GTGGCGTG TTGGCCTGAT G-GCGGACTG AGG------A AC----GCCC
GC----CG-C TGGAGGGGCC C--CC----- ------GGCC CGCCTGCCCG ---TTCG---
ACTG-----T GAGGGACTGT TG-C-CAGTG CCTTGCGACC GCGGG----- -TTTGCG--C
GCT-CGGGG- ---------- ------ATCA CCCCTCCCG- ---------- -----CATTC
A----GGTCT GTGGCTGCTC GGTCGGCTCC CTATCCGACC CGTCTTGAAA CACGGACCAA
GGAGTCTAAC ATGTGTGCGA GTCAT-GGGG TTCTACGAAA CCTA---AAG ACGCAATGAA
AGTGA-AGGT CTTC-CTCTT GGTTGGCCTA GGTCGGATCC C--TCCTTCG -G---GG--G
C--------- -GCAAGACTG GCCCGTCTCA GGGGCAT--C GTTCCCGAGG -CGGAGC-AG
AGTACATACG TTGGGACCCG AAAGATGGTG AACTATGCCT GAGTAGGACG AAGCCAGAGG
AAACTCTGGT GGAGGTCCGT AGCGATTC?? ?????????? ?????????? ??????????
?????????? ?????????? ?????????? ?????????? ?????????? ??????????
?????????? ?????????? ?????????? ?????????? ?????????? ??????????
?????????? ?????????? ?????????? ?????????? ?????????? ??????????
?????????? ?????????? ?????????? ?????????? ?????????? ??????????
?????????? ?????????? ?????????? ?????????? ?????????? ??????????
?????????? ?????????? ?????????? ?????????? ?????????? ??????????
?????????? ?????????? ?????????? ?????????? ?????????? ??????????
?????????? ?????????? ?????????? ?????????? ?????????? ??????????
?????????? ?????????? ?????????? ?????????? ?????????? ??????????
?????????? ?????????? ?????????? ?????????? ?????????? ??????????
?????????? ?????????? ?????????? ?????????? ?????????? ??????????
?????????? ?????????? ?????????? ?????????? ?????????? ??????????
?????????? ?????????? ?????????? ?????????? ?????????? ??????????
?????????? ?????????? ?????????? ?????????? ?????????? ??????????
?????????? ?????????? ?????????? ?????????? ?????????? ??????????
?????????? ?????????? ?????????? ?????????? ?????????? ????

>Notomastus_tenuis
TAGTCATATG CTTGTCTCAA AGATTAAGCC ATGCATGTCT AAGTACATAC TTTTAC----
--------AC AGTGAAACTG CGAATGGCTC ATTAGATCAG TTATGGTTCC TTAGAT---C
GTACAATCCT ACTTGGATAA CTGTGGTAAT TCTAGAGCTA ATACATGA-- AAGC-ACGCT
CCGACTCTCT ---------- ---------- ---------- AGGGGAAGAG CGTTTTTAT-
TACAACAAAA CCA-ATCGG- ------CCTT C-----GGGT CGTC----CC TTTGGTGACT
CTGAATAACT TTGTTCCGAT CGC-ATGGCC T-C----GAG CCGGCGACGT -ATCTTTCAA
ATGTCTGCCC TATCAAG-TT TCGATGTTAG TTGACATGAC TATCATGCTT TTAACGGGTA
ACGGGGAATC AGGGTTCGAT TCCGGAGAGG GAGCATGAGA AACGGCTACC ACATCCAAGG
AAGGCAGCAG GCGCGCAAAT TACCCACTCC TGACACGGGG AGGTAGTGAC GAAAAATAAC
AATACGGGAC TCGT-TCGAG GCCCCGTAA- ---------- --TTGGAATG AGTACACTTT
AAATCCTTTA ACGAGGATCT ATTGGAGGGC AAGT-CTGGT GCCAGCAGCC GCGGTAATTC
CAGCTCCAAT AGCGTATATT AAAGTTGTTG CAGTTAAAAA GCTCGTAGTT GGATCTCGGG
T-TTAGGCCG GCGGTTCAC- ---------- ---------C TCG-TCGGT- GACGACTGCC
-TG------- ---------- ---------- ---------- ---------- ---TCCTGAC
CT---ACCTG CCGGTC-T-- TCCTCT--GG TGCTCTTGAC TG-AGTGTCT GA------GG
CGGCCGGAAC -GTTTACTTT GAAAAAATTA GAGTGTTCAA GGCAGGCGT- TT--TTGCCT
GAATAATAGT GCATGGAATA ATGGAAGAGG ACCTCGGTTC TATTTT--GT TGGTTTTCGG
AA-CTGGAGG TAATGATTAA GAGGGACTGA CGGGGGCATT CGTATTACGG TGTTAGAGGT
GAAATTCTTG GATCGTCGTA AGACGAACTA CTGCGAAAGC ATTTGCCAAG AATGTTTTCA
TTAATC-AAG AACGAAAGTC GGAGGTTCGA AGACGATCAG ATACCGTCGT AGTTCCGACC
TTAAACGATG CCAACTAGCA ATTCGCCGGC GTTGTTTCCA ---TGACTCG GCGGGCGGCT
TC--CGGGAA ACCAAAGTCT TTGGGTTCCG GGGGAAGTAT GGTTGCAAAG CTGAAACTTA
AAGGAATTGA CGGAAGGGCA CCACCAGGAG TGGAGCCTGC GGCT-TAATT TGACTCAACA

CGGGGAAACT CACCCGGCCC GGACACTGTA AGGATTGACA GATTGAGAGC TCTTTCTTGA
TTCGGTGGGT GGTGGTGCAT GGCCGTTCTT AGTTGGTGGA GCGATTTGTC TGGTTAATTC
CGATAACGAA CGAGACTCTA GCCTATTAAA TAGTTCGCCG AT-------- -TTTTCGAGT
CGGTGTCA-- ----ACTTCT TAGAGGGACA AGTGTC---- GTATAGGCAC ---GCGAGAT
TGAGCAATAA CAGGTCTGTG ATGCCCTTAG ATGTTTGGGG CCGCACGCGC GCTACACTGA
AGGACTCAAC GTGT-----G -TCTGTCCTG GC-CCGGGAG GGCT------ ----------
---------- -----GGGTA ACCCGTTGAA CCTCCTTCGT GCTAGGGATT GGGGCTTGTA
ATTCTTCCCC ATGAACGAGG AATTCCCAGT AAGCGCGAGT CATCAGCTCG CGTTGATTAC
GTCCCTGCCC TTTGTACACA CCGCCCGTCG CTACTACCGA TTGGATGGTT TAGTGAGATC
CTCGGATTGG ACCCTG--CA TGGTGGGTTA CC--GCCGCG CCGGC--GTG CCGAGAAGAC
GATCAAACT- TGATC-ATCT AGAGGAAGT- AAAAGTCGTA ACAAGGTTTC CATCAATAAG
CGGAGGAAAA GAAACTAACA A-GGATTCCC CTAGTAACGG CGAGCGAAGC GGGA-ATAGC
CCAGCACCGA ATCCCCTG-C CTCGC---AG GT--AGC-GG GAACTGTGGT GTTTAGGACT
G-CCTATGGG GTGGTGT-TC GGACGTCC-- ---GAGTCCT CCTGATCGGG G---------
---------C CTTTC----- ---------- -----CCATA GCGGGTGTCA GGCCTCTACT
GGCGATCGT- -CGCCTCCC- --CTTTGT-- GCGTC-CTTG GAGTCGGGTT G-TTTGGGAA
TGCAGCCCAA AGTGGGTGGT AAACTCCATC TAAGGCTAAA TACTGACACG AGACCGATAG
CGGA-CAAGT ACCGTGAGGG AAAGTTGAAA AGAAC-TTTG AAGAGAGAGT TCAAGAGTAC
GTGAAACCGT TCAGAGGTAA ACGGATGGGA CCGCAACCTC C--CCTGTGG ---AATTCAA
CCTTTGGGCA -G--TCTGTC CCGTCGGTT- TCCGGAT-CG C--------- -A--A-GACC
GGGGCCGGCG ---------- GGAGCAGGC- ---------- ---------- ---GTCCGGG
GGCGCATTTT TCGCAGGGGG A-AGCCGCGA CCGGTTCGGT GTCGGTCATA AGCCCC----
------GGCG -GGAAGGT-A GCTC------ --TC---CTC GGA---G--- ---AGTG-TT
ATAGCCC-G- --CGGAGGTG TTGGCCCGGC G-CCGGACCG AGG------G AC----GCCC
GC----CG-C GGCGGGGGCC C--TT----- ------GCCC CGTACGCTCG ---TTCG---
ACTG-----G GAGGGACTGT CC-T-CAGTG CCTCCCGACC GCGAG----- -CGGACG--T
GGG-TCGGG- ---------- ------ATTC CCCCCGTCG- ---------- -----TCTTT
AG---GGTCT GCGGCCGTTC GGTCGGCTCC CTATCCGACC CGTCTTGAAA CACGGACCAA
GGAGTCTAAC ATGTGCGCGA GTCAT-GGGG CTCTACGAAA CCTA---AAG GCGCAATGAA
GGTGA-AGGC CCTC-CTCTA GGTGGGCCTA GGTCGGATCC CG-TCCCCTG -GGGCGG--G
C--------- -GCAAGACTG GCCCGTCTCG AAGGCAT--T GACCTTGAGG -CGGAGCAAG
AGCGTACACG TTGGGACCCG AAAGATGGTG AACTATGCCT GAGTAGGACG AAGCCCGAGG
AAACTCTGGT GGAGGTCCGT AGCGATTC?? ?????????? ?????????? ??????????
?????????? ?????????? ?????????? ?????????? ?????????? ??????????
?????????? ?????????? ?????????? ?????????? ?????????? ??????????
?????????? ?????????? ?????????? ?????????? ?????????? ??????????
?????????? ?????????? ?????????? ?????????? ?????????? ??????????
?????????? ?????????? ?????????? ?????????? ?????????? ??????????
?????????? ?????????? ?????????? ?????????? ?????????? ??????????
?????????? ?????????? ?????????? ?????????? ?????????? ??????????
?????????? ?????????? ?????????? ?????????? ?????????? ??????????
?????????? ?????????? ?????????? ?????????? ?????????? ??????????
?????????? ?????????? ?????????? ?????????? ?????????? ??????????
?????????? ?????????? ?????????? ?????????? ?????????? ??????????
?????????? ?????????? ?????????? ?????????? ?????????? ??????????
?????????? ?????????? ?????????? ?????????? ?????????? ??????????
?????????? ?????????? ?????????? ?????????? ?????????? ??????????
?????????? ?????????? ?????????? ?????????? ?????????? ??????????
?????????? ?????????? ?????????? ?????????? ?????????? ????

>Ophelina_acuminata
TAGTCATATG CTTGTCTCAA AGATTAAGCC ATGCATGTCT AAGTACAAAC TTTTAC----
--------AC AGTGAAACTG CGAATGGCTC ATTAAATCAG TTATGGTTCC TTAGAT---C
GTACAAGT-T ACTTGGATAA CTGTGGTAAT TCTAGAGCTA ATACATGC-- AACA-AAGCT
CCGACCCTCG ---------- ---------- ---------- -TGGGAAGAG CGCTTTTAT-
TAGATCAAAA CCA-ATCGG- ------TCCT C-TCGGGGGC CGTC----CC ATTGGTGACT
CTGGATAACT TTGGGCTGAT CGC-ACGGCC T-T----GAG CCGGCGACGT -ATCTTTCAA
ATGTCTGCCC TATCAAC-TT TCGATGGTAC GTGATATGCC TACCATGGTT GTAACGGGTA
ACGGGGAATC AGGGTTCGAT TCCGGAGAGG GAGCATGAGA AACGGCTACC ACATCCAAGG
AAGGCAGCAG GCGCGCAAAT TACCCACTCC TGACACGGGG AGGTAGTGAC GAAAAATAAC
AATACGGGAC TCTT-TCGAG GCCCCGTAA- ---------- --TTGGAATG AGTACACTTT
AAATCCTTTA ACGAGGATCC ATTGGAGGGC AAGT-CTGGT GCCAGCAGCC GCGGTAATTC
CAGCTCCAAT AGCGTATATT AAAGTTGTTG CAGTTAAAAA GCTCGTAGTT GGATCTCAGG
T-GCAGGCTG GCGGTCCAC- ---------- ---------T TTG--CGGTG G--TACTGCC
-TG------- ---------- ---------- ---------- ---------- ---TCCTGAC
CT---ACCTC CCGGTT-T-- TCCCTT--GG TGCTCTTGAC TG-AGTGCCT CG------GG
TGGCCGGAAC -GTTTACTTT GAAAAAATTA GAGTGTTCAA AGCAGGCGTT ----GTGCCT
GAATATTCGT GCATGGAATA ATGGAATAGG ACCTCGGTTC TATTTT--GT TGGTTTTCGG
AA-CTTGAGG TAATGATTAA GAGGGACAGA CGGGGGCATT CGTATTACGG TGTTAGAGGT
GAAATTCTTG GATCGCCGTA AGACGAACTA CTGCGAAAGC ATTTGCCAAG AATGTTTTCA
TTAATC-AAG AACGAAAGTC AGAGGTTCGA AGACGATCAG ATACCGTCGT AGTTCTGACC
ATAAACGATG CCGACTAGCG ATCCGCCGGA GTTGTTTCCA ---TGACTCG GCGGGCAGCT
TC--CGGGAA ACCAAAGTCT TTGGGTTCCG GGGGAAGTAT GGTTGCAAAG CTGAAACTTA
AAGGAATTGA CGGAAGGGCA CCACCAGGAG TGGAGCCTGC GGCT-TAATT TGACTCAACA
CGGGAAAACT CACCCGGCCC GGACACTGTA AGGATTGACA GATTGAGAGC TCTTTCTTGA
TTCGGTGGGT GGTGGTGCAT GGCCGTTCTT AGTTGGTGGA GCGATTTGTC TGGTTAATTC

```
CGATAACGAA CGAGACTCTA GCCTATTAAA TAGTTCACCG AT-------- ATCGTTTTGT
CGGTGTTA-- ----ACTTCT TAGAGGGACA AGTGGC---- TTTCAGCCAC ---ACGAGAT
TGAGCAATAA CAGGTCTGTG ATGCCCTTAG ATGTTCGGGG CCGCACGCGC GCTACACTGA
AGGAATCAGC GTGT-----G -TTTGCCCTG GC-CCGGAAG GGTC------ ----------
---------- -----GGGTA ACCCGTTGAA CCTCTTTCGT GCTAGGGATT GGGGCTTGTA
ATTGTTCCCC ATGAACGAGG AATTCCCAGT AAGCGCGAGT CATAAGCTCG CGTTGATTAC
GTCCCTGCCC TTTGTACACA CCGCCCGTCG CTACTACCGA TTGGATGGTT TAGTGAGATC
CTCGGATTGG TCTCGA--CA TGGTGGGCAA CC--TCCGCG TCGAT--GTG CCGAGAAGAC
GATCAAACT- TGATC-ATCT AGAGGAAGT- AAAAGTCGTA ACAAGGTTTC CATTACTAAG
CGGAGGAAAA GAAACTAACA A-GGATTCCC CTAGTAACGG CGAGTGAAGC GGGA-AGAGC
CCAGCACCGA ATCCCCCG-G CCTGT----G TC--GGT-GG GAACTGTGGT GTTTAGGCCG
G-CTATTGTC GTCGGTG-CG GAGCGTCC-- ---GAGTCCT CCTGATCGGG G---------
---------C TTCAC----- ---------- -----CCAGA GCGGGTGTCA GGCCTCTAC-
GGCCCTCCGC CCGTCGGCT- --ATC--A-- CCGTC-CTTG GAGTCGGGTT G-TTTGGGAA
TGCAGCCCTA AGTGGGTGGT AAACTCCATC TAAGGCTAAA TACTGACACG AGTCCGATAG
CGGA-CAAGT ACCGTGAGGG AAAGTTGAAA AGAAC-TTTG AAGAGAGAGT TCAAGAGTAC
GTGAAACCGT TTAGAGGCAA ACGGATGGGA CCGCAAA-GT CGACCCGCGG ---AATTCAA
CTCTCTGTTC -G--AGCGCT -CGGCGGCTT GGCGGAC-CG T--------- -A--A-GGCC
GTCTGTTGTC G----AGTCG TTCGTCGGG- ---------- ---------- ---------G
GGCGCACTTT CCGTGGGGAG AGCGCCACGA CCGGTTCTCC GGCAGTCATA AG-CTT----
------CGTG -GGAAGGTAG CTCG------ --CC--TCTC GGGGT-G--- ---AGTG-TT
ATAGCCC-A- --TGGACGTG TTGGCCTGCT G-GGGGACTG AGG------A TCT----CCC
GC----CGCG GCGTCTGGCC C--TG----- ------GCTG CTTCGGTTCG ---TTCG---
ACTG-----G GGGGGACTGT TC-T-CAGTG CCCTCCGACT GCGGG----- -CCGTCT--C
AGACCGGGT- ---------- ------GTCC TCGCGTCAG- ---------- -----CCT--
AG---GGTCT GTGGCGAATC GGTCGGCCCC CTATCCGACC CGTCTTGAAA CACGGACCAA
GGAGTCTAAC ATGTGCGCGA GTCAT-GGGG TTCTACGAAA CCTA---AAG GCGCAATGAA
AGTGA-AGGC TTGC-TGTTC GGCTGGCCTA GGTAGGACGC CG-TCCTTCG -GGGCGG--C
C--------- -GCACTACCG GCCCGTCTCG ATATCGT--T GTATATGAGG -CGGAGCAAG
AGCGTACACG TTGGGACCCG AAAGATGGTG AACTATGCCT GAGTAGGATG AAGTCAGAGG
AAACTCTGAT GGAGGTCCGT AGCGATTCCG TAAGTCTACC GGAGGCAAGG CCCCCAGGAA
ACAGTTGGCT ACCAAGGCCG CCCGTAAGAG CGCCCCAGCC ACTGGTGGAG TTAAGAAGCC
CCATCGTTAC AGGCCCGGTA CCGTCGCTCT CCGTGAGATC CGTCGTTACC AGAAGAGCAC
CGAGCTTCTC ATCCGCAAGC TGCCCTTCCA GCGTCTTGTG CGTGAGATCG CCCAGGATTT
CAAGACCGAT CTGCGCTTCC AGAGCTCTGC CGTCATGGCC CTGCAGGAGG CTAGCGAGGC
TTACCTGGTT GGTCTGTTCG AGGACACCAA CCTGTGCGCC ATCCACGCCA AGCGTAACCC
TTTACTTCAT TTTCGGAGCA TGAGGAGGCC TTCTTGGCAC CTCCATAAGT CTCCTTATTC
GAGCTGAGCT TGGACAACCT GGATCCCTTC TAGGAAGGGA CCAGCTCTAT AACACTATCG
TTACCGCTCA CGCTTTTCTA ATAATTTTCT TTCTTGTTAT ACCAGTATTT ATTGGAGGCT
TTGGAAATTG ACTTATCCCC CTAATACTAG GGGCCCCAGA TATGGCATTT CCCCGGCTAA
ACAATATAAG ATTCTGACTT CTCCCCCCGT CTCTCATTCT TCTAGTTTCT TCCGCAGCTG
TAGAAAAAGG AGTTGGGACA GGATGAACCG TCTATCCCCC TCTCTCTAGA AACCTAGCCC
ACGCTGGTCC CTCTGTAGAC CTAGCAATTT TCTCTCTACA CCTAGCTGGA ATTTCATCAA
TTCTAGGCTC TCTAAACTTT ATCACTACTG TAATCAATAT ACGGTGAAAA GGACTTCGTC
TTGAACGCAT TCCCCTCTTT GTATGAGCCG CTGTAATTAC AACTGTTCTT CTTCTCCTAT
CTCTACCAGT ACTTGCTGGT GCAATTACTA TGCTTCTTAC TGATCGAAAC CTAAATACTG
CATTCTTTGA TCCTGCGGGT GGTGGAGACC CTGTTCTTTA CCAACACCTA TTC-

>Lepidonotus_sublevis
TAGTCATATG CTTGTCTCAA AGATTAAGCC ATGCATGTGT AAGTACAGAC TGTACA----
--------AC GGTGAAACTG CGAATGGCTC ATTAGATCAG TTATGGTTCC TTAGAT---C
GTACAATCCT ACTTGGATAA CTGTGGCAAT TCTAGAGCTA ATACATGC-- TGAC-AATGT
CCGA----CC ---------- ---------- ---TTCAC-- --GGGAGGAC TGCTTTTAT-
TAGATCAAAA CCA-ATCGGG TGCAGTCGTT CGCGGCGGCC CGTC----GT TTTGGTGACT
CTGGATAACT TTGTGCCGAT CGC-ATGGCC TTC----GAG CCGGCGACGA -GTCTTTCAA
ATGTCTGACC TATCAAA-TG TCGATGGTAA GTGACCTGCT TACCATGTTA GTAACGGGTA
ACGGGGAATC AGGGTTCGAT TCCGGAGAGG GAGCATGAGA AACGGCTACC ACATCCAAGG
AAGGCAGCAG GCGCGCAAAT TACCCACTCC CGACACGGGG AGGTAGTGAC GAAAAATAAC
AATACGGGAC TCTT-TCGAG GCCCCGTAA- ---------- --TTGGAATG AGTACACTTT
AAATCCTTTA ACGAGGATCT ATTGGAGGGC AAGT-CTGGT GCCAGCAGCC GCGGTAATTC
CAGCTCCAAT AGCGTATATT AAAGTTGTTG CAGTTAAAAA GCTCGTAGTT GGATCTCGGA
T-CCGGGCGA ATGGTCCGC- ---------- ---------C TCG--CGGCG GTTTACTGTT
-CGATGGCGT CGGTTGGGCT GCCTCTTCGG GGGTTAGTTT CGGTCGGCGT CGTTCCCGGT
CTCTCACCCT CTGGAC-G-- CCCGTC--GG TGCTCTTGAC TGCAGTGCCG GC------GG
CGGCCAGGAC -GTTTACTTT GAAAAAATTA GAGTGTTCCA AGCAGGCGCG TT--CCGCCC
GAATAATGGT GCATGGAATA ATAGAATAGG ACCTCGGTTC TATTTT--GT TGGTTTTCGG
AA-CTAGAGG TAATGATTAA GAGGGACAGA CGGGGGCATT CGTATTGCGG TGTTAGAGGT
GAAATTCTTG GATCGCCGCA AGACGAACTA CTGCGAAAGC ATTTGCCAAG AATGTTTTCA
TTAATCAAAG AACGAAAGTC AGAGGTTCGA AGACGATCAG ATACCGTCCT AGTTCTGACC
ATAAACGATG CCAACTAGCG ATTCGCCGGA GTTGTTTCCA ---TGACCCG GCAGGCAGCT
TC--CGGGAA ACCAAAGTCT TTGGGTTCCG GGGGAAGTAT GGTTGCAAAG CTGAAACTTA
AAGGAATTGA CGGAAGGGCA CCACCAGGAG TGGAGCCTGC GGCT-TAATT TGACTCAACA
CGGGAAAACT CACCTGGCCC GGACACCGTT AGGATTGACA GATTGAGAGC TCTTTCTCGA
TTCGGTGGGT GGTGGTGCAT GGCCGTTCTT AGTTGGTGGA GCGATTTGTC TGGTTAATTC
CGATAACGAA CGAGACTCTG ACCTGCTAAC TAGTCCGTCG ATTTACCCGA TTCGTTCGGT
CGACGCCG-- ----ACTTCT TAGAGGGACA AGTGGC---- GTACAGCCAC ---GCGAGAT
```

```
TGAGCAATAA CAGGTCTGTG ATGCCCTTAG ATGTTCGGGG CCGCACGCGC GCTACACTGA
AGGAATCAAC GTGT-----T CATCGTCCTG GT-CCGGAAG GATC------ ----------
---------- -----GGGTA ACCCGTTGAA CCTCCTTCGT GCTAGGGATT GGGGCTTGTA
ATTCTTCCCC ATGAACGAGG AATTCCCAGT AAGCGCGTGT CATAAGCACG CGTTGATTAC
GTCCCTGCCC TTTGTACACA CCGCCCGTCG CTACTACCGA TTGAATGGTT TAGTGAGATC
CTCGGACCGG CCCCGG--CA CGGCGGGCAA CC--GTCGAG CCGTG--GCG CCGGGAAGAC
GATCGAACT- TGATC-ATTT AGAGGAAGT- AAAAGTCGTA ACAAGGTTTC CATCACTAAG
CGGAGGAAAA GAAACTAACC ATGGATTCCC CTAGTAACGG CGAGTGAAGC GGGA-AGAGC
CCAGCACCGA ATCCCCTGTC GCTGC----G GC--AGT-GG GAACTGTGGT GTTTAGGACG
A-TCACTGGC GCGTCGT-GC TCCCGTCC-- ---GAGTCCT CCTGATCGGG G---------
---------C CTTTC----- ---------- -----CCATA GCGGGTGTCA GGCCTCTA--
GTCTAGGCGA GGGCCGGCGT GCCTCTAA-- ACGTC-CTTG GAGTCGGGTT G-TTTGGGAA
TGCAGCCCAA AGTGGGTGGT AAACTCCATC TAAGGCTAAA TACTGACATG AGACCGATAG
CGGA-CAAGT ACCGTGAGGG AAAGTTGAAA AGAAC-TTTG AAGAGAGAGT TCAAGAGTAC
GTGAAACCGC TCAGAGGCAA ACTGATGGGA CCGCAAA-GT CGCCTCGTGG ---AATTCAA
CTCTTCCGAG GG--CTCGGG GCGTTCAGAC GTAGATC-CT C--------- -ATGG-GACT
GCTTTGGGCG ---------- TTCGCGGGC- ---------- ---------- ---TCCGGAG
GGCGCCCTTT CCACGAGGAG AGCGCCACGA CCGGTCCGTT GGCGGCCAGA AGTCCCAGAG
AAAGGTAGCG -GGAACGATT TCGG------ --TCGCCTTC CGTG------ --------TT
ATAGTCT-C- --TGGGTTCG GAGGCCCGCC G-GCGAACCG AGG------A ACA---GCAT
GC----CGCC GCTTCCGCCT CCTTCGGGAT GGCGCGGGCC CCCGCGTCGG ---TTTG---
TCGGGTCGAG GATGGACTGT TT-A-CAGTG TGTTCCGGCT GCCAGGCTTA TCGGCTG--T
GGGTGTGCT- ---------- ------CCCG TTTTGGAAG- ---------- -----CCT--
AG---GGTCC GTGGCGAATC GATCGGCACC CCATCAGACC CGTCTTGAAA CACGGACCAA
GGAGTCTAAC ATGTGCGCAA GTCAT-TGGG CTCTACGAAA CCCA---CAG GCGCAATGAA
AGTGACAGGG CGTT-CGC-- ----GTCCGT GGCAGGATCC GCCGCCCCCG --GGCGGTGG
C--------- -GCACTGCTG GCCCGTCTCG GTCGCCA--C GTCGACGAGG -CGGAGCAAG
AGCGTACACG TTGGGACCCG AAAGATGGTG AACTATGCCT GAGTAGGACG AAGTCAGGGG
AAACCCTGAT GGAGGTCCGT AGCGATTC?? ?????????? ?????????? ??????????
?????????? ?????????? ?????????? ?????????? ?????????? ??????????
?????????? ?????????? ?????????? ?????????? ?????????? ??????????
?????????? ?????????? ?????????? ?????????? ?????????? ??????????
?????????? ?????????? ?????????? ?????????? ?????????? ??????????
?????????? ?????????? ?????????? ?????????? ?????????? ?????-----
---------T TTTTGGTAC- TGATCAGGTC TTTTAGGAAC TTCTATAAGA CTTCTAATTC
GTGCTGAGCT AGGACAACCT GGATCCTTGC TTGGCAGAGA CCAATTATAT AATACTATTG
TTACAGCTCA CGCTTTTTTA ATAATTTTTT TTCTTGTAAT ACCAGTCCTA GTAGGAGGAT
TTGGTAATTG ATTAATTCCA CTAATATTAG GTGCTCCTGA TATAGCTTTT CCACGATTAA
ATAATATAAG ATTTTGATTA CTTCCCCCCT CACTAATTCT TTTACTCTCA TCAAGAGCTG
TAGAAAAAGG GGTGGGAACA GGATGGACAG TTTATCCGCC TCTTGCTTCT AATATTGCAC
ATGCTGGGCC TTCCGTTGAT CTAGCTATTT TTTCTCTTCA TATTGCAGGG GTCTCTTCTA
TTTTAGGAGC CCTTAATTTT ATTACTACAG TTGTTAATAT ACGATACAAA GGATTACGTT
TAGAACGTGT TCCTCTTTTT GTATGAGCAG CTAAAATTAC AGCAATTCTT TTATTACTTT
CCTTACCAGT TCTAGCTGGG GCTATTACGA TACTTTTAAC TGACCGTAAC CTAAATACCG
CTTTCTTTGA CCCTGCCGGT GGAGGGGATC C--------- ---------- ----

>Siphonosoma_cumanense
---TCATATG CTTGTCTCAA AGATTAAGCC ATGCATGTGT AAGCACAGGC CTTTAC----
--------AT GGTGAACCTG CGAATGGCTC ATTAGATCGG CTATTATTTA TTAGAG---C
ATACTATCCT ACTTGGATAA CTGTGGCAAT TCTAGAGCTA ATACATGC-- AACA-CGGCT
CCGACCTCCG ---------- ---------- ---------- --GGGAAGAG CGAGTTTAT-
TAGATCAAAA CCA-ATCTG- ------GCCG CAA----GGC CAGAGG--TT GTTGGTGACT
CTGGATAACT TTGGTCTGAT CGC-ACGGCC TTG----GCG CCGGCGACGC -ATCTTTCAA
GTGTCCGCCC TATCAAC-TG TCGTTGGTAA GGTATCTGCT TACCAAGGTT GTAACGGGTA
ACGGGGAATC AGGGTTCGAT TCCGGAGAGG GAGCATGAGA AACGGCTACC ACATCCTAGG
AAGGCAGCAG GCGCGCAAAT TACCCACTCC CGGCACGGGG AGGTAGTGAC GAAAAATAAC
AATACGGGAC TCTT-ACGAG GCCTCGTAA- ---------- --TTGGAATG AGTACACTTT
AAATCCTTTA ACGAGGATCT ATTGGAGGGC AAGT-CTGGT GCCAGCAGCC GCGGTAATTC
CAGCTCCAAT AGCGTATATT AAAGCTGCTG CAGTTAAAAA GCTCGTAGTT GGATCTCGGG
C-GCCGGCGG GCGGTTCGC- ---------- ---------T TCA--CGGCT TCTTACTGCC
-CG------- ---------- ---------- ---------- ---------- ---CCACGGC
CCT--ACCAG CCGGCT-CAC CCCG----GG TGCTCTTGGT TG-AGTGCCT GG------GG
CGTCCGGAAG -CTTTACTTT GAAAAAATTA GAGTGCTCAA AGCAGGCG-- CA--AGGCCT
GTATAATGCT GCATGGAATA ATGGAATAGG ACCTCGGTTC TATTTT--GT TGGTTTTCGG
AACCCCGAGG TAATGATTAA GAGGGACAGA CGGGGGCATT CGTATTACGG CGTTAGAGGT
GAAATTCTTG GATCGCCGTA AGACGGACAA CTGCGAAAGC ATTTGCCAAG AATGTTTTCA
TTAATC-AAG AACGAAAGTC AGAGGTTCGA AGACGATCAG ATACCGTCGT AGTTCTGACC
ATAAACGATG CCAACTGGCG ATCGGCGGCG GTTGATTTAG ---TGACTCC GCCGGCAGCC
AC--CGGGAA ACCTAAGTCT TTAGGTTCCG GGGGAAGTAT GGTTGCAAAG CTGAAACTTA
AAGGAATTGA CGGAAGGGCA CCACCAGGAG TGGAGCCTGC GGCT-TAATT TGACTCAACA
CGGGAAAACT CACCCGGCCC GGACACTGTA AGGATTGACA GATTGAGAGC TCTTTCTTGA
TTCGGTGGGT GGTGGTGCAT GGCCGTTCTT AGTTGGTGGA GCGATTTGTC TGGTTAATTC
CGATAACGAA CGAGACTCTA GCCTGCTAAC TAGCCAGCCG GT------CC GTCAAG--GC
CGGCG-CAGA ----GCTTCT TAGAGGGACA AGTGCA---- TGGAATGCAC ---ACGAGAT
TGAGCAATAA CAGGTCTGTG ATGCCCTTAG ATGTTCGGGG CCGCACGCGC GCTACACTGA
AGGCAGCAGC AGGT-----G TGTTTTCCTG GC-CCGAGAG GGTC------ ----------
```

```
---------- -----GGGTA ACCC-TTGAA CCGCCTTCGT GCTAGGGATC GGGGCTTGTA
ATTATTCCCC GTGAACGAGG AATTCCCAGT AAGCGCGAGT CATAAGCTCG CGTTGATTAC
GTCCCTGCCC TTTGTACACA CCGCCCGTCG CTACTACCGA TTGGAAGGTT TAGTGAGGCC
CTCGGATTGG TCGCTG--CT GGTTGGGCGA CC--GGCCGG CAGT---GAA CCGAGAAGAA
GGCCAAACT- TGACT-TTCT AGAGGAAGT- AAAAGTCGTA ACAAGGTTTC C---------
---------- ---------- ---------- ---------- --------GT GGCA-ATAGC
CCAGCGCTGA AGCTCCCGCC CCAAC---GG GCGTGGC--- GCAATGTAGC GTTTGGGACG
C-GCCGCGCC AGGCTGG-CC GACCGCCC-- ---AAGTCCT CCTGATCGGG G---------
---------C ATCAC----- ---------- -----CCGCG GCGGGTGTCA GGCCCATAG-
GGCGGTCCG- -AGGCCGGG- --CCAGGT-- GCGTC-CCCG GAGTAGGGTT G-TTTGTGAA
TGCAGCCCGA AGTGGGTGGT AAACTCCATC TAAGGCTAAA TACTGGCACG AGTCCGATAG
CGGA-CAAGT ACCGTGAGGG AAAGTTGAAA AGAAC-TTTG AAGAGAGAGT TCAATAGTAC
GTGAAACCGT TCAGAGGGAA ACGGACGGGA CCG-GTAAGT CGACCCGGGG ----ATTCAA
CCCGTTGGCT CGGCCCGGCG GCGGCGGAAG GACCCCTTCA C--------- -GGGG-GACA
CCGCCCGCCG CGTCAGGGCT CGTGGCCGC- ---------- ---------- ---------G
GGCGCACTTC CC-CGGGGAG AGCGCCGGGA CCGGCTGGCG GGCGGCCAGA AGGCCG----
------GGAG -CGTTGGTGT CCCC-----C TCCCCACTCG GGAGGGG--- ---GTGCCTC
GCGCCCC-GT GCCGGGCCCG CCCGCCGGCC GAGGGGACCG CCG------- -----CGCCC
GCGTTACGGC GCCGGGGGTC GC-------- ------GGGC CGGGCGACGG GGACTCA---
ACCG----AG GCGGGACTGT TCGCGCAGTG CCGCCCGACC GCCCC----- -CGCGCCAGA
GGCCCGCGC- ---------- ------GCAC CCCGGTAGGG ---------- -----CGCAC
AG---GGTCC CCGGCGAGCC AGCCGGCACC CCGTCCGACC CGTCTTGAAA CACGGACCAA
GGAGTCTAAC ATGTGCGCGA GTCAG-TGGG CCTCACGAAC CCCA---AAG GCGCAATGAA
AGTGAAAGGC CGGCTTGCCC GGC---CCTA GGTGGGATCC GG-CCGCCCG GCGGCCG--G
C--------- -GCACCACCG GCCCGTCTCG CACGGTT-GC GTTCCGGGAG GCGGAGCAAG
AGCGTACACG TTGGGACCCG AAAGATGGTG AACTATGCCT GAGCAGGGTG AAGCCAGAGG
AAACTCTGGT GGAAGTCCGT AGCGATTCCG TAAGTCTACA GGAGGCAAGG CCCCAAGGAA
GCAGCTGGCC ACCAAAGCTG CCAGAAAGTC AGCACCAGCC ACCGGTGGTG TGAAGAAGCC
TCACCGTTAC AGGCCAGGCA CAGTTGCTCT CCGTGAGATC CGTCGTTACC AGAAGAGCAC
TGAGCTGCTC ATCCGCAAAC TGCCATTCCA GCGTTTRGTG CGTGAGATTG CACAGGACTT
CAAGACTGAC CTGCGCTTCC AGAGCTCAGC TGTCATGGCC CTGCAAGAGG CATCTGAGGC
CTACCTGGTC GGCCTCTTTG AGGACACCAA CTTGTGCGCC ATCCACGCCA AGCGT-----
-GTACNTTAT TTTAGGAATT TGAGCAGGTC TATTAGGAAC ATCTATAAGN CTACTAATTC
GAGCAGAACT GGGTCAGCCC GGATCCCTCT TAGGAAATGA TCAGCTTTAT AATGTTATCG
TAACTGCGCA TGCATTTTTA ATGATTTTTT TCCTAGTAAT ACCAGTCTTA ATTGGAGGTT
TTGGGAACTG ACTTATTCCT TTAATAGTAG GAACCCCTGA TATAGCTTTT CCTCGCTTAA
ATAATATAAG ATTCTGACTT CTTCCTCCTG CTCTCTTCTT ACTCGTAGCT TCCGCTATAG
TAGAACAAGG AGTCGGTACG GGTTGAACTG TATATCCACC TTTATCAGAT AATATTGCTC
ATACAGGGCC TTCTGTAGAT TTAGCAATTT TCTCACTCCA CTTAGCAGGA GCAAGCTCTA
TCTTGGGAGC CCTAAATTTT ATTAGAACAG TTGCTAATCT TCGGCACCGA GGAGTTACGT
ACGAACGGCT TCCTTTATTT ATTTGAGCTG TTTTTATTAC CGTTATTCTT CTTTTGCTAG
CTCTCCCTGT TTTAGCCGGG GCAATTACTA TGCTCTTAAC AGATCGGAAT CTAAATACAT
CTTTTTTTGA CCCAAGGGGA GGGGGCGACC CGATTCTCTT TAGACATCTC ----

>Littorina_littorea
---TCATATG CTTGTCTCAA AGATTAAGCC ATGCATGTCT AAGTTCACAC CCTCGT----
--------AC GGTGAAACCG CGAATGGCTC ATTAAATCAG TCGAGGTTCC TTAGAT---G
ATCCCAATCT ACTTGGATAA CTGTGGTAAT TCTAGAGCTA ATACATGC-- CAAC-CAGCT
CCGACCCGTT ---------- ---------- ---ACTG--- --GGAAAGAG CGCTTTTAT-
CAGTTCAAAA CCA-GTCGG- -----GCCCG TTAAACGGTC CGTC-----C CTTGGTGACT
CTGGATAACT TTGTGCCGAT CGC-ATGGCC T-C----GAG CCGGCGACGC -ATCTTTCAA
ATGTCTGCCC TATCAAA-TG ACGATGGTAC GTGATCTGCC TACCATGTTG GCAACGGGTA
GCGGGGAATC AGGGTTCGAT TCCGGAGAGG GAGCATGAGA AACGGCTACC ACATCCAAGG
AAGGCAGCAG GCGCGCAACT TACCCACTCC TGGCACGGGG AGGTAGTGAC GAAAAATAAC
AATACGGAAC TCTT-TTGAG GCTCCGTAA- ---------- --TTGGAATG AGTACACTTT
AAACCCTTTA ACGAGGATCT ATTGGAGGGC AAGT-CTGGT GCCAGCAGCC GCGGTAATTC
CAGCTCCAAT AGCGTATACT AAAGTTGTTG CGATTAAAAA GCTCGTAGTT GGATCTCAGG
C-ATGGGCGC ACGGTCCGC- ---------- ---------C TCG--CGGC- GGTCACTGTG
-TG------- ---------- ---------- ---------- --------TA TGTTTCCCAT
CCTACGCTTC CCGGTTGTTA GCCCAT--GG TGCTCTTCAT TG-AGCGTTT TG------GG
TGGCCGGAAC -GTTTACTTT GAAGAAATTA GAGTGTTCAA AGCAGGCACG TC----GCCT
GAATAATGGT GCATGGAATA ATGGAATAGG ACCTCGGTTC TATTTT--GC TGGTTTTCGG
AA-CACGAGG TAATGATTAA GAGGGACAGA CGGGGGCATC CGTATTGCGG TGTTAGAGGT
GAAATTCTTG GATCATCGCA AGACGAACTA CTGCGAAAGC ATTTGCCAAG AATGTTTTCA
TTAGTC-AAG AACGAAAGTC AGAGGTTCGA AGACGATCAG ATACCGTCGT AGTTCTGACC
ATAAACGATG CCAACTAGCG ATCCGCTGGT GTTGCTTCAT ---CGACTCT GCGGGCAGCT
TC--CGGGAA ACCAAAGTCT ATGGGTTCCG GGGGAAGTAT GGTTGCAAAG CTGAAACTTA
AAGGAATTGA CGGAAGGGCA CCACCAGGAG TGGAGCCTGC GGCT-TAATT TGACTCAACA
CGGGGAAACT CACCCGGTCC GGACACTGTA AGGATTGACA GATTGATAGC TCTTTCTTGA
TTCGGTGGGT GGTGGTGCAT GGCCGTTCTT AGTTGGTGGA GCGATTTGTC TGGTTAATTC
CGATAACGAA CGAGACTCTA GCCTACTAAA TAGTTCGCCG AT------CC TTTATGC-GT
CGGCGTCA-- ----ACTTCT TAGAGGGACA AGTGGC---- GTTTAGCCAC ---ACGAGAT
TGAGCAATAA CAGGTCTGTG ATGCCCTTAG ATGTCCGGGG CCGCACGCGC GCTACACTGA
AGGAATCAGC GTGG-----C TATCTCCCTG GT-CCGAGAG GATT------ ----------
---------- -----GGGAA ACCCGTTGAA TCTCCTTCGT GATAGGGATT GGGGCTTGAA
ATTCTTCCCC ATGAACGAGG AATTCCCAGT AAGCGCGAGT CATCAGCTCG CGTTGATTAC
```

```
GTCCCTGCCC TTTGTACACA CCGCCCGTCG CTACTACCGA TTGAACGGTT TAGTGAGGGC
CTCGGATTGG TCTCGG---C CCGCCCTTCA CCGGGCGGCG CCGTT---GG TCGAGAAGAC
GCTCGAACT- TGATC-GTTT AGAGGAAGT- AAAAGTCGTA ACAAGGTTTC C-----TAAG
---------- --AACTAACA --GGATTCCC TCAGTAACGG CGAGTGAAGC GGGA-TCAGC
CCAGCACCGA ATCCCCCA-G TCTTT----G GCTTGGC-GG GAACTGTGGT GTATGGGACG
C-CAACT-TG TCGTCCGACC GGGTGCCG-- ---AAGTCCT CCTGATCGGG G---------
---------C CTCTC----- ---------- -----CCAGA GCGGGTGTCA GGCCTTTACT
GGTGCCTGGT CGGTCGGCT- ----GCGA-- GCGTC-TCTC GAGTCGGGTT GTTTTGGGAA
TGCAGCCCTA AGCGGGTGGT AAACTCCATC TAAGGCTAAA TACTGGCACG AGTCCGATAG
CGGA-CAAGT ACCGTGAGGG AAAGTTGAAA AGAACTTTTG AAGAGAGAGT TCAACAGTAC
GTGAAACCGC CTAGAGGTAA ACGGGTGGAT CCGCAAA-GT CGGCCCGCGG ---AATTCAG
CTCGGATGGC TG--GCGCGT GCGCCGGGC- AAGAGAT-CT G--------- ------AACG
GACTCTCCTG GTGAACGACG CTGGTCGGC- ---------- ---------- ---C-----G
TGTGCACTTT CCGCGGGCAG AGCGCCACGA CCGGTTCTCG GGTGGTCAGA AGGC------
------GGCG AGGAAGGTAG GTGG------ --GC-GCTTC GGCGCCT--- ---ACTG-TT
ATAGCCTCG- --CCTGACC- -CGACCCACC T-GGGGACCG AGG------A G-------CC
GC----CGTC GGTGTAGGCC GCCTC----- ------GCTT TCTTGGGATG ---TTCG---
ACTG-----G CGGAGACTGG GC-AACCGTG TCTGCCGACC GC-------- -TTCCTGAGA
TGGACTGGG- ---------- ------GTGG GCCCGCCGG- ---------- -----CAC--
AG---GGTCA GTGGCGAATC GGTCGGCCCT CCACCCGACC CGTCTTGAAA CACGGACCAA
GGAGTCTAAC ATGCGCGCGA GTCGT-TGGG TAGTACGAAA CCCG---AAG GCGAAGTGAA
AGCGA-GGGC CGTC---TCT GACGAGCTCA GGTGGGATCC CTCTCTTCCG --GGAGGGGG
C--------- -GCACCACCG GCCCGTCTCG TCCGCGT--T GTCGGTGAGG -CGGAGCATG
AGCGCGCACG TTGGGACCCG AAAGATGGTG AACTATGCCT GAGTAGGACG AAGCCAGAGG
AAACTCTGGT GGAGGTCCGC AGCGATTCCG TAAATCCACC GGAGGAAAAG CTCCTCGCAA
ACAGCTGGCA ACCAAGGCCG CTCGCAAAAG CGCCCCTGCC ACTGGAGGAG TCAAGAAACC
TCACCGTTAC AGGCCTGGAA CTGTGGCTCT TCGTGAGATC CGTCGTTACC AGAAGAGCAC
CGAGCTCCTG ATCCGCAAGC TGCCCTTCCA GCGTCTGGTG CGCGAAATCG CCCAGGACTT
CAAGACAGAC CTGCGCTTCC AGAGCTCTGC CGTCATGGCT CTGCAGGAGG CCAGCGAGGC
TTACCTGGTC GGTCTCTTTG AGGACACCAA CCTGTGCGCC ATCCACGCCA AGCGT-----
---------- ---------- --ATCTGGGC TTGTTGGTAC TGCCTTAAGT CTACTTATTC
GGGCTGAATT AGGTCAACCT GGCGCTCTCC TGGGAGATGA CCAGCTGTAC AACGTTATCG
TTACAGCCCA CGCCTTTGTA ATAATTTTCT TTCTTGTTAT GCCTATAATA ATTGGTGGGT
TTGGAAATTG ACTTGTCCCC TTAATATTAG GAGCACCCGA TATAGCATTC CCTCGCTTAA
ATAACATAAG CTTTTGATTA CTCCCCCCCG CCTTGTTGTT GTTACTATCT TCTGCTGCGG
TAGAAAGTGG TGCAGGAACG GGTTGAACTG TATATCCTCC TTTATCCGGA AATTTAGCCC
ATGCCGGAGG CTCTGTGGAC TTAGCCATTT TCTCTCTTCA TTTGGCCGGT GTCTCATCTA
TTTTAGGGGC CGTAAATTTT ATTACAACTA TTATTAATAT ACGATGACGA GGGATGCAAT
TCGAGCGATT ACCTCTTTTT GTTTGATCTG TAAAAATTAC AGCCATTCTT TTACTTTTAT
CCCTTCCAGT TTTAGCAGGA GCCATTACAA TATTGTTAAC TGATCGAAAT TTTAACACTG
CCTTCTTCGA TCCTGCTGGG GGTGGAGATC CTAT------ ---------- ----
```

```
>Solemya_velum
---TCATATG CTTGTCTCAA AGATTAAGCC ATGCATGTCT AAGTGCACAC TTTCAC----
--------AT AGTGAAACCG CGAATGGCTC ATTAAATCAG TTGATGTTTA TTAGAT---C
GTACAATCCT ACTTGGATAA CTGTGGTAAT TCTAGAGCTA ATACATGC-- AACC-AAGCT
CCGA-----C ---------- ---------- ---CTCGTGA GAGGGAAGAG CGCTTTTAT-
TAGATCAAAA CCA-ATCGG- ----TTCTTT C---GGGAGC CGT-----CT GTTGGTGACT
CTGGATAACT TTGTGCTGAT CGC-ACGGCC A-C----GAG CCAGCGACGT -ATCTTTCAA
ATGTCTGCCC TATCAAC-TG TCGATGGTAG GAGATGTGCC TACCATGGTC GTAACGGGTA
GCGGGGAATC AGGGTTCGAT TCCGGAGAGG GAGCATGAGA AACGGCTACC ACATCCAAGG
AAGGCAGCAG GCGCGCAAAT TACCCACTCC TGGCACGGGG AGGTAGTGAC GAAAAATAAC
AATACGGGAC TCGT-TCGAG GCCCCGTAA- ---------- --TTGGAATG AGTACACTCT
AAATCCTTTA ACGAGGATCT ATTGGAGGGC AAGT-CTGGT GCCAGCAGCC GCGGTAATTC
CAGCTCCAAT AGCGTATATT AAAGTTGTTG CAGTTAAAAA GCTCGTAGTT GGATCTCGGG
T-TTGGGCTG GCGGTCCGC- ---------- ---------C TAG--CGGCG GTTTACTGCC
ACG------- ---------- ---------- ---------- ---------- ---TCCCGAC
CT---ACCTG CCGGCC-C-- ACCCTT--GA TGCTCTTGAC TG-AGTGTCT CG------GG
CGACCGGAAC -GTTTACTTT GAAAAAATTA GAGTGTTCAA AGCAGGCCT- AT--CGGCCT
GAATAATGGT GCATGGAATA ATGGAATAGG ACCTCGGTTC TATTTT--GT TGGTTTTCGG
AA-CTGGAGG TAATGATTAA GAGGGACAGA CGGGGGCATT CGTATTACGG TGTTAGAGGT
GAAATTCTTG GATCGCCGTA AGACGAACTA CTGCGAAAGC ATTTGCCAAG AATGTTTTCA
TTAATC-AAG AACGAAAGTC AGAGGTTCGA AGACGATCAG ATACCGTCGT AGTTCTGACC
ATAAACGATG CCGACTAGCG ATCCGCCGTA GTTGCTTCAA ---TGACTCG GCGGGCAGCT
TC--CGGGAA ACCAAAGTCT TTGGGTTCCG GGGGAAGTAT GGTTGCAAAG CTGAAACTTA
AAGGAATTGA CGGAAGGGCA CCACCAGGAG TGGAGCCTGC GGCT-TAATT TGACTCAACA
CGGGAAAACT CACCCGGCCC GGACACTGTA AGGATTGACA GATTGAGAGC TCTTTCTTGA
TTCGGTGGGT GGTGGTGCAT GGCCGTTCTT AGTTGGTGGA GCGATTTGTC TGGTTAATTC
CGATAACGAA CGAGACTCTA GCCTACTAAA TAGTTCGCCG AT-------- TCGATATAGT
CGGCG-CA-- ----ACTTCT TAGAGGGACA AGTGGC---- TTYTAGCCAC ---ACGAGAT
TGAGCAATAA CAGGTCTGTG ATGCCCTTAG ATGTTCGGGG CCGCACGCGC GCTACACTGA
AGGAATCAAC GTGC-----A -TTTGCCCTT GC-CCGGAAG GGTT------ ----------
---------- -----GGGTA ACCCGTTGAA CCTCCTTCGT GCTAGGGATT GGGGCTTGTA
ATTATTCCCC ATGAACGAGG AATTCCCAGT AAGCGCGAGT CATAAGCTCG CGTTGATTAC
GTCCCTGCCC TTTGTACACA CCGCCCGTCG CTACTACCGA TTGAGCGGTT TAGTGACATC
CTCGGACTGT TCCCGA--GA CGG-GGGCAA CC--TCGAC- TCGGT--TGG ACGGAAAAC
```

```
GATGGAACT- TGATC-GCTT AGAGGAAGT- AAAAGTCGTA ACAAGGTTTC CATCACTAAG
CGGAGGAAAA GAAACTAACA A-GGATTCCC TCAGTAACGG CGAGTGAAGC GGGA-AAAGC
CCAGCACCGA ATCCCGCA-G CTCAT----G GC--TGCAGG GACCTGTGGT GTTTGGATCG
T-CGAGTGTC GATGCGT-TC GGGCTCCT-- ---AAGTCCT CCTGATCGGG G---------
---------C TTCAT----- ---------- -----CCATA GCGGGTGTCA GGCCTTTACA
GGCGCCCGAC GCTTCGACT- ---CCGAA-- GCGAT-CTAG GAGTAGGGTT G-TTTGGGAA
TGCAGCCCGA AGTGGGTGGT AAACTCCATC TAAGGCTAAA TACAGACACG AGTCCGATAG
AGGA-CAAGT ACCGTGAGGG AAAGTTGAAA AGAAC-TTTG AAGAGAGAGT TCAAGAGTAC
GTGAAACCGC CTAGAGGTAA ACGGGTGGAA CCGCAAA-GT CTGCCCGGGG ---AATTCAA
CTTGCCGAGC GT--CCGGCC TTCGGGGGTG TGCGGATCCG T--------- -A--AAGACC
GTCCCC---- CTGTTCGACC GGTGCCGGC- ---------- ---------- ---------T
AGTGCACTTT CTCCGGGCGG AGCGCCACGA CCGGTTTTTC GGCGGCCAGA AG-GCG----
------ATCG -GGAAGGTGA CTAC------ --TGCTCTTG CGGGCAG--- ---TAGTGTT
ATAGCCCGG- --TCGATACT TGGGCACGTC G-GGAGACCG AGG------A A-----GGTT
GC----CGCG GCGAGCGGGT TCTCG----- ------GCCC TCTTCTCGCA ---TTCG---
ACTG-----G TGCTCACTGT TC-TGCAGTG TGAACCGACT GTGTG----- -GGTCGA--T
CGGTCGGGA- ---------- ------TTTC CGTCGTCGC- ---------- -----CTC--
-G---GGTCA GTGGCGAATC GGTCGGTCCT CCACCCGACC CGTCTTGAAA CACGGACCAA
GGAGTCTAAC ATGTGCGCAA GTCAT-GGGG TCTATCGAAA CCTA---AAG GCACAATGAA
AGTGA-AGGC CGGCTCGTT- --TCGGCCTA GGCGGGATCC CCTTCCTAC- --GGAGGGGG
C--------- -GCACCGCTG GCCCGTCTCG TCCGACT--C GTCGGTGAGG -CGGAGCAAG
AGCGTACACG TTGGGACCCG AAAGATGGTG AACTATGCCT GAGTAGGACG AAGCCAGAGG
AAACTCTGGT GGAGGTCCGT AGCGATTCCG TAAGTCCACC GGTGGCAAAG CCCCAAGAAA
GCAGTTGGCT ACCAAGGCCG CACGTAAAAG TGCCCCAGCC ACAGGAGGTG TGAAGAAACC
ACACAGATAC AGGCCCGGAA CCGTCGCCTT GAGAGAAATC CGTCGTTACC AGAAGAGCAC
TGAACTTCTG ATCAGGAAAC TTCCATTCCA GCGACTTGTT CGTGAGATCG CCCAGGATTT
CAAGACCGAT CTGCGATTCC AGAGCTCAGC TGTCATGGCT TTGCAAGAGG CTAGCGAAGC
ATACCTCGTT GGTCTTTTCG AGGATACCAA CTTGTGCGCC ATCCACGCCA AAAGA-----
---------- ---------T TGAGCCGGTA TAGTTGGAAC ATCTCTTAGT CTCTTAATTC
GAGCTGAACT AGGACAGCCT GGAGCCCTTT TAGGGGACGA CCAACTTTAT AACGTAATCG
TGACAGCACA TGCATTTATT ATGATTTTCT TCCTAGTAAT ACCAATAATA ATAGGAGGGT
TTGGAAATTG ATTTGTTCCT ATAATACTAG GGGCTCCAGA CATAGCATTC CCACGAATAA
ATAATATGAG ATTCTGACTT TTACCTCCTG CCTTAACTCT TCTATTGGGG TCAGCTGCTG
TAGAAAGGGG GGCTGGAACG GGGTGAACAG TATACCCACC ACTTTCTGGA AATCTAGCTC
ACGCAGGTGC CTCAGTAGAT CTAACAATTT TCTCACTACA TTTAGCGGGT GCCTCATCAA
TTATAGCATC AATTAATTTT ATTACAACAG CAATTAACAT ACGATCCCGA GGAATACGAT
TCGAACGAAT ACCTTTATTT GTTTGATCTA TTAAAATTAC AGCTGTTTTA CTTCTTCTTT
CACTTCCAGT TCTAGCCGGA GCAATTACTA TACTATTAAC AGACCGAAAT TTTAACACAT
CATTCTTCGA TCC------- ---------- ---------- ---------- ----
```
